# Proteomic Profile of Skeletal Muscles and Liver in a Dexamethasone-Induced Atrophy Model: *Insights* into the Role of β-Hydroxy-β-Methylbutyrate on Contractile and Metabolic Protein

**DOI:** 10.1007/s12013-026-01995-4

**Published:** 2026-01-22

**Authors:** Talita Mendes Oliveira Ventura, Cintia Kazuko Tokuhara, Mizael Pereira, Cleuber Rodrigo de Souza Bueno, Idvaldo Aparecido Favaretto-Junior, Iris Jasmin Santos German, Marcelie Priscila de Oliveira Rosso, Aline de Lima Leite, André Luís Shinohara, Rogério Leone Buchaim, Marília Afonso Rabelo Buzalaf, Jesus Carlos Andreo, Rodrigo Cardoso de Oliveira

**Affiliations:** 1 Department of Biological Sciences, Discipline of Biochemistry, Bauru School of Dentistry, University of São Paulo FOB-USP, Bauru, São Paulo Brazil; 2 Human Genetics Program, Sanford Burnham Prebys Medical Discovery Institute, La Jolla, California USA; 3 Department of Biological Sciences, Universidade do Sagrado Coração, Bauru, São Paulo Brazil; 4 Department of Biological Sciences, Discipline of Anatomy, Bauru School of Dentistry, University of São Paulo FOB-USP, Bauru, São Paulo Brazil

**Keywords:** Skeletal muscle. Muscle atrophy. Liver. Dexamethasone. Leucine

## Abstract

First study to characterize the proteomic alterations induced by β-Hydroxy-β-Methylbutyrate in combination with Dexamethasone.

Evaluation of β-Hydroxy-β-Methylbutyrate in a severe glucocorticoid-induced muscle atrophy model.

Analysis focused on white (fast-twitch) skeletal muscle, which is preferentially affected by glucocorticoid-induced atrophy.

**Supplementary Information:**

The online version contains supplementary material available at 10.1007/s12013-026-01995-4.

## Abstract (unstructured)

Although some effects of glucocorticoids are necessary for metabolic responses during stress, chronic exposure to high levels of these hormones can cause side effects such as hyperglycemia, insulin resistance, and muscle atrophy. Elevated glucocorticoid levels are observed in several clinical conditions, and their exogenous administration, such as high-dose dexamethasone treatments, is included in many clinical protocols. In this context, the supplement β-Hydroxy-β-methylbutyrate (HMB) emerges as a promising approach to counteract side effects its anticatabolic action. Therefore, the aim of this study was to evaluate the protein profile in the muscles and liver of rats treated with HMB in response to a model of severe muscle atrophy induced by dexamethasone. A total of 24 male Wistar rats, 60 days old, were used and distributed into the following groups: (1) Placebo Experimental Group (PEG), *n* = 8, treated only with saline solution; (2) Dexamethasone Experimental Group (DEG), *n* = 8, treated with intraperitoneal Dexamethasone injection (1 mg/kg/day); and (3) Dexamethasone + HMB Experimental Group (DEHG), *n* = 8, treated with intraperitoneal Dexamethasone injection (1 mg/kg/day) and gavaged with HMB (0.3 g/kg/day). After 10 days of treatment, the animals were euthanized for collection of the Soleus, Extensor Digitorum Longus (EDL) muscles, and liver, followed by protein processing and identification. In the Soleus muscle, when comparing DEG vs. PEG, proteins such as *Myosin heavy chain 2*, *Myosin-4*, *Myosin-*6, and *Myosin-7* showed decreased expression in DEG compared to PEG. Comparing PEG vs. DEHG also showed a decrease in myosin expression in DEHG, but only *Myosin-6* and *Myosin-7* were reduced among the identified isoforms. In the EDL muscle, both DEG and DEHG showed reduced levels of all identified myosin subtypes. However, when comparing DEG and DEHG, the only myosin isoform preserved in DEHG compared to DEG was *Myosin heavy chain 2*. In the liver, dexamethasone treatment impaired glucose uptake, altered the Cytochrome P450 system, and reduced mitochondrial function, as reflected by reduced ATP levels. In conclusion, dexamethasone induced the loss of contractile proteins (myosins) in both muscles. The HMB dosage used in this study partially prevented myosin loss in the Soleus muscle but did not inhibit myosin loss in the EDL muscle. In the liver, dexamethasone induced changes in several proteins related to glucose metabolism; however, HMB was unable to attenuate dexamethasone-induced hepatic glycogenolysis.

**Supplementary Information:**

The online version contains supplementary material available at 10.1007/s12013-026-01995-4.

## Introduction

Skeletal muscle tissue is a highly dynamic and adaptable structure, capable of adjusting its phenotype and function in response to various stimuli, including neural activation, mechanical load, hormonal factors, cytokines, and nutritional status [1, 2]. These adaptations lead to both qualitative and quantitative changes in muscle fibers, primarily characterized by alterations in their cross-sectional area, which directly affect muscle strength and energy metabolism [1]. The regulation of muscle mass driven by these stimuli is essential for maintaining mobility, functionality, and quality of life, and serves as an important marker of musculoskeletal health. However, several pathological and physiological conditions, such as aging, immobilization, chronic diseases, metabolic stress, and exposure to catabolic agents like glucocorticoids, promote muscle atrophy, defined by the loss of muscle mass and strength due to an imbalance between protein synthesis and degradation, with a predominance of catabolism [3–5]. This condition poses a major clinical challenge, as it is linked to decreased functional independence, increased morbidity and mortality, and a significant decline in quality of life [6]. Despite therapeutic approaches that include nutritional supplementation, physical exercise, and hormonal interventions, there is still no fully effective treatment for muscle atrophy, underscoring the need to deepen our understanding of the involved molecular mechanisms [7, 8].

Glucocorticoids are steroid hormones essential for metabolic homeostasis, especially in response to stress, playing a crucial role in regulating the intermediary metabolism of carbohydrates, lipids, and proteins [9, 10]. However, long-term exposure or high-dose therapeutic use such as with dexamethasone, is directly linked to the development of muscle atrophy [11, 12]. These hormones inhibit muscle protein synthesis and activate degradative pathways, such as the ubiquitin-proteasome system (UPS), mainly by increasing the expression of muscle-specific E3 ligases, Atrogin-1 and MuRF-1, which target contractile proteins for degradation [4, 13]. In addition, glucocorticoids influence the nutrient-regulated autophagy pathway, which also contributes to maintaining muscle protein balance but, when excessive, promotes muscle loss [14, 15].

Dexamethasone also affects various processes by acting on metabolic tissues, including the liver, by modulating gluconeogenesis and hepatic mitochondrial bioenergetics, thereby impacting whole-body metabolism [16, 17]. Its high affinity for hepatic glucocorticoid receptors contributes to its preferential accumulation in this organ, leading to metabolic alterations that may adversely affect nutritional status and systemic energy homeostasis [10, 18].

Given the deleterious effects of glucocorticoids, the search for therapeutic alternatives that can minimize muscle loss, and its complications has gained increasing attention. β-Hydroxy-β-Methylbutyrate (HMB), a leucine metabolite, has emerged as a promising candidate due to its proven anticatabolic effects in both preclinical and clinical studies, in sports contexts as well as in clinical conditions associated with accelerated muscle mass loss, such as cancer cachexia, sepsis, and muscle disuse [11, 12, 19]. HMB stimulates anabolic pathways, particularly the mTOR pathway, enhancing protein synthesis, while also inhibiting muscle degradative systems, thereby contributing to preserving muscle mass [20, 21]. Furthermore, HMB promotes the recruitment and proliferation of muscle satellite cells, facilitating regeneration and recovery during atrophic conditions [22].

Although previous studies have investigated the effectiveness of HMB in protecting against dexamethasone-induced muscle atrophy in some muscle types, significant gaps remain concerning its effects at higher glucocorticoid doses and across different muscle profiles, including predominantly glycolytic (white) muscles, as well as its potential synergistic or modulatory effects on the liver, a key organ in energy metabolism [4, 11, 12, 17, 23]. A detailed understanding of the protein alterations induced by these treatments is essential for developing more effective strategies to combat muscle atrophy and its systemic consequences. Thus, this study aims to evaluate the proteomic profile across various muscles and the liver of rats subjected to severe model of dexamethasone-induced muscle atrophy, also investigating the potential protective effects of HMB in this condition.

## Materials and Methods

### Ethical Aspects and Experimental Design

This research was conducted with the approval of the Ethics Committee in Education and Research on Animals (CEEPA) under protocol #018/2013, at the Bauru Dental School, University of São Paulo, and the guidelines of the Animals in Research: Reporting In Vivo Experiments (ARRIVE).

Twenty-four male Wistar rats, 60 days old, were used, supplied by the Animal Facility of the Bauru School of Dentistry at the University of São Paulo. The animals were housed in a clean, pathogen-free environment under a 12-hour light/dark cycle at 22 °C. A maximum of four animals were housed per cage and had free access to water and food *ad libitum*.

Before any intervention, the animals were weighed and assigned to ensure each group had roughly the same average body weight and consisted of animals with similar weights. After this assignment, randomization was used to decide which treatment each group would receive.

This methodology was chosen because of the well-established link between body weight, muscle mass, and the cross-sectional area of various muscle fiber types. The aim was to ensure that no group started with heavier or lighter animals, which could influence the outcomes.

The animals were distributed into the following groups: Placebo Experimental Group (PEG), Dexamethasone Experimental Group (DEG) and Dexamethasone + HMB Experimental Group (DEHG), consisting of *n* = 8 for each group. After group assignment, all animals received daily treatments for 10 consecutive days according to their respective protocols.

### Drug Administration Protocol

For the groups treated with dexamethasone, the protocol described by Barel et al. (2010) was followed, using a dose of 1 mg/kg of dexamethasone (Decadron^®^) diluted in saline solution and administered intraperitoneally for 10 consecutive days. Groups that did not receive dexamethasone were also given intraperitoneal injections of saline solution only, in the same volume as the treated groups.

Regarding the group treated with β-hydroxy-β-MethylButyrate (HMB), HMB was administered via gavage. It was given at a dose of 0.3 g/kg of body weight, diluted in 1 mL of physiological solution, daily, for 10 consecutive days. For the groups that did not receive HMB, gavage was performed with saline solution only.

### Group Treatments

In the Placebo Experimental Group (PEG), animals were treated for 10 consecutive days with both oral gavage and intraperitoneal injections containing only saline solution. This protocol ensured that this group experienced treatment-related stress, without exposure to any pharmacological agents. In the Dexamethasone Experimental Group (DEG), animals received oral gavage with saline solution and intraperitoneal injections of dexamethasone for 10 consecutive days, thereby experiencing both dexamethasone effects and the same gavage-induced stress. Finally, in the Dexamethasone + HMB Experimental Group (DEHG), animals were treated for 10 consecutive days with oral gavage containing HMB and intraperitoneal injections of dexamethasone, allowing assessment of whether HMB could attenuate or prevent dexamethasone-induced muscle atrophy.

### Sample Collection

After 10 days of treatment, the animals were euthanized with anesthetic overdose of Xylazine Hydrochloride, Anasedan^®^, Vetbrands, São Paulo, Brazil), combined with Ketamine Hydrochloride (50 mg/kg – Dopalen^®^, Vetbrands, São Paulo, Brazil) and the Soleus, Extensor Digitorum Longus (EDL) muscles, and liver were collected and immediately frozen by immersion in liquid nitrogen and stored at -80 °C (INDREL^®^, IULT 2430, Londrina, Paraná, Brazil).

### Proteomic Analysis

After removal from the freezer, samples were kept submerged in liquid nitrogen to ensure they remained frozen during handling. Tissue homogenization was performed using a cryogenic mill, which allows pulverization of samples at low temperatures, transforming them into frozen powder and thus preventing protein degradation during handling.

For homogenization, 100 mg of tissue was mixed with 500 µL of lysis buffer (Urea/Thiourea) and incubated for 1 h at 4 °C with occasional agitation. Samples were centrifuged at 14.000 rpm for 30 min at 4 °C, and the supernatant was collected. Desalting was performed using Amicon^®^ Ultra Millipore 3 KDa columns (UFC8003). Protein quantification was carried out using the Bradford method with the Quick Start™ Bradford Protein Assay kit (Bio-Rad, Hercules, CA, USA), in triplicate. Peptide analysis was performed using a Xevo G2 mass spectrometer (Waters Technologies) coupled to the nanoACQUITY system (Waters, Manchester, UK). The Nano-UPLC (Nano-Ultra Performance Liquid Chromatography, Waters^®^, Barueri, SP, Brazil) was used for high-performance protein separation prior to mass spectrometry. Peptide identification was performed using the nanoACQUITY UPLC-Xevo QTof MS system.

Chromatographic separation was performed using a nanoACQUITY HSS T3 reverse-phase analytical column (75 μm × 150 mm, 1.8 μm particle size, Waters, UK). The column was equilibrated with 93% mobile phase A (0.1% formic acid in water) and 7% mobile phase B (100% ACN + 0.1% formic acid). Peptides were separated with a linear gradient from 7to 85% mobile phase B over 70 min at a flow rate of 0.35 µL/min, with the column maintained at 35 °C.

Data were acquired using the MSE elevated-energy method (19–5 V) on the Xevo^®^ G2 Q-TOF operated in positive-ion nano-electrospray mode, enabling the acquisition of both precursor and fragment ions in a single injection. Source conditions included capillary voltage 2.5 kV, sample cone 30 V, extraction cone 5 V, and source temperature 80 °C. Data were collected for 70 min, scanning a mass range of 50–2000 Da. A lockspray with [Glu]-fibrinopeptide (1 pmol/µL, flow 1 µL/min, m/z 785.8427) was used for accuracy and reproducibility. Protein identification was performed using ProteinLynx Global Server (PLGS) v3.0 *software*, based on the built-in ion counting algorithm.

Protein identities were matched against the Rattus norvegicus database (UniProtKB/Swiss-Prot). Expression differences between groups were determined using *PLGS software*, considering *p* < 0.05 for downregulated proteins and 1-*p* > 0.95 for upregulated proteins.

### Bioinformatics Analysis

Protein profiles from each sample were analyzed using CYTOSCAPE^®^ v3.7.0 (Java^®^ 1.8.0_162). All proteins identified by mass spectrometry were uploaded into the *software* using their accession IDs from the UniProt database (www.uniprot.org). Proteins were initially confirmed in UniProt, and a primary network was generated. Taxonomy was restricted to *Rattus norvegicus* (10116). Proteins with a ratio > 1 were classified as upregulated, and those with a ratio < 1 as downregulated. Additional subclassifications were selected to build more specific networks, with Cytoscape^®^ also suggesting related proteins from the same taxonomy that were not experimentally detected but are known to interact with those identified. Literature searches for the identified proteins were conducted using PubMed (https://www.ncbi.nlm.nih.gov/pmc). Using the ClueGO^®^ plug-in in Cytoscape^®^, proteins were classified according to their biological processes. This enabled the integration of genes, proteins, and mRNAs into subnetworks generated by ClueGO^®^, facilitating the identification of interrelationships and potential new associations. Therefore, the Biological Process category was used. Significant results were considered when the Kappa score was > 0.4, based on the percentage of associated genes provided by Cytoscape^®^.

### Histological Processing

After weighing, muscle specimens of the right hindlimb were wrapped in Tissue-Tek^®^ (O.C.T., Sakura Finetek, Torrance, USA) and immediately immersed in liquid nitrogen for histological processing. Sections with a thickness of 10 μm were obtained in a cryostat at -20 °C and placed on histological slides. The cross-sectional area and minimum fiber diameter were measured on slides stained with Hematoxylin and Eosin (HE), and Masson’s trichrome staining was used to measure the percentage of connective tissue in the muscle.

The images were captured with an Olympus BX-50 photomicroscope, coupled to a computer, and three fields from each sample were photographed with a 20x objective. For morphometric analysis 220 muscle fibers of each animal were manually measured, area and the Minimum diameter, through Image Pro-Plus^®^ 6.2 program (Media Cybernetics, Bethesda, MD, USA).

#### Density of Connective Tissue Measurements

Using Masson’s trichrome staining, a photo of the entire muscle cross-section was captured with a 2x objective to measure its total area, and then 20x objective photos were captured in Image Pro-Plus^®^ 6.2 to identify Aniline Blue (responsible for marking connective tissue in this technique) with greater fidelity. After the measurements, the percentage of connective tissue in the muscle was calculated.

#### Statistical Analysis of Cross-sectional area, Minimum Diameter and Connective Tissue

The morphometric values (cross-sectional area, minimum diameter, and percentage of connective tissue) were analyzed using One-Way ANOVA followed by the Tukey test. The value of *p* < 0.05 was considered statistically significant.

## Results

### Soleus Muscle Proteomic Analysis

In the proteomic analysis of the soleus muscle, a total of 654, 700, and 689 protein records were identified in each group, corresponding to PEG, DEG, and DEHG, respectively. Subsequently, in the qualitative analysis of the proteins, only those identified as unique to each group and those with altered expression (interactions) remained. A complete description of these proteins is provided in the tables included in the supplementary materials.

For the soleus muscle, among the unique proteins, 154 remained in the PEG group (Table S1), 179 in the DEG group (Table S2), and 172 in the DEHG group (Table S3).

In the comparison between DEG vs. PEG, among the significantly altered proteins (either downregulated or upregulated), a total of 23 proteins remained, with 22 proteins downregulated and 1 protein upregulated, as shown in Table S4.

**Fig. 1:**
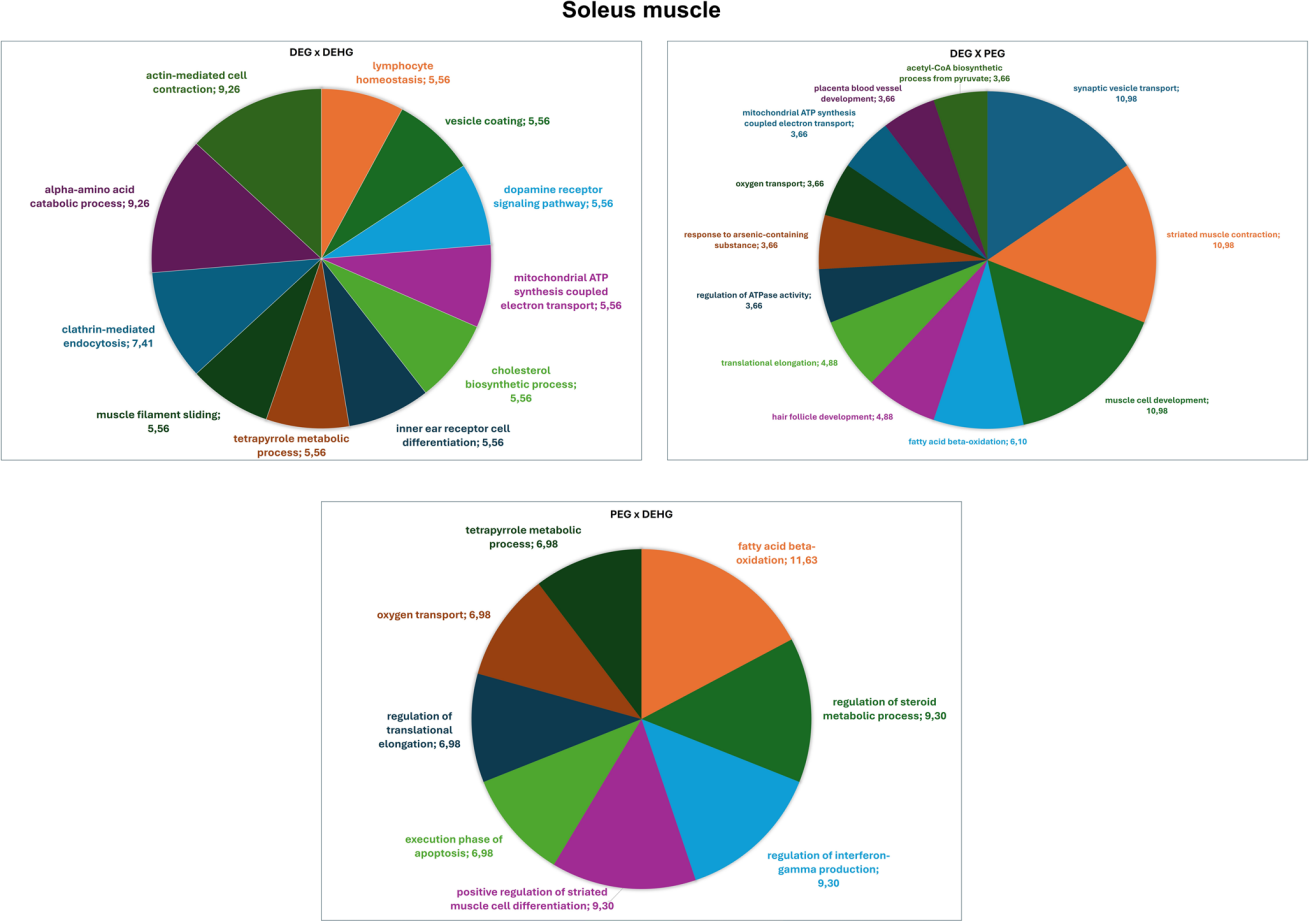
Distribution of uniquely identified proteins and those with differential expression in the Soleus muscle in the comparisons DEG vs. PEG, DEHG vs. PEG, and DEHG vs. DEG. Categories are shown based on gene ontology for Biological Processes, generated with Cytoscape 3.0.2^®^. Only significant terms were considered (k = 0.04), and the distribution is shown according to the number of genes associated with each term. Protein accession numbers were obtained from UniProt^®^. Gene ontology was assessed using the ClueGo^®^ plugin in Cytoscape. Only categories representing more than 3% are displayed in the graph

The functional analysis of biological processes based on Gene Ontologies (GO) for the most significant term, comparing DEG vs. PEG, is presented (Fig. 1). Among them, the categories with the highest percentage of genes include *synaptic vesicle transport* (10.98%), *striated muscle contraction* (10.98%), *muscle cell development* (10.98%), *fatty acid beta-oxidation* (6.10%), *hair follicle development* (4.88%), *translational elongation* (4.88%), *regulation of ATPase activity* (3.66%), *response to arsenic-containing substance* (3,66%), *oxygen transport* (3.66%), *mitochondrial ATP synthesis coupled electron transport* (3.66%), *placenta blood vessel development* (3.66%), and *acetyl-CoA biosynthetic process from pyruvate* (3,66%).

**Fig. 2:**
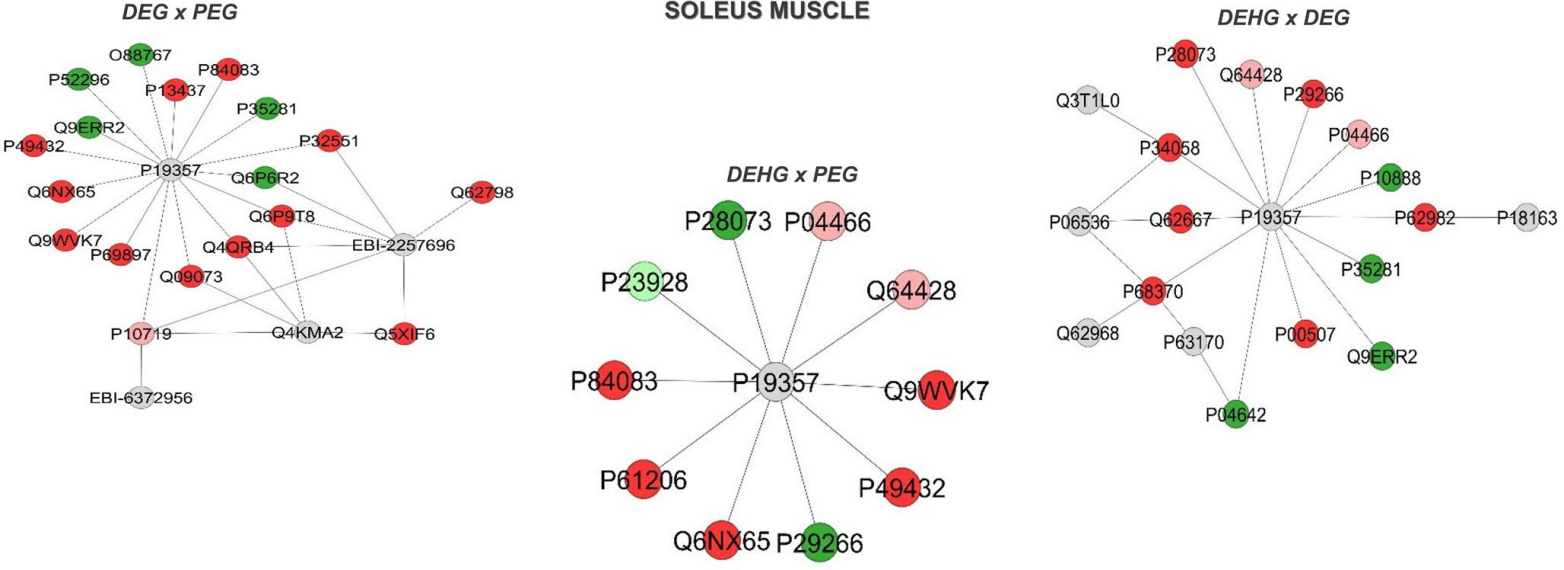
Subnetworks generated by JActiveModules to show interactions among the proteins identified in the Soleus muscle with differential expression in the comparisons: DEG vs. PEG, DEHG vs. PEG, and DEHG vs. DEG. The node colors represent the differential expression of each protein, labeled with its accession code. Dark red indicates unique proteins, while light red indicates downregulated proteins. Dark green indicates proteins that are unique, while light green signifies upregulated proteins. Gray nodes and their accession codes represent interaction proteins provided by Cytoscape^®^, which were not identified in this study. This color scheme was consistent across all networks

In the protein interactions in DEG vs. PEG the accession numbers in the dark green nodes correspond to: *COMM domain-containing protein 5* (Q9ERR2), *Importin subunit beta-1* (P52296), *Parkinson’s disease protein 7 homolog* (O88767), *Ras-related protein Rab-10* (P35281), and *Dihydrolipoyl dehydrogenase*,* mitochondrial* (Q6P6R2). The accession numbers in the dark red nodes correspond to: *3-ketoacyl-CoA thiolase*,* mitochondrial* (P13437), *ADP-ribosylation factor 5* (P84083), *Cytochrome b-c1 complex subunit 2*,* mitochondrial* (P32551), *Dorsal root ganglia homeobox protein* (Q62798), *Tubulin alpha-4 A chain* (Q5XIF6), *Tubulin beta-4B chain* (Q6P9T8), *Tubulin beta-3 chain* (Q4QRB4), *ADP/ATP translocase 2* (Q09073), *Tubulin beta-5 chain* (P69897), *Hydroxyacyl-coenzyme A dehydrogenase*,* mitochondrial* (Q9WVK7), *Programmed cell death protein 10* (Q6NX65), and *Pyruvate dehydrogenase E1 component subunit beta*,* mitochondrial* (P49432). The accession numbers in the light red nodes correspond to: *ATP synthase subunit beta*,* mitochondrial* (P10719). The accession numbers in the gray nodes correspond to: *Solute carrier family 2*,* facilitated glucose transporter member 4* (P19357), *UV excision repair protein RAD23 homolog B* (Q4KMA2), *Uncharacterized protein* (EBI-2257696), and *Uncharacterized protein* (EBI-6372956) (Fig. 2).

In the comparison PEG vs. DEHG, besides the unique proteins of each group, a total of 21 proteins with statistically significant differential expression were identified, of which 3 were downregulated and 18 were upregulated, as shown in Table S5.

The functional analysis according to the biological process by Gene Ontologies (GO) with the most significant term for the comparison between PEG vs. DEHG is presented (Fig. 1). Among them, the categories with the percentage of genes include fatty acid beta-oxidation (11.63%), regulation of steroid metabolic process (9.30%), regulation of interferon-gamma production (9.30%), positive regulation of striated muscle cell differentiation (9.30%), execution phase of apoptosis (6.98%), regulation of translational elongation (6.98%), oxygen transport (6.98%), and tetrapyrrole metabolic process (6.98%).

Regarding the proteins interaction in PEG vs. DEHG comparison, the accession numbers in the dark green nodes correspond to: *Major vault protein* (Q62667), *Cullin-3* (B5DF89), *Tumor protein p53-binding protein 2* (F1M5H6), Inter-alpha-trypsin inhibitor heavy chain 2 (D3ZFH5), and *Very long-chain acyl-CoA synthetase* (P97524). The accession numbers in the light green nodes correspond to: *Hemoglobin subunit alpha-1/2* (P01946), *Hemoglobin subunit beta-1* (P02091), and *Albumin* (P02770). The accession numbers in the dark red nodes correspond to: *Nucleobindin-1* (Q63083). The accession numbers in the gray nodes correspond to: *Glucocorticoid receptor* (P06536), *Aldehyde dehydrogenase family 16 member A1* (Q3T1L0), and *Insulin receptor* (P15127) (Fig. 2).

Finally, in the last comparison between DEG vs. DEHG, a total of 22 proteins showed statistically significant differential expression with 19 proteins downregulated, and 3 proteins upregulated, as shown in Table S6.

The functional analysis based on the biological process by Gene Ontologies (GO), for the comparison between DEG vs. DEHG, highlighting the most significant term is presented (Fig. 1). Among them, the following categories with the percentage of genes include alpha-amino acid catabolic process (9.26%), actin-mediated cell contraction (9.26%), clathrin-mediated endocytosis (7.41%), lymphocyte homeostasis (5.56%), vesicle coating (5.56%), dopamine receptor signaling pathway (5.56%), mitochondrial ATP synthesis coupled electron transport (5.56%), cholesterol biosynthetic process (5.56%), inner ear receptor cell differentiation (5.56%), tetrapyrrole metabolic process (5.56%), and muscle filament sliding (5.56%).

In the protein interaction in DEG vs. DEHG, the accession numbers in the dark green nodes represent: *Cytochrome c oxidase subunit 4 isoform 1*,* mitochondrial* (P10888), *Ras-related protein Rab-10* (P35281), *COMM domain-containing protein 5* (Q9ERR2), and *L-lactate dehydrogenase A chain* (P04642). The accession numbers in the light red nodes correspond to: *Trifunctional enzyme subunit alpha*,* mitochondrial* (Q64428) and *Myosin regulatory light chain 2*,* skeletal muscle isoform* (P04466). The accession numbers in the dark red nodes correspond to: *3-hydroxyisobutyrate dehydrogenase*,* mitochondrial* (P29266), *Ubiquitin-40 S ribosomal protein S27a* (P62982), *Aspartate aminotransferase*,* mitochondrial* (P00507), *Tubulin alpha-1 A chain* (P68370), *Major vault protein* (Q62667), *Heat shock protein HSP 90-beta* (P34058), and *Proteasome subunit beta type-6* (P28073). The accession numbers in the gray nodes correspond to: *Solute carrier family 2*,* facilitated glucose transporter member 4* (P19357), *Long-chain-fatty-acid–CoA ligase 1* (P18163), *Dynein light chain 1*,* cytoplasmic* (P63170), *Sodium channel protein type 10 subunit alpha* (Q62968), *Glucocorticoid receptor* (P06536), and *Aldehyde dehydrogenase family 16 member A1* (Q3T1L0) (Fig. 2).

### EDL Muscle Proteomic Analysis

For the EDL muscle, a total of 392, 396, and 562 proteins were identified in the PEG, DEG, and DEHG groups, respectively. Regarding the exclusive proteins, 107 Table S7), 119 (Table S8), and 174 (Table S9) were observed in PEG, DEG, and DEHG, respectively.

Twenty-seven interaction proteins with significant differential expression were observed in the DEG vs. PEG comparison, which 14 proteins showed decreased expression (downregulated) and 13 proteins showed increased expression (up-regulated), as shown in Table S10.

**Fig. 3:**
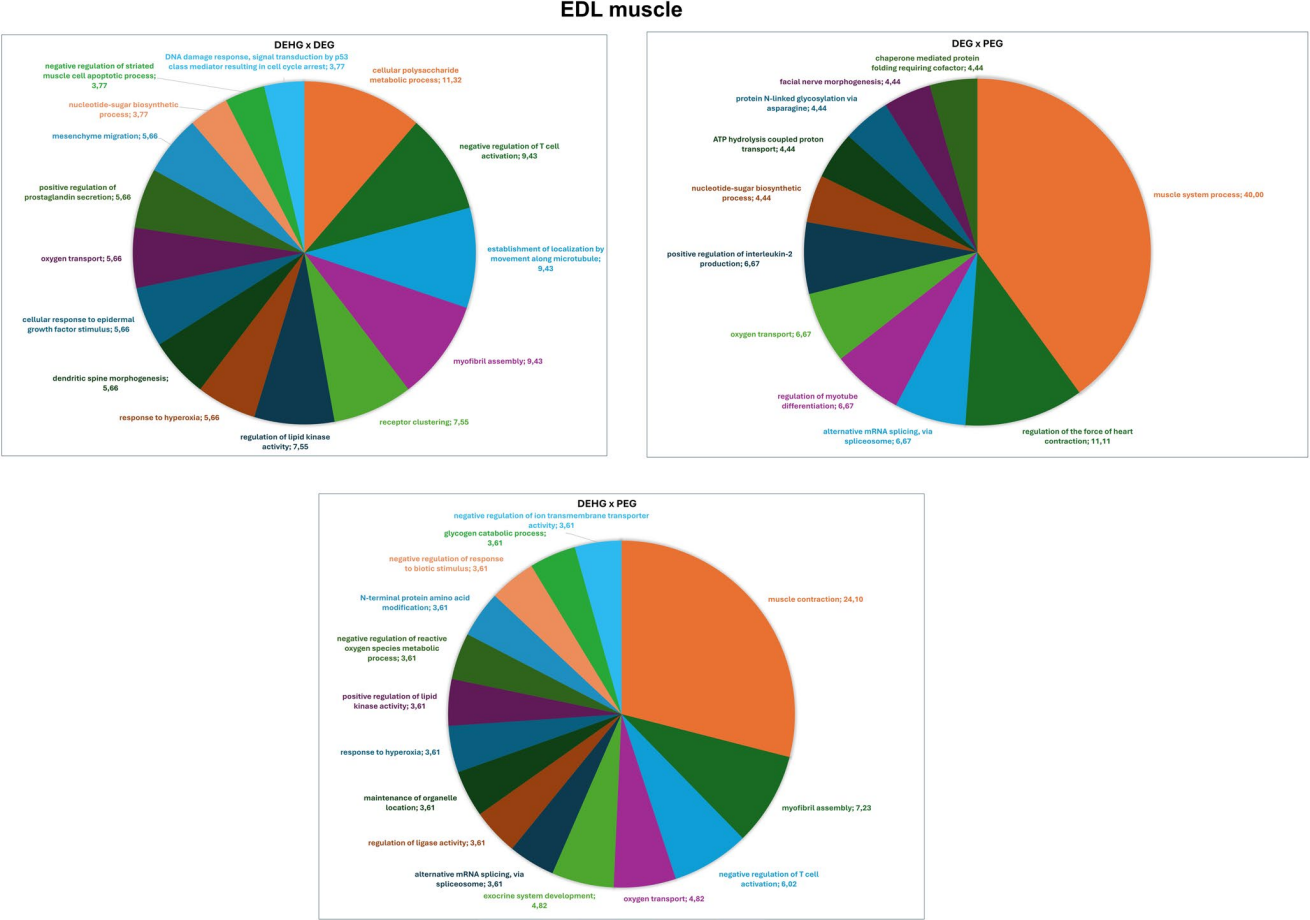
Distribution of uniquely identified proteins and those with differential expression in the EDL muscle in the comparisons -DEG vs. PEG, DEHG vs. PEG, and DEHG vs. DEG. Categories are shown based on gene ontology for Biological Processes, generated with Cytoscape 3.0.2^®^. Only significant terms were considered (k = 0.04), and the distribution is shown according to the number of genes associated with each term. Protein accession numbers were retrieved from UniProt^®^. Gene ontology was assessed using the ClueGo^®^ plugin in Cytoscape. Only categories representing more than 3% are displayed in the graph

The functional analysis according to the biological process by Gene Ontologies (GO), highlighting the most significant term, for the comparison between DEGvs. PEG, is presented (Fig. 3). Among them, the categories with the percentages of genes include muscle system process (40.00%), regulation of the force of heart contraction (11.11%), alternative mRNA splicing, via spliceosome (6.67%), regulation of myotube differentiation (6.67%), oxygen transport (6.67%), positive regulation of interleukin-2 production (6.67%), nucleotide-sugar biosynthetic process (4.44%), ATP hydrolysis coupled proton transport (4.44%), protein N-linked glycosylation via asparagine (4.44%), facial nerve morphogenesis (4.44%), and chaperone mediated protein folding requiring cofactor (4.44%).

**Fig. 4:**
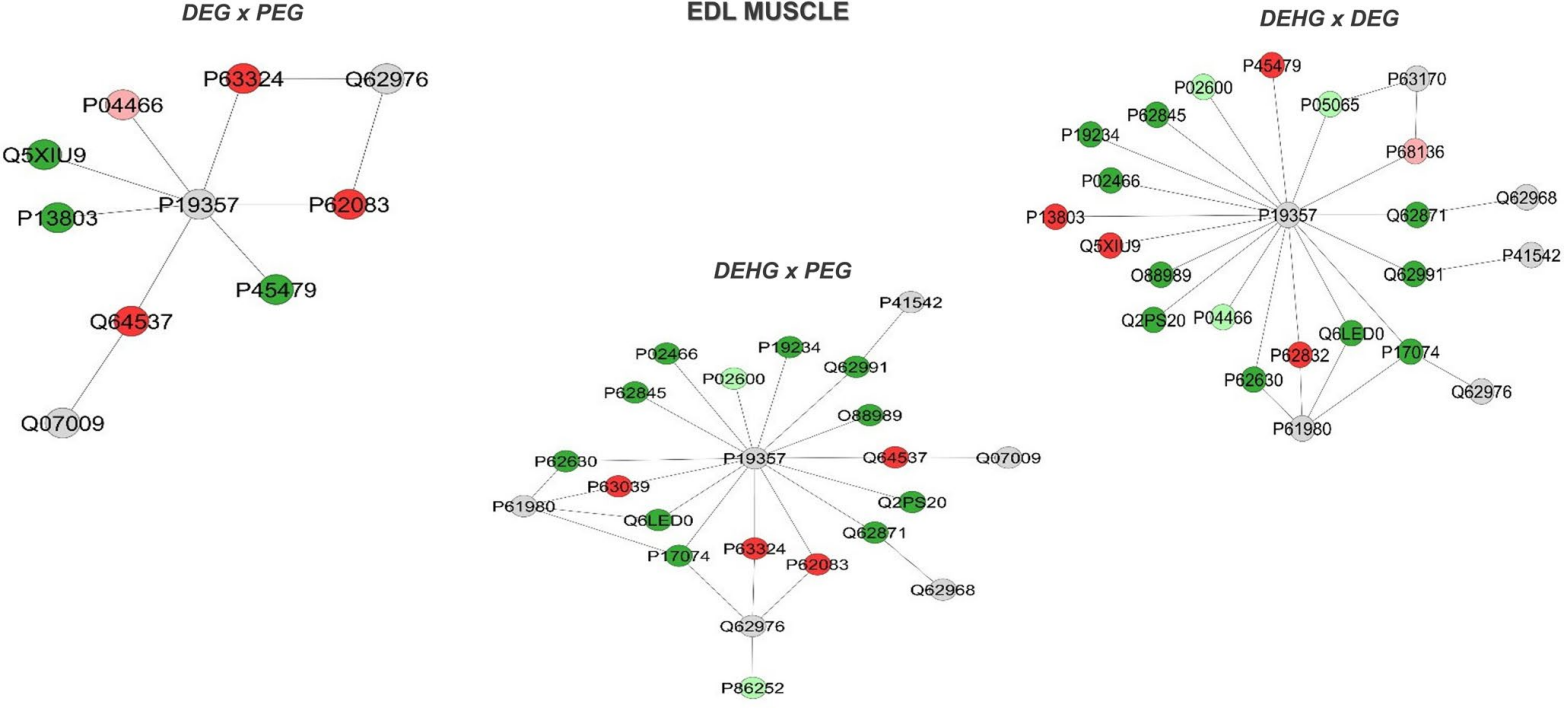
Subnetworks generated by JActiveModules showing interactions between proteins identified in the EDL muscle with differential expressions in the comparison: DEG vs. PEG, DEHG vs. PEG, and DEHG vs. DEG. The node colors indicate the differential expression of the respective protein, labeled with its accession code. Dark red indicates unique proteins, while light red indicates downregulated proteins. Dark green indicates proteins uniquely, while light green identifies upregulated proteins. Gray nodes and their accession codes represent interaction proteins provided by Cytoscape^®^, which were not identified in the present study. This color scheme was maintained in all networks

In the protein interactions in DEG vs. PEG, the accession numbers in the dark green nodes correspond to: *Membrane-associated progesterone receptor component 2* (Q5XIU9), *Electron transfer flavoprotein subunit alpha*,* mitochondrial* (P13803), and *Palmitoyl-protein thioesterase 1* (P45479). The accession numbers in the dark red nodes correspond to: *40 S ribosomal protein* S12 (P63324), *40 S ribosomal protein S7* (P62083), and *Calpain small subunit 1* (Q64537). The accession numbers in the light red nodes correspond to: *Myosin regulatory light chain 2*,* skeletal muscle isoform* (P04466). The accession numbers in the gray nodes correspond to: *Solute carrier family 2*,* facilitated glucose transporter member 4* (P19357), *Calcium-activated potassium channel subunit alpha-1* (Q62976), and *Calpain-2 catalytic subunit* (Q07009) (Fig. 4).

Thirty-eight proteins with significant differential expression were identified in the comparison DEHG vs. PEG, including 11 downregulated and 27 upregulated as shown in Table S11.

The functional analysis according to the biological process by Gene Ontologies (GO), highlighting the most significant term, for the comparison between DEHG vs. PEG is presented (Fig. 3). Among them, the categories with the percentages of genes include muscle contraction (24.10%), myofibril assembly (7.23%), negative regulation of T cell activation (6.02%), oxygen transport (4.82%), exocrine system development (4.82%), alternative mRNA splicing, via spliceosome (3.61%), regulation of ligase activity (3.61%), maintenance of organelle location (3.61%), response to hyperoxia (3.61%), positive regulation of lipid kinase activity (3.61%), negative regulation of reactive oxygen species metabolic process (3.61%), N-terminal protein amino acid modification (3.61%), negative regulation of response to biotic stimulus (3.61%), glycogen catabolic process (3.61%), and negative regulation of ion transmembrane transporter activity (3.61%).

Regarding the protein interaction (Fig. 4), the accession numbers in the dark red nodes correspond to: 60 kDa heat shock protein, mitochondrial (P63039), *40 S ribosomal protein S12* (P63324), *40 S ribosomal protein S7* (P62083), *Calpain small subunit 1* (Q64537). The accession numbers in the dark green nodes correspond to: *NADH dehydrogenase [ubiquinone] flavoprotein 2*,* mitochondrial* (P19234), *Sect. 1 family domain-containing protein 1* (Q62991), *Malate dehydrogenase*,* cytoplasmic* (O88989), *Junctophilin-2* (Q2PS20), *Cytoplasmic dynein 1 intermediate chain 2* (Q62871), *40 S ribosomal protein S19* (P17074), *Histone H3.1* (Q6LED0), *Elongation factor 1-alpha 1* (P62630), *40 S ribosomal protein S15* (P62845), *Collagen alpha-2(I) chain* (P02466). The accession numbers in the light green nodes correspond to: *Myosin light chain 1/3*,* skeletal muscle isoform* (P02600), *Transcriptional activator protein Pur-alpha* (P86252). The accession numbers in the gray nodes correspond to: *Solute carrier family 2*,* facilitated glucose transporter member 4* (P19357), *General vesicular transport factor p115* (P41542), *Calpain-2 catalytic subunit* (Q07009), *Sodium channel protein type 10 subunit alpha* (Q62968), *Calcium-activated potassium channel subunit alpha-1* (Q62976), *Heterogeneous nuclear ribonucleoprotein K* (P61980).

Twenty-seven proteins with significant differential expression were observed when comparing the two groups that received dexamethasone, DEHG vs. DEG, including eight downregulated and nineteen upregulated proteins, as shown in Table S12.

The functional analysis according to the biological process by Gene Ontologies (GO) with the most significant term for the comparison between DEHG vs. DG is presented (Fig. 3). Among them, the categories with the percentages of genes include cellular polysaccharide metabolic process (11.32%), negative regulation of T cell activation (9.43%), establishment of localization by movement along microtubule (9.43%), myofibril assembly (9.43%), receptor clustering (7.55%), regulation of lipid kinase activity (7.55%), response to hyperoxia (5.66%), dendritic spine morphogenesis (5.66%), cellular response to epidermal growth factor stimulus (5.66%), oxygen transport (5.66%), positive regulation of prostaglandin secretion (5.66%), mesenchyme migration (5.66%), nucleotide-sugar biosynthetic process (3.77%), negative regulation of striated muscle cell apoptotic process (3.77%), DNA damage response signal transduction by p53 class mediator resulting in cell cycle arrest (3.77%).

Additionally, regarding protein interactions between DEHG vs. DEG, the accession numbers in the dark green nodes correspond to: *NADH dehydrogenase [ubiquinone] flavoprotein 2*,* mitochondrial* (P19234), *Sect. 1 family domain-containing protein 1* (Q62991), *Malate dehydrogenase*,* cytoplasmic* (O88989), *Junctophilin-2* (Q2PS20), *Cytoplasmic dynein 1 intermediate* chain 2 (Q62871), *40 S ribosomal protein S19* (P17074), *Histone H3.1* (Q6LED0), *Elongation factor 1-alpha 1* (P62630), *40 S ribosomal protein S15* (P62845), *Collagen alpha-2(I) chain* (P02466). The accession numbers in the light green nodes correspond to: *Myosin light chain 1/3*,* skeletal muscle isoform* (P02600), *Fructose-bisphosphate aldolase A* (P05065), *Myosin regulatory light chain 2*,* skeletal muscle isoform* (P04466). The accession numbers in the dark red nodes correspond to: *Palmitoyl-protein thioesterase 1* (P45479), *60 S ribosomal protein L23* (P62832), *Electron transfer flavoprotein subunit alpha*,* mitochondrial* (P13803), *Membrane-associated progesterone receptor component 2* (Q5XIU9). The accession number in the light red node corresponds to: *Actin*,* alpha skeletal muscle* (P68136). The accession numbers in the gray nodes correspond to: *Solute carrier family 2*,* facilitated glucose transporter member 4* (P19357), *Dynein light chain 1*,* cytoplasmic* (P63170), *Sodium channel protein type 10 subunit alpha* (Q62968), *General vesicular transport factor p115* (P41542), *Calcium-activated potassium channel subunit alpha-1* (Q62976), *Heterogeneous nuclear ribonucleoprotein K* (P61980) (Fig. 4).

### Liver Proteomic Analysis

A total of 1.069, 687, and 523 proteins were observed in the PEG, DEG, and DEHG groups, respectively. A qualitative analysis of the liver proteins also revealed exclusive proteins for each group, including 309 in the PEG (Table S13), 206 in the DEG (Table S14), and 129 in the DEHG (Table S15).

In the DEG vs. PEG comparison, in addition to the unique proteins of each group, proteins present in both groups that exhibited altered expression were also included. Among the proteins with significantly altered expression (downregulated or upregulated), 112 showed statistically significant differential expression, with 69 downregulated and 43 upregulated (Table S16).

**Fig. 5:**
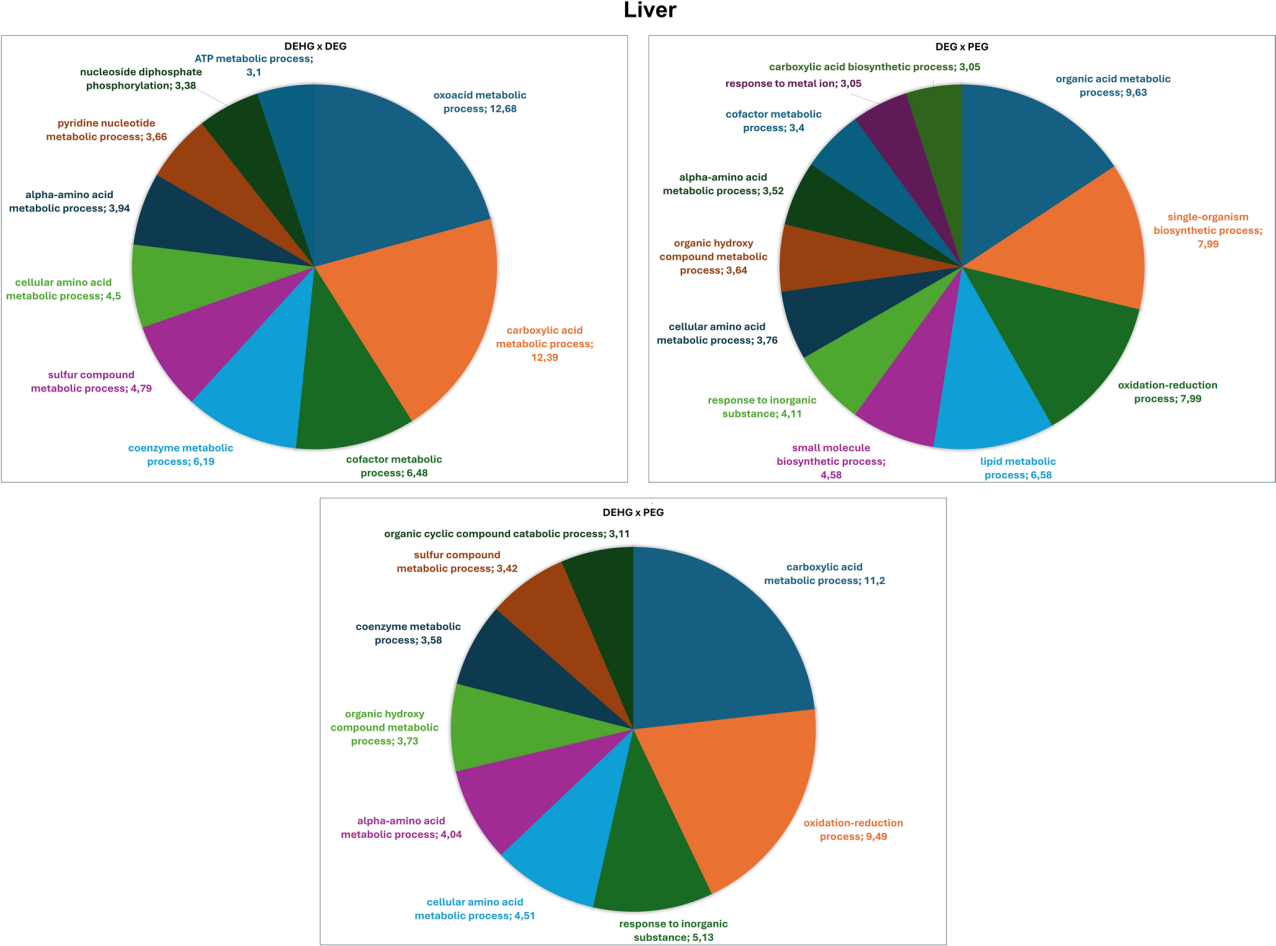
Distribution of uniquely identified proteins and those with differential expressions in the Liver in the comparisons DEG vs. PEG, DEHG vs. PEG, and DEHG vs. DEG. Categories are presented based on gene ontology for Biological Processes, generated with Cytoscape 3.0.2^®^. Only significant terms were considered (k = 0.04), and the distribution is shown according to the number of genes associated with each term. Protein accession numbers were retrieved from UniProt^®^. Gene ontology was assessed using the ClueGo^®^ plugin in Cytoscape. Only categories representing more than 3% are displayed in the graph

The functional analysis according to the biological process by Gene Ontologies (GO) with the most significant term for the comparison between DEG vs. PEG is presented (Fig. 5). Among them, the categories with the percentages of genes include organic acid metabolic process (9.63%), oxidation-reduction process (7.99%), single-organism biosynthetic process (7.99%), lipid metabolic process (6.58%), small molecule biosynthetic process (4.58%), response to inorganic substance (4.11%), cellular amino acid metabolic process (3.76%), organic hydroxy compound metabolic process (3.64%), alpha-amino acid metabolic process (3.52%), cofactor metabolic process (3.41%), response to metal ion (3.06%), and carboxylic acid biosynthetic process (3.06%) (Fig. 3).

**Fig. 6:**
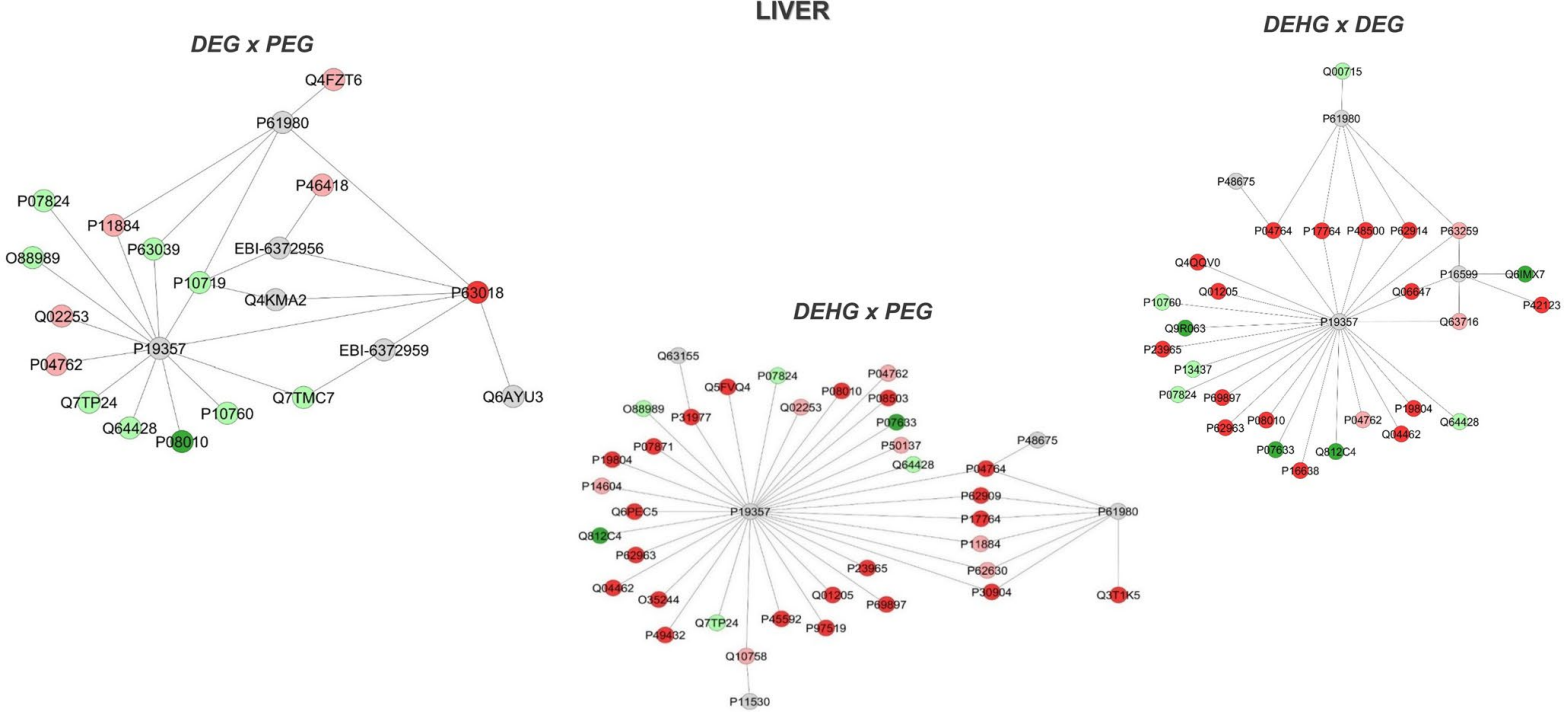
Subnetworks generated by JActiveModules to establish interaction between the proteins identified in the Liver with differential expressions in the comparison: DEG vs. PEG, DEHG vs. PEG and DEHG vs. DEG. The node colors indicate the differential expression of the respective protein, labeled with its accession code. Dark red indicates unique proteins, while light red indicates downregulated proteins. Dark green indicates proteins that are unique, while light green identifies upregulated proteins. Gray nodes and their accession codes represent interaction proteins from Cytoscape^®^ that were not identified in the present study. This color scheme was maintained in all networks

Subnetworks also were generated to analyze the interactions of the proteins in DEG vs. PEG comparison. The accession number on the dark green node corresponds to Glutathione S-transferase Mu 2 (P08010) (Fig. 6). The accession numbers on the light green nodes correspond to *Signal recognition particle receptor subunit beta* (Q7TMC7), *Adenosyl homocysteinase* (P10760), *Trifunctional enzyme subunit alpha*,* mitochondrial* (Q64428*)*,* Signal recognition particle receptor subunit beta* (Q7TP24), *Malate dehydrogenase*,* cytoplasmic* (O88989), *Arginase-1* (P07824), *60 kDa heat shock protein*,* mitochondrial* (P63039), and *ATP synthase subunit beta*,* mitochondrial* (P10719). The accession numbers on the light red nodes correspond to *Histone H2A type 3* (Q4FZT6), *Glutathione S-transferase alpha-5* (P46418), *Aldehyde dehydrogenase*,* mitochondrial* (P11884), *Methylmalonate-semialdehyde dehydrogenase [acylating]*,* mitochondrial* (Q02253), and *Catalase* (P04762). The accession number on the dark red node corresponds to *Heat shock cognate 71 kDa protein* (P63018). The accession numbers on the gray nodes correspond to *Heterogeneous nuclear ribonucleoprotein* K (P61980), UV excision repair protein RAD23 homolog B (Q4KMA2), Solute carrier family 2, facilitated glucose transporter member 4 (P19357), *DnaJ homolog subfamily B member* 6 (Q6AYU3), unidentified protein (EBI-6372956), and unidentified protein (EBI-6372959).

The comparison of DEHG vs. PEG revealed a total of 86 interacting proteins with altered expression, including 47 downregulated and 39 upregulated proteins, as detailed in Table S17. The functional analysis based on the biological process by Gene Ontologies (GO) identified the categories with the percentage of genes include carboxylic acid metabolic process (11.20%), followed by oxidation-reduction process (9.49%), response to inorganic substance (5.13%), cellular amino acid metabolic process (4.51%), alpha-amino acid metabolic process (4.04%), organic hydroxy compound metabolic process (3.73%), coenzyme metabolic process (3.58%), sulfur compound metabolic process (3.42%), and organic cyclic compound catabolic process (3.11%). Each of the nine categoriesis labeled with the name of the biological process it represents, along with the corresponding percentage of genes in that category (Fig. 5).

Regarding protein interactions in the DEHG vs. PEG comparison, Fig. 6 the accession numbers in dark red nodes correspond to *Enoyl-CoA delta isomerase 1*, *mitochondrial* (P23965); *Tubulin beta-5 chain (P69897); Dihydrolipoyllysine-residue succinyltransferase component of 2-oxoglutarate dehydrogenase complex*,* mitochondrial* (Q01205); *Hydroxymethylglutaryl-CoA lyase*,* mitochondrial* (P97519); *Cofilin-1* (P45592); *Pyruvate dehydrogenase E1 component subunit beta*,* mitochondrial* (P49432); *Peroxiredoxin-6* (O35244); *Profilin-1* (P62963); *Sod_Cu domain-containing protein* (Q6PEC5); *Nucleoside diphosphate kinase B* (P19804*); 3-ketoacyl-CoA thiolase B*,* peroxisomal* (P07871); *Ezrin* (P31977); Malectin (Q5FVQ4); *Glutathione S-transferase Mu 2* (P08010); *Medium-chain specific acyl-CoA dehydrogenase*,* mitochondrial* (P08503); *Alpha-enolase* (P04764); *40 S ribosomal protein S3* (P62909); *Acetyl-CoA acetyltransferase*,* mitochondrial (*P17764); *Macrophage migration inhibitory factor* (P30904). The accession numbers in light red nodes correspond to: *Keratin*,* type II cytoskeletal 8* (Q10758); *Enoyl-CoA hydratase*,* mitochondrial* (P14604); *Methylmalonate-semialdehyde dehydrogenase [acylating]*,* mitochondrial* (Q02253); *Catalas*e (P04762); *Transketolase* (P50137); *Aldehyde dehydrogenase*,* mitochondrial* (P11884); *Elongation factor 1-alpha 1* (P62630). The accession numbers in light green nodes correspond to: *Signal recognition particle receptor subunit beta* (Q7TP24); *Malate dehydrogenase*,* cytoplasmic* (O88989); *Arginase-1* (P07824); *Trifunctional enzyme subunit alpha*,* mitochondrial* (Q64428). The accession numbers in dark green nodes correspond to: *Translation initiation factor 4 A*,* isoform 1* (Q812C4); *Propionyl-CoA carboxylase beta chain*,* mitochondrial* (P07633). The accession numbers in gray nodes correspond to: *Dystrophin* (P11530); *Solute carrier family 2*,* facilitated glucose transporter member 4* (P19357); *Netrin receptor DCC* (Q63155); *Desmin* (P48675); *Heterogeneous nuclear ribonucleoprotein K* (P61980) (Fig. 6).

Eighty-three interaction proteins were observed in the DEHG vs. DEG comparison, including 35 downregulated and 48 upregulated proteins (Table S18). Functional analysis based on biological processes include oxoacid metabolic process (12.68%), carboxylic acid metabolic process (12.39%), cofactor metabolic process (6.48%), coenzyme metabolic process (6.19%), sulfur compound metabolic process (4.79%), cellular amino acid metabolic process (4.5%), alpha-amino acid metabolic process (3.94%), and pyridine nucleotide metabolic process (3.66%) (Fig. 5).

Regarding protein interactions in the DEHG vs. DEG comparison, the accession numbers in dark red nodes correspond to Alpha-enolase (P04764), *Acetyl-CoA acetyltransferase*,* mitochondrial* (P17764), *Triosephosphate isomerase* (P48500), *60 S ribosomal protein L11* (P62914), *ATP synthase subunit O*,* mitochondrial* (Q06647), *L-lactate dehydrogenase B chain* (P42123), *Nucleoside diphosphate kinase B* (P19804), *Valine-tRNA ligase* (Q04462), *ATP-citrate synthase* (P16638), *Glutathione S-transferase Mu 2* (P08010), *Profilin-1* (P62963), *Tubulin beta-5 chain* (P69897), *Enoyl-CoA delta isomerase 1*,* mitochondrial* (P23965), *Dihydrolipoyllysine-residue succinyltransferase component of the 2-oxoglutarate dehydrogenase complex*,* mitochondrial* (Q01205), and *Tubulin beta chain* (Q4QQV0). The accession numbers in light red nodes correspond to *Actin*,* cytoplasmic 2* (P63259), *Peroxiredoxin-1* (Q63716), and *Catalase* (P04762). The accession numbers in light green nodes correspond to *Histone H2B type 1* (Q00715), *Trifunctional enzyme subunit alpha*,* mitochondrial* (Q64428), Arginase-1 (P07824), *3-ketoacyl-CoA thiolase*,* mitochondrial* (P13437), and *Adenosylhomocysteinase* (P10760). The accession numbers in dark green nodes correspond to *Hsp70-binding protein 1* (Q6IMX7), *Translation initiation factor 4 A*,* isoform 1* (Q812C4), *Propionyl-CoA carboxylase beta chain*,* mitochondrial* (P07633), and *Peroxiredoxin-5*,* mitochondrial* (Q9R063). The accession numbers in gray nodes correspond to *Heterogeneous nuclear ribonucleoprotein K* (P61980), *Solute carrier family 2*,* facilitated glucose transporter member 4* (P19357), *Tumor necrosis factor* (P16599), and *Desmin* (P48675) (Fig. 6).

The muscle morphology and morphometry showed that in both muscles of GCP animals, there was structural preservation of the polygonal shape of muscle fibers; no cells with membrane rupture were observed in either group for both muscles. However, it is possible to notice that in GCP and GEDH, the nuclei are rounded and arranged at the periphery of the fibers (normal appearance), while in GED, some fibers exhibit flattened nuclei, along with a decrease in fiber area, which is even more evident in the EDL muscle (Supplemental Fig. 1).The morphometry (Table S19) showed that HMB was beneficial only in the soleus muscle, with attenuation of atrophy induced by DEX in the GEDH group. However, in the EDL muscle, GED and GEDH showed similar values, both lower than GCP, indicating that HMB could not prevent DEX-induced atrophy.

Regarding the connective tissue percentage data, only a significant difference was observed between GED and GEDH in the soleus muscle (Supplemental Fig. 2).

## Discussion

As expected, the Dexamethasone protocol used in our study (1 mg/day/kg) successfully induced changes in liver protein expression in the animals and atrophic changes in both muscles. The study by Krug et al. [24] demonstrated, through morphological and morphometric analysis, muscle atrophy in animals subjected to this dexamethasone administration protocol. In our study, we observed molecular pathways and proteins involved in this process, confirming and aligning with previously reported findings [24]. These observations were very clear in our results. When examining the effects of dexamethasone alone, by analyzing the most affected biological processes when comparing dexamethasone to placebo (DEG vs. PEG), we found that in the Soleus muscle, two of the three most impacted categories include “striated muscle contraction” and “muscle cell development,” both with the number of altered genes representing 10.98% of all categories.

It was previously reported that dexamethasone activates various proteolytic pathways in muscle tissue, including the ubiquitin-proteasome system [23] and the autophagy-lysosomal system [4]. Proteins involved in these pathways were also identified in our study, thereby confirming these findings previously described in the literature.

When analyzing the unique proteins of the two groups exposed to the atrophy protocol (DEG and DEHG), several proteins involved in degradation were observed in both muscles. In the Soleus muscle, the DEG group exhibited the presence of Cell death activator CIDE-3, a protein involved in the apoptotic process. The apoptotic role of CIDE proteins was initially demonstrated through overexpression in mammalian cells, and their apoptotic functions were initially considered independent of caspase activation. However, later studies revealed that this function depends on caspase-3, caspase-9, and cytochrome c release [25, 26].

The soleus muscle of the DEG group also showed the NEDD8-activating enzyme E1 regulatory subunit. In the NEDD8-mediated ubiquitination system, the NEDD8-activating enzyme E1 plays a key role in the proteasomal degradation of intracellular proteins [27]. The NEDD8-mediated ubiquitination pathway is regulated by three distinct enzymes: NEDD8-activating enzyme (E1), NEDD8-conjugating enzyme (E2), and NEDD8 ligase enzyme (E3). NEDD8 is first activated by E1; the activated NEDD8 is then transferred from E1 to E2. E2 collaborates with cullin-RING E3 ligases (CRLs) to conjugate NEDD8 to the substrate protein, such as the NF-κB pathway inhibitor [27].

Another protein with altered expression was COMM domain-containing protein 5, which can modulate the activity of Cullin-RING E3 ubiquitin ligases [28]. In this complex, COMMD facilitates NF-κB binding to the ligase, thereby accelerating its ubiquitination and degradation [29].

Meanwhile, in the DEHG group, Cullin-3 was also detected, a component of Cullin-RING E3 ubiquitin ligase complexes involved in protein ubiquitination and part of the ubiquitin-proteasome system [30]. Polyubiquitin-B and Proteasome subunit beta type-6, both proteins involved in the final stage of the ubiquitin-proteasome system, were also identified [31].

The outcomes of activating these proteolytic pathways could be observed in group comparisons. In the differential expression of DEG *versus* PEG, proteins *Myosin Heavy Chain 2*,* Myosin-4*,* Myosin-6*, and *Myosin-7* were all downregulated in DEG compared to PEG. Comparing DEHG to PEG, there was also a decrease in myosin expression in DEHG, but among the identified myosin isoforms, only *Myosin-6* and *Myosin-7* showed decreased levels. Additionally, when comparing DEHG and DEG, Myosin proteins *Myh2*,* Myosin-4*,* Myosin-6*, and *Myosin-7* were reduced in DEG, indicating that although the DEHG Soleus experienced atrophy, it was partially inhibited and less severe than in DEG.

These molecular findings align with the morphological and morphometric data where HMB was applied at the same dosage and in the same atrophic model, showing that the Soleus muscle of both dexamethasone-treated groups experienced atrophy, but it was less severe in the group receiving HMB [23] .

Similarly, in the study by Giron et al. [4], HMB reduced dexamethasone-induced atrophy in the Soleus and Gastrocnemius muscles of Sprague-Dawley rats. However, their protocol involved a lower dexamethasone dose (0.1 mg/kg/day) over a longer period (21 days) [4]. In contrast, in the EDL muscle, Noh et al. [23] found that HMB did not provide a protective effect against dexamethasone-induced atrophy when comparing morphometric data [23].

In our study, for the EDL muscle, both experimental groups showed decreased levels of all identified myosin heavy chain (MyHC) isoforms compared to the placebo (PEG), as expected. When comparing the DEG and DEHG groups, only the *Myosin heavy chain 2 A* (MyHC-2 A) isoform demonstrated relatively higher expression in DEHG than in DEG, whereas all other isoforms did not differ between groups. MyHC-2 A is encoded by the MYH2 gene and is predominantly expressed in fast-twitch oxidative (type IIa) muscle fibers rather than in slow-twitch (type I) fibers, consistent with established fiber-type classification [32]. Fast-twitch fibers, especially those with low oxidative capacity, are known to be more susceptible to glucocorticoid-induced atrophy than oxidative fibers. In both human and rodent models, glucocorticoid treatment causes pronounced atrophy of fast-twitch fibers via enhanced proteolysis and reduced protein synthesis, whereas oxidative fibers are less severely affected under similar conditions [33].

These fiber-specific effects of glucocorticoids may help explain our finding that MyHC-2 A was relatively spared in DEHG. Moreover, the greater efficacy of HMB in the soleus muscle, a muscle with a high proportion of oxidative fibers, may reflect the relative resistance of oxidative fibers to glucocorticoid-mediated catabolism [34].

These observations are in agreement with Baptista et al. [12], who reported that HMB exerted greater protective effects in the soleus muscle, predominantly composed of slow-twitch oxidative fibers, than in the gastrocnemius, which presents a mixed fiber-type composition, in dexamethasone-treated animals [12].

Similarly, Bennett et al. [35] reported no significant effect of HMB on the EDL following limb suspension in aged rats [35]. Although the atrophy model differs, HMB showed significant protective effects in other muscles subjected to the same atrophic protocol [22, 36], but not in the EDL, reinforcing the notion that the responsiveness to HMB may depend on muscle fiber composition and metabolic profile.

Regarding the EDL muscle, we also observed that in DEG, Serine/threonine-protein kinase BRSK2 was overexpressed compared to both PEG and DEHG. BRSK2 activity is induced during fasting conditions to inhibit mTOR and promote autophagy [37]. Dexamethasone-induced autophagy promotes muscle atrophy via mitochondrial clearance [38], and one possible explanation for our findings is that BRSK2 acts as a repressor of NRF2 signaling, a protein that promotes mitochondrial biogenesis [37, 39].

Although HMB was not effective in preventing EDL atrophy and only partially effective in the Soleus, metabolic changes were observed in both muscles, mainly involving proteins related to glucocorticoid receptors, insulin receptors, tumor necrosis factors, and GLUT4. These changes may occur in response to dexamethasone-induced mitochondrial dysfunction and insulin resistance [39].

In the liver, dexamethasone treatment changes the expression of various hepatic enzymes and increases the activity of important enzymes involved in lipid metabolism, gluconeogenesis, amino acid metabolism, and the cytochrome P450 system, which regulates many xenobiotic-metabolizing enzymes [40]. These changes were also observed in our results, showing that dexamethasone impaired glucose uptake, altered the cytochrome P450 system, and affected mitochondrial function, as evidenced by decreased ATP levels.

The involvement of these signaling pathways has been linked to both normal cellular responses, such as survival, proliferation, protein synthesis, and increased mitochondrial respiration, as well as the development of various diseases, including chronic inflammation and cancer [41].

Lipid metabolism, a process heavily influenced by dexamethasone in our study, was not affected by HMB. These results align with those of Bordag et al. (2015), who examined side effects and metabolomic changes caused by dexamethasone and also noted increased lipolysis and elevated levels of gluconeogenic amino acids, indicating enhanced gluconeogenesis (Bordag et al., 2015).

Regarding the histological analysis, we tested a higher dose of dexamethasone to evaluate HMB’s effectiveness in a severe atrophic model. The comparison between HMB and dexamethasone was only examined on red muscle (soleus—with a greater predominance of type I fibers) [12, 23] and mixed muscle (gastrocnemius—containing both type I and type II fibers, such as IIa and IIb) [4]. Our results indicated that HMB could partially protect the soleus muscle even at this dexamethasone dose, but it had no effect on the EDL. According to Nava et al. [42], muscles composed mainly of type 2 fibers (such as the EDL) are more severely affected by dexamethasone, which explains why HMB was unable to prevent or reduce dexamethasone-induced atrophy in the EDL [42].

Regarding the effect of dexamethasone on the extracellular matrix (ECM), the results are inconsistent. Some studies show that dexamethasone decreased collagen I, III, and IV mRNA expression [43, 44], while others indicate that type IV collagen mRNA expression did not decrease, and the overall collagen IV content was even higher in animals treated with DEX [45]. In this study, in GED animals, the proportion of connective tissue remained unchanged compared to GCP, indicating that connective tissue was proportionally reduced following muscle tissue loss. However, when comparing GED and GEDH, the muscle behavior differed: the percentage in the soleus increased, suggesting that the connective tissue experienced a smaller reduction (or none) in its content.

In our study, HMB did not reduce dexamethasone-induced liver changes or prevent myosin loss in the EDL. However, the positive result seen in the Soleus muscle is encouraging and supported by morphometric findings from a previous study [4], suggesting that HMB may be a useful aid in reducing GC-induced atrophy at high doses. Some articles have addressed the careful cost/benefit balance in clinical situations involving high-dose GC use, such as sepsis [46], advanced-stage cancer, and COVID-19 treatment [47]. Therefore, the findings here strongly support the potential for HMB to be used as a therapeutic agent against dexamethasone effects in specific clinical scenarios.

## Conclusion

In conclusion, the dexamethasone administration protocol used leads to the loss of contractile proteins such as myosins in both muscles evaluated. The HMB dose partially prevented myosin loss in the soleus muscle, but did not prevent it in the EDL muscle. Additionally, the same HMB dose was ineffective in reducing dexamethasone-induced hepatic glycogenolysis.

## Supplementary Information

Below is the link to the electronic supplementary material.

Supplementary Material 1

Supplementary Material 2

## Data Availability

The datasets used and/or analyzed during the current study are available from the corresponding author on reasonable request.
