# Supplementary material for: Proteomic Profile of Skeletal Muscles and Liver in a Dexamethasone-Induced Atrophy Model: Insights into the Role of β-Hydroxy-β-Methylbutyrate on Contractile and Metabolic Protein
Source: Cell Biochem Biophys. 2026 Jan 22;84(2):2311–26. doi: 10.1007/s12013-026-01995-4 (PMC13234069; doi:10.1007/s12013-026-01995-4)

Supplemental Figure

Supplemental Figure 1. Representative images of muscles stained with HE: Soleus (A - C) and EDL (D - F). A and D show GPC, B and E show GED, C and F show GEDH. The five-pointed star indicates normal cellular nuclei; thin arrows point to arterioles. The four-pointed stars highlight nuclei toward the center. Sections were taken with a 40x objective. Representative images stained with Masson's trichrome: Soleus (G - I) and EDL (J - L). For GPC, images are G and J; for GED, H and K; and for GEDH, I and L. These section images were obtained with a 20x objective. Symbols: five-pointed star – connective tissue (perimysium); thick arrow - muscle spindle.

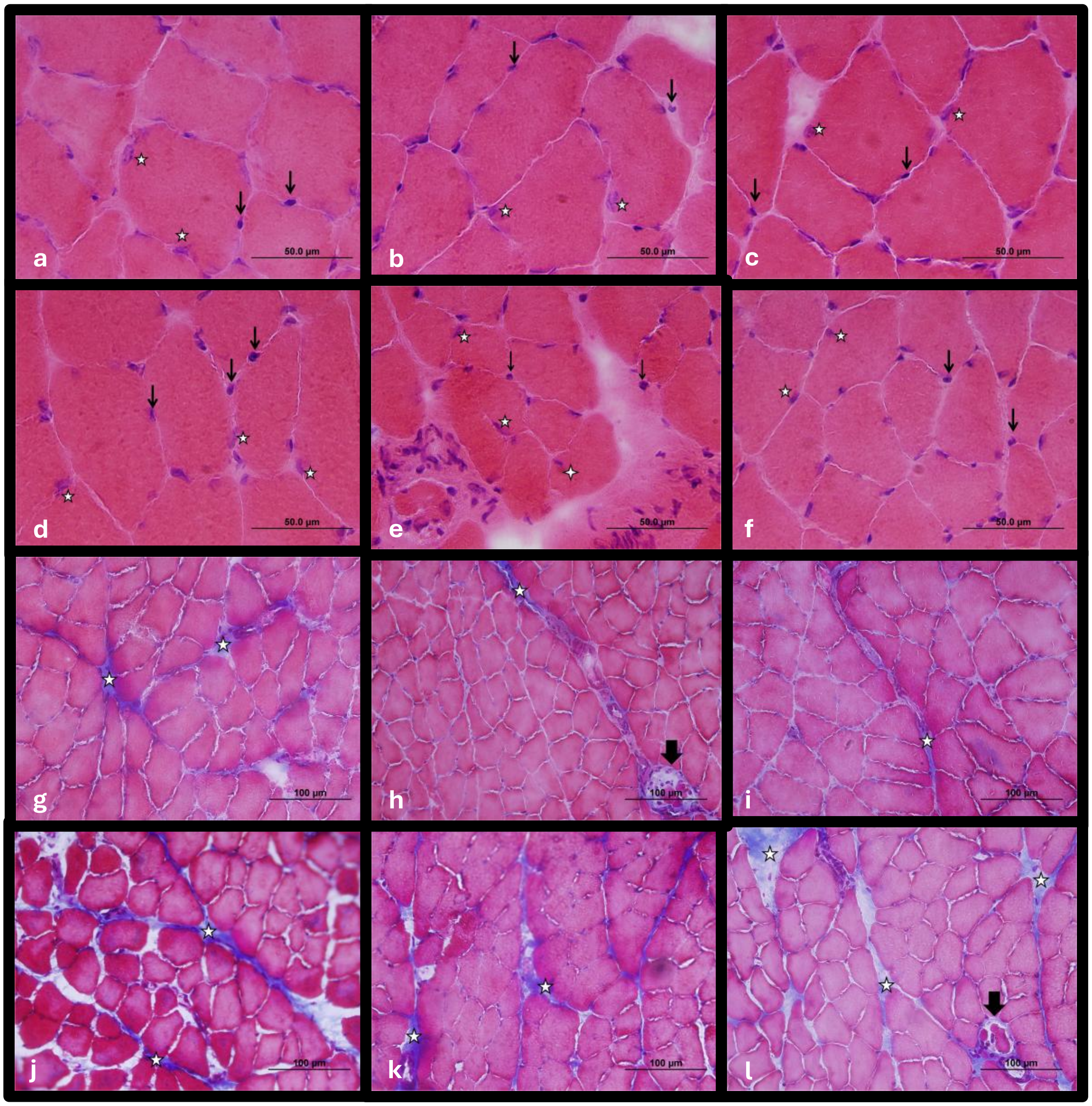

Supplemental Figure 2. Percentage of intramuscular connective tissue in the Soleus and EDL muscles, respectively. Significantly different values were found between GED and GEDH only in the soleus muscle.

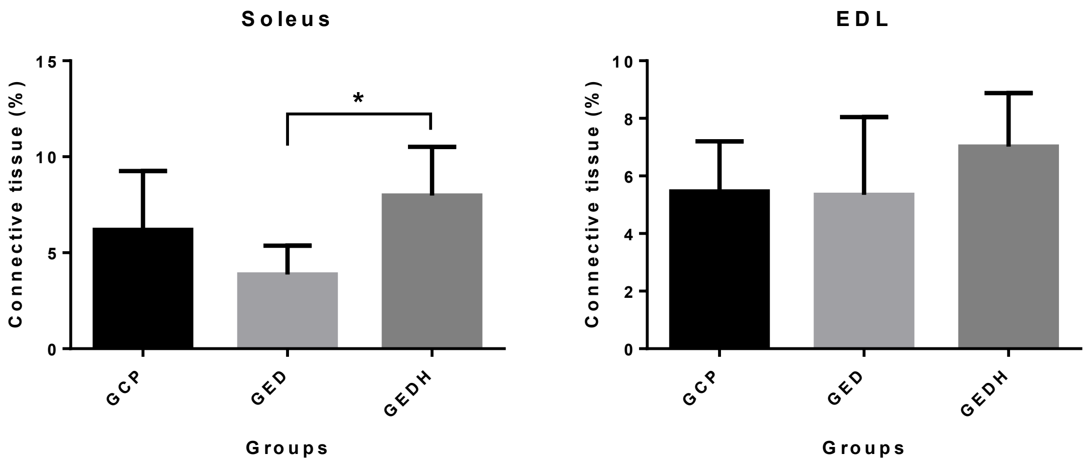

Supplement: Supplementary file 1 — Supplementary Material 1 [file 12013_2026_1995_MOESM1_ESM.pdf]
