# Supplementary material for: Proteomic Profile of Skeletal Muscles and Liver in a Dexamethasone-Induced Atrophy Model: Insights into the Role of β-Hydroxy-β-Methylbutyrate on Contractile and Metabolic Protein
Source: Cell Biochem Biophys. 2026 Jan 22;84(2):2311–26. doi: 10.1007/s12013-026-01995-4 (PMC13234069; doi:10.1007/s12013-026-01995-4)
Supplement: Supplementary file 2 — Supplementary Material 2 [file 12013_2026_1995_MOESM2_ESM.pdf]

Supplementary Materials

Table S1. Unique proteins found in the soleus muscle samples of GEP rats (animals exposed only to placebo treatments).

Accession number	***Protein name***	***PLGS Score***
Q62711	*1-phosphatidylinositol 4,5-bisphosphate phosphodiesterase delta-4*	122.81
P62890	*60S ribosomal protein L30*	46.56
P25108	*Acetylcholine receptor subunit alpha*	108.55
Q5M9H2	*Acyl-Coenzyme A dehydrogenase, very long chain*	125.41
P84079	*ADP-ribosylation factor 1*	44.25
P84083	*ADP-ribosylation factor 5*	104.25
P50475	*Alanine--tRNA ligase, cytoplasmic*	57.9
G3V8I1	*Alkaline phosphatase*	65.03
Q9JJH9	*Alpha-2u globulin*	73.34
Q5U2P3	*AN1-type zinc finger protein 2A*	133.22
B5DEP3	*Anaphase-promoting complex subunit 10*	118.43
P53678	*AP-3 complex subunit mu-2*	119.91
G3V6G9	*Basic leucine zipper transcriptional factor ATF-like 3 O*	60.92
O88996	*BCL-W*	211.34

D3ZAZ5	*Breast cancer anti-estrogen resistance 3 (Predicted)*	112.31
Q8K3P6	*Calcium-binding mitochondrial carrier protein SCaMC-2*	124.95
Q63704	*Carnitine O-palmitoyltransferase 1, muscle isoform*	61.56
B0BNA9	*CCR4-NOT transcription complex subunit 11*	76.93
Q498M7	*CCR4-NOT transcription complex, subunit 4*	117.94
Q9Z2V2	*CD40 ligand*	54.02
G3V7K0	*Ceh-10 homeo domain containing homolog (C. elegans), isoform CRA_a*	68.84
Q1WIM2	*Cell adhesion molecule 2*	84.05
P51802	*Chloride channel protein ClC-Kb*	157.63
Q9R1E9	*Connective tissue growth factor*	215.74
Q99ME0	*CXC chemokine RTCK1*	63.28
D3ZD09	*Cytochrome c oxidase subunit 6B1*	1299.77
G3V7T8	*Discs, large (Drosophila) homolog-associated protein 3, isoform CRA_b*	70.57
P97838	*Disks large-associated protein 3*	75.89
Q62798	*Dorsal root ganglia homeobox protein*	49.44
Q8CJB9	*E3 ubiquitin-protein ligase BRE1B*	52.83
Q76IJ9	*ELAV (Embryonic lethal, abnormal vision, Drosophila)-like 3 (Hu antigen C), isoform CRA_b*	74.43
Q3MIE0	*Enoyl-CoA hydratase domain-containing protein 3, mitochondrial*	181.55

Q6MG81	*FK506-binding protein-like*	60.89
P06883	*Glucagon*	175.81
D3Z8F7	*Glucokinase activity, related sequence 1 (Predicted)*	43.89
D3ZY68	*Glutamyl-tRNA(Gln) amidotransferase subunit C, mitochondrial O*	173.64
O08730	*Glycogenin-1*	97.37
Q5BJQ5	*GTP-binding protein Rit2*	191.71
Q07439	*Heat shock 70 kDa protein 1A/1B*	114.54
D3ZVM5	*Heat shock protein 70kDa 12B (Predicted)*	168.89
Q64268	*Heparin cofactor 2*	50.24
Q78ZR5	*Homeodomain-only protein*	191.38
Q9WVK7	*Hydroxyacyl-coenzyme A dehydrogenase, mitochondrial*	133.09
P15693	*Intestinal-type alkaline phosphatase 1*	65.03
D4ACI9	*Intraflagellar transport 88 homolog (Chlamydomonas) (Predicted)*	62.83
G3V779	*Ladinin (Predicted), isoform CRA_b*	71.25
D4A6D8	*Leucine-rich repeat transmembrane neuronal protein 1*	57.6
O88204	*Low-density lipoprotein receptor-related protein 3*	118.19
Q6QI20	*LRRGT00188*	87.42
Q5XIC4	*Maspardin*	142.76
G3V8M0	*Melanophilin*	138.18

P04633	*Mitochondrial brown fat uncoupling protein 1*	66.17
Q66HP8	*Mitochondrial carnitine/acylcarnitine carrier protein*	42.58
Q9WTY9	*Mitogen-activated protein kinase 13*	79.22
Q63562	*Mitogen-activated protein kinase kinase kinase 8*	74.1
G3V840	*Mitogen-activated protein kinase kinase kinase 8*	74.1
B0BNJ3	*Morc4 protein*	62.95
D3ZC22	*Myotubularin related protein 6 (Predicted), isoform CRA_a*	114.67
B2RZD6	*Ndufa4 protein*	643.48
D4A6Y3	*Neurexin-1*	56.09
Q2YDU6	*Nuclear prelamin A recognition factor*	132.81
Q63083	*Nucleobindin-1*	55.35
D4A3K0	*Olfactory receptor*	41.7
Q62782	*Paired mesoderm homeobox protein 2A*	68.72
D4A881	*Phosphoinositide phospholipase C*	122.81
D3ZJM8	*Poly(A)-specific ribonuclease*	64.87
F7FA48	*Progesterone receptor*	127.32
Q6NX65	*Programmed cell death protein 10*	56.69
P09320	*Prolactin-4A1*	96.19
F1M9V5	*Prostate and testis expressed 2*	279.6

M0RAF7	*Protein 3110082J24Rik*	63.62
D3ZPI6	*Protein Abca12*	60.26
F1LTT8	*Protein C2cd4a*	58.12
D4A9X9	*Protein Cenpk*	42.32
D4A3V5	*Protein Cgnl1*	48.33
D3ZC63	*Protein Cmpk2*	65.5
D3ZG48	*Protein Ddx50*	189.86
B2RZ42	*Protein FAM150B*	71.72
Q5XIN5	*Protein FAM228A*	295.34
F1LQ27	*Protein Fam98c*	139.65
D4AD65	*Protein Figla*	103.35
A0A096MK92	*Protein Ift88*	62.83
F1LWL3	*Protein LOC100362226 (Fragment)*	80.77
M0RCT6	*Protein LOC100363427 (Fragment)*	106.43
M0R8W3	*Protein LOC100909612 (Fragment)*	66.17
M0R8J8	*Protein LOC100910259*	58.73
G3V8N0	*Protein LOC100910912*	82.61
M0R6B6	*Protein LOC100911393 (Fragment)*	71.63
F1LYK5	*Protein LOC100911395 (Fragment)*	72.31

Q1RP74	*Protein LOC100911774*	77.08
M0R4X7	*Protein LOC100912272*	168.18
M0R983	*Protein LOC688320*	82.44
A0A096MK24	*Protein Morc4*	62.95
D3ZM65	*Protein Ncoa7*	114.49
M0RBR8	*Protein Pip5kl1 (Fragment)*	158.39
F7EPH4	*Protein Ppa1*	126.67
Q66HG8	*Protein Red*	69.87
D3ZQ82	*Protein Rfx1*	57.98
B1WC95	*Protein RGD1560010*	81.09
D3ZTZ3	*Protein Rnf222*	129.48
D3Z991	*Protein Rps6kc1*	110.43
D4ABK0	*Protein Sgcz (Fragment)*	66.76
D4A6B3	*Protein Slamf6*	58.71
D4A589	*Protein Smg6*	48.61
D3Z8L2	*Protein Spata33*	57.39
D3ZC46	*Protein Tcf25*	108.83
Q5XIF0	*Protein Tex264*	55.4
D3Z9M6	*Protein Tmem114*	82.81

D4A5W0	*Protein Toe1*	116.49
F1M5N2	*Protein Vsig10l*	100.45
D4A8F0	*Protein Zbtb49*	88.69
D3ZSN4	*Protein Zfp330*	118.62
P49432	*Pyruvate dehydrogenase E1 component subunit beta, mitochondrial*	43.4
B1H241	*Resistance to inhibitors of cholinesterase 8 homolog A (C. elegans)*	55.62
Q63639	*Retinal dehydrogenase 2*	53.78
Q9Z1J8	*SEC14-like protein 3*	93.34
Q64627	*Sialidase-2*	58.61
Q7TP15	*S-methyl-5'-thioadenosine phosphorylase*	93.58
P23978	*Sodium- and chloride-dependent GABA transporter 1*	62.79
D4AE35	*Sulfotransferase*	100.36
O88583	*Suppressor of cytokine signaling 3*	67.35
Q4QQU6	*Survival of motor neuron-related-splicing factor 30*	76.36
Q80ZG1	*Synembryn-A*	55.62
Q5HZA9	*Transmembrane protein 126A*	235.65
Q6QA27	*Tripartite motif-containing protein 44*	62.77
Q5XIF6	*Tubulin alpha-4A chain*	67.26
Q6P9T8	*Tubulin beta-4B chain*	179.28

B5DEI4	*Ubiquitin-conjugating enzyme E2 W*	71.63
Q5M9F0	*UPF0705 protein C11orf49 homolog*	166.14
P84889	*Vang-like protein 2*	47.15
P45953	*Very long-chain specific acyl-CoA dehydrogenase, mitochondrial*	125.41

Table S2. Unique proteins found in the soleus muscle samples of GED rats (present in the samples of animals exposed only to 1 mg/kg/day of Dexamethasone for 10 days).

Accession number	***Protein name***	***PLGS Score***
F1LMK0	*A kinase (PRKA) anchor protein 1, isoform CRA_a*	61.37
D3ZRE6	*AarF domain containing kinase 2 (Predicted)*	89.61
B3DMA2	*Acyl-CoA dehydrogenase family member 11*	41.78
D4A3N4	*Adenylate cyclase 1 (Predicted)*	55.55
P15207	*Androgen receptor*	39.59
D3ZCH1	*Ankyrin repeat domain 12 (Predicted), isoform CRA_b*	50
P12527	*Arachidonate 5-lipoxygenase*	49.29
Q6P6T7	*Beta-1,3-N-acetylglucosaminyltransferase*	72.98
Q9R0T4	*Cadherin-1*	79
F1LPF6	*Calcium-transporting ATPase*	80.92
Q5XI33	*Cell death activator CIDE-3*	42.58
Q5XIN9	*Coiled-coil domain-containing protein 81*	46.23
Q9ERR2	*COMM domain-containing protein 5*	51.21
D3ZWT9	*C-type lectin domain family 6 member A*	110.94
B2RZ50	*Cyclin-dependent kinase inhibitor 3*	107.32

Q5U2T2	*Dehydrodolichyl diphosphate synthase*	116.03
Q6P6R2	*Dihydrolipoyl dehydrogenase, mitochondrial*	86.98
P14740	*Dipeptidyl peptidase 4*	77.41
Q5RKI3	*DNA polymerase lambda*	60.69
Q499W2	*DNA replication complex GINS protein SLD5*	43.18
Q6IML7	*DnaJ homolog subfamily C member 27*	60.74
Q5M834	*Dysbindin*	76.92
Q68FR6	*Elongation factor 1-gamma*	44.38
Q6PCU4	*Emd protein*	75.11
Q3T1K5	*F-actin-capping protein subunit alpha-2*	81.34
M0R6L9	*Ferritin*	44.18
Q62997	*GDNF family receptor alpha-1*	46.45
P06536	*Glucocorticoid receptor*	52.1
Q8K1Q0	*Glycylpeptide N-tetradecanoyltransferase 1*	89
P48317	*Growth arrest and DNA damage-inducible protein GADD45 alpha*	94.73
Q9QYW4	*Growth differentiation factor 9*	111.23
Q5XIA8	*Growth hormone-inducible transmembrane protein*	54.4
P62882	*Guanine nucleotide-binding protein subunit beta-5 O*	76.11
M0R4T9	*Histone acetyltransferase*	75.5

G3V8Y8	*Huntingtin interacting protein 1, isoform CRA_a*	76.84
P52296	*Importin subunit beta-1*	62.09
D4AD37	*Inositol monophosphatase 3*	60.12
Q68FR3	*Integrator complex subunit 12*	101.73
Q7TP98	*Interleukin enhancer-binding factor 2*	88.04
Q4QQS0	*Interleukin-1 receptor-associated kinase-like 2*	55.37
P98089	*Intestinal mucin-like protein (Fragment)*	43.4
D3ZRI4	*Iroquois related homeobox 4 (Drosophila) (Predicted)*	67.29
D4AB89	*Kinesin-like protein*	73.24
Q9QX69	*LanC-like protein 1*	111.47
Q5U2Y9	*Lebercilin*	92.12
Q4V7E8	*Leucine-rich repeat flightless-interacting protein 2*	66.66
Q63010	*Liver carboxylesterase B-1*	52.25
Q6QI33	*LRRGT00175*	99.44
Q6AY59	*Molybdopterin synthase catalytic subunit*	52.76
M0R6C7	*Mucin-2*	43.4
E9PT87	*Myosin light chain kinase 3*	54.29
O08557	*N(G),N(G)-dimethylarginine dimethylaminohydrolase 1*	91.83
D3ZS58	*NADH dehydrogenase [ubiquinone] 1 alpha subcomplex subunit 2*	83.7

Q6DKG0	*N-alpha-acetyltransferase 35, NatC auxiliary subunit*	43.31
Q9Z1A5	*NEDD8-activating enzyme E1 regulatory subunit*	49.38
P58821	*Neuron-specific vesicular protein calcyon*	68.6
Q62609	*Noelin*	87.3
D3ZN70	*Nuclear receptor corepressor 1*	122.15
Q5XIG4	*OCIA domain-containing protein 1*	284.95
M0R7X1	*Olfactory receptor*	75.9
P83860	*Orexigenic neuropeptide QRFP*	147.8
D3ZHS8	*Protein Amer3*	195.89
D3ZGR5	*Protein Ankrd12*	45.5
D3ZER2	*Protein Bfsp2*	54.13
D3ZSR7	*Protein Ccdc102a*	46.96
M0R9C5	*Protein Ccdc160*	73.64
F7ESU6	*Protein Ccnb2*	68.28
F1M7J7	*Protein Cep250 (Fragment)*	28.59
D3ZXM6	*Protein Clec4n*	110.94
B2RZ68	*Protein Dcaf7*	47.32
D3Z9D4	*Protein Dcdc2c*	61.94
D4A903	*Protein Dhdh*	44.94

D4AD15	*Protein Eif4g1*	43.94
E9PSY8	*Protein Eps15*	52.43
M0RBH9	*Protein Gas1*	136.03
D3ZTB7	*Protein Gm17190*	56.18
M0R8F6	*Protein Gm4978 (Fragment)*	46.12
D3ZY81	*Protein Gzmn*	53.62
D4A7Q7	*Protein Hunk*	109.19
F1M147	*Protein Hyls1 (Fragment)*	81.7
F1M293	*Protein Il15ra*	121.04
D3ZD85	*Protein Kat6b*	68.46
D3ZVK8	*Protein Lilrb3l*	51.05
F1MA06	*Protein LOC100362176*	101.68
D4ABA3	*Protein LOC100362909*	82.11
M0RD03	*Protein LOC100912571 (Fragment)*	43.94
Q5XIK0	*Protein LOC502684*	62.36
F1M337	*Protein LOC681364 (Fragment)*	98.18
D3ZV82	*Protein LOC685067*	74.1
D3ZF00	*Protein LOC685621*	49.99
D3ZV94	*Protein LOC691161*	146.5

E9PSW6	*Protein Lrch4*	54.1
D3ZFB2	*Protein Luc7l3*	128.33
D3ZTY6	*Protein Mfsd9*	56.69
A0A096MKH9	*Protein Morc3*	52.68
D4AEN7	*Protein Mroh2b*	39.24
D4A4A9	*Protein Mrpl19*	93.64
D3ZWK2	*Protein Parvg*	41.1
Q8K3F3	*Protein phosphatase 1 regulatory subunit 14B*	103.74
D4A9R2	*Protein Pkhd1l1*	52.77
Q6AYG3	*Protein prune homolog*	60.63
M0R3X1	*Protein Psapl1*	105.43
F1M7S7	*Protein Rab17 (Fragment)*	97.09
D3ZCU8	*Protein Rab33a*	136.95
D3ZFR9	*Protein Rdh13*	95.61
Q498C8	*Protein RER1*	91.98
D4A2T4	*Protein RGD1305184*	57.3
F1LQD9	*Protein RGD1305455*	109.68
F1LWA9	*Protein RGD1308742 (Fragment)*	86.58
D3ZBU7	*Protein RGD1310819 (Fragment)*	27.36

F1LU12	*Protein RGD1564865 (Fragment)*	73.74
D4AB71	*Protein RGD1565222*	43.03
D3ZEQ6	*Protein RGD1565283*	57.5
Q4TU79	*Protein Rhox3*	58.36
F1LVT6	*Protein Senp1 (Fragment)*	48.96
D3ZM80	*Protein Six2 (Fragment)*	53.3
D3ZWL1	*Protein Skil*	41.84
D3ZBS9	*Protein Smarcd1*	67.37
Q4V7E4	*Protein Sox18*	80.04
B4F7D6	*Protein Stk11ip*	85.35
B5DF13	*Protein Trim59*	58.95
D4A147	*Protein Ugt2a3*	118.33
M0R988	*Protein Zfp106*	47.5
D3ZLD2	*Protein Zmat5*	94.32
M0RBA0	*Pulmonary surfactant-associated protein D*	126.12
F1LSH4	*Pyrin*	74.32
P35281	*Ras-related protein Rab-10*	105.35
Q6WN19	*Reticulon*	115.56
Q4QQT0	*RNA pseudouridylate synthase domain-containing protein 4*	118.55

P11507	*Sarcoplasmic/endoplasmic reticulum calcium ATPase 2*	80.92
Q64666	*Serotonin N-acetyltransferase*	51.7
Q925Q9	*SH3 domain-containing kinase-binding protein 1*	135.75
P0DJJ3	*SH3-containing GRB2-like protein 3-interacting protein 1*	47.55
G3V9I9	*Splicing factor, arginine/serine-rich 12, isoform CRA_b*	46.74
Q9JKL7	*Splicing regulatory glutamine/lysine-rich protein 1*	52.87
Q5PPL3	*Sterol-4-alpha-carboxylate 3-dehydrogenase, decarboxylating*	79.53
D4AB66	*Stonin-2*	77.44
O70377	*Synaptosomal-associated protein 23*	89.74
P21707	*Synaptotagmin-1*	53.9
P47861	*Synaptotagmin-5*	61.11
Q04666	*Transcription factor HES-1*	48.79
P86252	*Transcriptional activator protein Pur-alpha (Fragments)*	165.17
D3ZLN0	*Transformer-2 protein homolog beta*	82.9
Q6AY76	*Transmembrane protein 248*	61.87
P97710	*Tyrosine-protein phosphatase non-receptor type substrate 1*	71.21
D3ZLQ8	*Ubiquitin carboxyl-terminal hydrolase*	52.99
Q5PPF5	*Zinc finger CCCH-type with G patch domain-containing protein*	50.11
Q4KLI1	*Zinc finger protein with KRAB and SCAN domains 1*	66.49

Table S3. Unique proteins found in the soleus muscle samples of GEDH rats (animals exposed to 1 mg/kg/day of Dexamethasone and 300 mg/kg/day of HMB for 10 days).

Accession number	***Protein name***	***PLGS Score***
P29266	*3-hydroxyisobutyrate dehydrogenase, mitochondrial*	62.72
F1LWG8	*5-hydroxytryptamine receptor 2B*	78.6
Q7TP79	*Aa2-245*	64.73
M0RAP9	*Acetyltransferase component of pyruvate dehydrogenase complex*	51.14
P49911	*Acidic leucine-rich nuclear phosphoprotein 32 family member A*	49.79
G3V7G3	*Activity-dependent neuroprotector homeobox protein*	51.09
P0C6C0	*A-kinase anchor protein SPHKAP*	71.94
P25409	*Alanine aminotransferase 1*	76.43
D3ZP53	*Ankyrin repeat and sterile alpha motif domain-containing protein 1B*	215.49
Q5U318	*Astrocytic phosphoprotein PEA-15*	177.13
Q06647	*ATP synthase subunit O, mitochondrial*	81.2
Q03344	*ATPase inhibitor, mitochondrial*	75.27
Q32ZG9	*Beta-defensin 129*	44.02
Q4V7C8	*Centrosomal protein of 55 kDa*	52.49
Q4V891	*Centrosomal protein POC5*	40.22

P98087	*Cerebellin-2*	47.2
P32738	*Choline O-acetyltransferase*	36.37
G3V9X4	*Cholinergic receptor, nicotinic, alpha polypeptide 5, isoform CRA_b*	46.46
D3ZUQ3	*Coronin*	67.33
Q91ZN1	*Coronin-1A*	68.95
B5DF89	*Cullin-3*	75.53
D3ZG85	*Cyclin-dependent kinase-like 5*	40.16
Q8VII2	*Cystatin-12*	215.03
P12075	*Cytochrome c oxidase subunit 5B, mitochondrial*	241.37
D3ZHZ6	*Cytochrome P450 4A10*	79.08
Q63150	*Dihydropyrimidinase*	46.22
A0A096MJ42	*Disks large homolog 3*	53.64
E9PU42	*Down syndrome critical region gene 3 (Predicted), isoform CRA_c*	83.72
Q5XHX3	*Enabled homolog (Drosophila)*	107.2
Q6P3V8	*Eukaryotic translation initiation factor 4A1*	113.81
P05369	*Farnesyl pyrophosphate synthase*	69.45
Q9EST9	*Fibroblast growth factor 20*	52.95
P18427	*Follitropin subunit beta*	66.08
P59647	*FXYD domain-containing ion transport regulator 5*	54.16

P10683	*Galanin peptides*	80.3
D3ZD47	*Gene model 672, (NCBI) (Predicted)*	101.43
O35977	*Glial cell line derived neurotrophic factor family receptor alpha 2*	45.46
Q9R0A8	*HAUS augmin-like complex subunit 1*	42.35
F7EHL9	*Hermansky-Pudlak syndrome 5 protein*	44.87
M0R4T1	*Homeobox protein cut-like 1*	53.44
B5DF15	*Interleukin 17 receptor B*	44.33
Q62751	*Iron-responsive element-binding protein 2*	42.5
A1A5Q5	*Lysine-specific demethylase 4D*	37.92
Q62667	*Major vault protein*	84.92
Q66H84	*MAP kinase-activated protein kinase 3*	54.82
P97592	*Mast cell protease 4*	126.44
Q6AXU8	*Methyltransferase-like protein 6*	72.39
G3V6D9	*Na(+)/H(+) exchange regulatory cofactor NHE-RF*	83.8
P20420	*Neuronal acetylcholine receptor subunit alpha-5*	42.2
P70563	*Nucleoside diphosphate-linked moiety X motif 6*	95.54
Q5M7U2	*Origin recognition complex, subunit 5-like (Yeast)*	46.31
Q920Q0	*Paralemmin-1*	76.64
G3V8W2	*Phosphatidate cytidylyltransferase*	50.05

F1LML2	*Polyubiquitin-B*	120.63
Q63429	*Polyubiquitin-C*	120.63
Q5U317	*Pre-mRNA 3'-end-processing factor FIP1*	91.96
P09321	*Prolactin-3B1*	105.09
P28073	*Proteasome subunit beta type-6*	174.26
D4ADV7	*Protein 4930402K13Rik*	173.61
D4A576	*Protein Abcd4*	47.56
D3ZA74	*Protein Adamts14*	51.53
D3Z9L0	*Protein Agk*	62.89
D4A9Q2	*Protein Amot*	73.51
D4A1H6	*Protein Astn2*	107.53
D4ADK4	*Protein Cachd1*	73.9
F1M0U1	*Protein Ccdc73*	46.71
D3ZTW6	*Protein Ccser1*	140.33
F1MAH6	*Protein Cdh11*	41.82
D3ZSW4	*Protein Crnn*	113.43
D3ZCZ3	*Protein Cys1*	147.46
M0R6L8	*Protein Dnajc19*	39.4
D3ZPV7	*Protein Foxred1*	57.91

M0R3K5	*Protein Gdf5*	74.18
F1M019	*Protein Gse1 (Fragment)*	39.24
D4A1Z4	*Protein Gtf3c2*	42.77
A0A096MJ97	*Protein Heatr9*	44.32
F7EVC6	*Protein Hebp1*	256.53
D3ZFH5	*Protein Itih2*	73.53
D4AAW0	*Protein Kansl1*	47.92
G3V7R5	*Protein LOC100174910*	56.26
D4AEC9	*Protein LOC100360330*	45.01
D3ZPN7	*Protein LOC100360604*	51.93
D4A7B4	*Protein LOC100360611*	51.93
G3V9Z2	*Protein LOC100360645 (Fragment)*	120.63
D3Z9G3	*Protein LOC100361103*	51.93
D4ABI9	*Protein LOC100361556*	126.69
D3ZZH9	*Protein LOC100362779*	51.93
M0R5I1	*Protein LOC100909556 (Fragment)*	86.21
Q6MGB1	*Protein LOC100911800*	75.03
F1LWD5	*Protein LOC100912365*	81.59
D4A1P5	*Protein LOC102551064*	101.4

F1LVC4	*Protein LOC102556876*	58.57
M0R7U9	*Protein LOC681658*	41.09
F1LRA7	*Protein Map3k9*	81.74
D4A6P2	*Protein Mfsd4*	59.11
D3ZBT5	*Protein Mtcl1 (Fragment)*	41.24
D4A7U8	*Protein Myoz1*	109.7
D3ZH40	*Protein Otud7b*	76.04
D4A455	*Protein Pcdhga10*	57.47
D3ZX53	*Protein Prelid2*	57.18
G3V7P5	*Protein RGD1564614*	77.3
D3ZN86	*Protein RGD1565323*	61.85
F1LU69	*Protein Rps27l3*	120.63
F1M513	*Protein Rufy4*	48.79
D4A2I9	*Protein Sorcs3*	71.41
M0R7I1	*Protein Srbd1*	51.2
G3V8D0	*Protein St8sia3*	42.55
D4A3D9	*Protein Stk32c*	58.33
D3Z805	*Protein Sun5*	39.76
D3ZES0	*Protein Tet3*	45.99

F1LU55	*Protein Tmem249 (Fragment)*	55.12
D4ADT4	*Protein Tmem44*	40.28
A0A096MJG3	*Protein Tnfsf13b*	98.85
F1M5H6	*Protein Tp53bp2 (Fragment)*	52.18
Q66H79	*Protein Trim32*	46.51
D3ZA17	*Protein Trim46*	88.52
M0RBH7	*Protein Ttc16 (Fragment)*	73.2
D3ZGZ9	*Protein Wdr59*	40.34
A0A096MJG6	*Protein YIPF6*	61.8
P50399	*Rab GDP dissociation inhibitor beta*	47.98
Q9Z2P5	*Receptor-interacting serine/threonine-protein kinase 3*	80.8
P97844	*Regulator of G-protein signaling 1*	75.75
F1LMZ1	*Solute carrier family 15 member 1*	79.25
Q6AXV6	*Spermatid-associated protein*	80.29
F7EPE0	*Sulfated glycoprotein 1*	66.19
P07632	*Superoxide dismutase [Cu-Zn]*	212.74
Q6MFY8	*Tripartite motif-containing protein 40*	98.07
P68370	*Tubulin alpha-1A chain*	53.12
P62982	*Ubiquitin-40S ribosomal protein S27a*	120.63

P62986	*Ubiquitin-60S ribosomal protein L40*	120.63
D3ZPH4	*Uncharacterized protein (Fragment)*	197.49
M0RCB8	*Uncharacterized protein (Fragment)*	170.16
P97524	*Very long-chain acyl-CoA synthetase*	79.69
Q62634	*Vesicular glutamate transporter 1*	47.8
Q9Z2L0	*Voltage-dependent anion-selective channel protein 1*	92.97
Q5U2S3	*Zinc finger with UFM1-specific peptidase domain protein*	45.83

Table S4. Proteins identified in the soleus muscle of animals with differential expression in the GED group compared to the GEP group (upregulated or downregulated).

Accession number	***Protein name***	***PLGS Score***	**Protein Expression Ratio**<br>**DEG:PEG**
P10719	*ATP synthase subunit beta, mitochondrial*	568.7	0.794533599
P14141	*Carbonic anhydrase 3*	2820.95	0.718923724
P04797	*Glyceraldehyde-3-phosphate dehydrogenase*	105.77	0.65050909
Q9ESV6	*Glyceraldehyde-3-phosphate dehydrogenase, testis-specific*	129.68	0.670320042
P01946	*Hemoglobin subunit alpha-1/2*	4254.78	0.748263574
P02091	*Hemoglobin subunit beta-1*	6960.19	0.763379486
G3V6E1	*Myosin heavy chain 2*	310.76	0.843664815
Q29RW1	*Myosin-4*	554.45	0.860707971
G3V885	*Myosin-6*	1679.82	0.869358235
P02564	*Myosin-7*	2185.8	0.869358235
F1LRV9	*Protein Myh2*	481.03	0.852143792
P02770	*Serum albumin*	360.51	0.697676316
P00564	*Creatine kinase M-type*	1006.45	0.826959136
D3ZCV0	*Protein Actn2*	869.89	0.895834136

Q63610	*Tropomyosin alpha-3 chain*	930.56	0.835270205
P16409	*Myosin light chain 3*	3911.8	0.904837417
P08733	*Myosin regulatory light chain 2, ventricular/cardiac muscle isoform*	2999.3	0.886920439
P63259	*Actin, cytoplasmic 2*	3855.28	0.96078944
O88751	*Calcium-binding protein 1*	102.88	0.904837417
P58775	*Tropomyosin beta chain*	1295.46	0.886920439
D3ZHA7	*Similar to Myosin light chain 1 slow a (Predicted)*	206.83	0.576949804
P04692	*Tropomyosin alpha-1 chain*	1035.32	0.878095435
O88752	*Epsilon 1 globin*	3778.99	2.117000017

Expression ratios with values lower than 1 represent downregulated proteins, whereas ratio values higher than 1 correspond to upregulated proteins. The identified proteins are divided into downregulated (shown at the top of the table) and upregulated (shown at the bottom of the table), organized in alphabetical order based on the protein name.

Table S5. Proteins identified in the Soleus muscle of animals with differential expression in the PEG group compared to the DEHG group (upregulated or downregulated).

Accession number	***Protein name***	***PLGS Score***	**Protein Expression Ratio**<br><br>**PEG:DEHG**
O88752	*Epsilon 1 globin*	3778.99	0.718923724
P04466	*Myosin regulatory light chain 2, skeletal muscle isoform*	722.22	0.794533599
Q64428	*Trifunctional enzyme subunit alpha, mitochondrial*	54.61	0.304221247
P00564	*Creatine kinase M-type*	1006.45	1.1502738
F1M3U4	*Gp_dh_N domain-containing protein*	283.63	1.271249144
D3Z9K3	*Myosin regulatory light chain 2, ventricular/cardiac muscle isoform*	2086.52	1.246076729
P97541	*Heat shock protein beta-6*	441.68	1.363425117
P08733	*Myosin regulatory light chain 2, ventricular/cardiac muscle isoform*	2999.3	1.185304853
P00330	*Alcohol dehydrogenase 1*	53.7	2.435129616
P23928	*Alpha-crystallin B chain*	1117.97	1.309964465
Q2Q0I9	*Fibronectin type III domain-containing protein 1*	75.1	1.404947596
P11517	*Hemoglobin subunit beta-2*	6382.82	1.491824707
D3ZCI9	*Myosin light chain 10*	2245.62	1.233678052
P14141	*Carbonic anhydrase 3*	2820.95	1.271249144

M0R590	*Glyceraldehyde-3-phosphate dehydrogenase*	105.77	1.271249144
P01946	*Hemoglobin subunit alpha-1/2*	4254.78	1.476980773
P02091	*Hemoglobin subunit beta-1*	6960.19	1.896480852
G3V885	*Myosin-6*	1679.82	1.11627807
G3V8B0	*Myosin-7*	2192.97	1.11627807
E9PSN4	*Protein Zc3h13*	43.34	1.584073998
P02770	*Serum albumin*	360.51	1.616074385

Ratios with values below 1 represent downregulated proteins, while ratios above 1 correspond to upregulated proteins. The identified proteins are divided into downregulated proteins at the top of the table and upregulated proteins at the bottom.

Table S6. Table of proteins identified in the Soleus muscle of animals with differential expression in the DEG group compared to the DEHG group (upregulated or downregulated).

Accession number	***Protein name***	***PLGS Score***	**Protein Expression Ratio**<br>**DEG:DEHG**
Q6AY94	*Complex I assembly factor TIMMDC1, mitochondrial*	66.83	0.172045
Q63560	*Microtubule-associated protein 6*	112.63	0.346456
F1LRV9	*Protein Myh2*	481.03	0.843665
D3ZQT7	*Protein Tet3*	94.54	0.015299
Q64428	*Trifunctional enzyme subunit alpha, mitochondrial*	54.61	0.182684
Q63610	*Tropomyosin alpha-3 chain*	930.56	0.818731
F1M8F6	*Uncharacterized protein (Fragment)*	298.26	0.810584
F1M789	*Uncharacterized protein*	198.3	0.88692
G3V6E1	*Uncharacterized protein*	310.76	0.802519
F1LMU0	*Myosin-4*	553.36	0.88692
F7FK40	*Tropomyosin 1, alpha, isoform CRA_c*	415.85	0.794534
P04692	*Tropomyosin alpha-1 chain*	1035.32	0.83527
P09495	*Tropomyosin alpha-4 chain*	558.9	0.810584
P02563	*Myosin-6*	1279.86	0.951229

P58775	*Tropomyosin beta chain*	1295.46	0.878095
P10719	*ATP synthase subunit beta, mitochondrial*	568.7	0.88692
P14141	*Carbonic anhydrase 3*	2820.95	0.913931
P04466	*Myosin regulatory light chain 2, skeletal muscle isoform*	722.22	0.802519
P02564	*Myosin-7*	2185.8	0.970446
E9PSN4	*Protein Zc3h13*	43.34	1.632316
O88752	*Epsilon 1 globin*	3778.99	1.521962
P02091	*Hemoglobin subunit beta-1*	6960.19	1.447735

Ratios with values below 1 represent downregulated proteins, while ratios above 1 correspond to upregulated proteins. The identified proteins are divided into downregulated proteins at the top of the table and upregulated proteins at the bottom of the table.

Table S7. Unique proteins found in EDL muscle samples from PEG rats (animals exposed only to placebo treatments).

Accession number	***Protein name***	***PLGS Score***
P63324	*40S ribosomal protein S12*	191.72
P62083	*40S ribosomal protein S7*	73.43
Q63003	*5E5 antigen*	48.02
P63039	*60 kDa heat shock protein, mitochondrial O*	35.04
B2GV68	*Alkbh2 protein*	120.14
Q09326	*Alpha-1,6-mannosyl-glycoprotein 2-beta-N-acetylglucosaminyltransferase*	80.93
Q9Z1P2	*Alpha-actinin-1*	81.07
B5DEX9	*Arid3a protein*	54.05
Q5U318	*Astrocytic phosphoprotein PEA-15*	92.07
P13721	*Beta-galactoside alpha-2,6-sialyltransferase 1*	56.21
D4ABV5	*Calmodulin*	565.44
Q64537	*Calpain small subunit 1*	59.45
P14141	*Carbonic anhydrase 3*	151.84
A0S5V8	*Chitotriosidase*	46.09
D3ZUQ3	*Coronin*	117.57

O35828	*Coronin-7*	128.96
D4ADD0	*Distal-less homeobox 3 (Predicted)*	107.37
G3V6F5	*ElaC homolog 2 (E. coli)*	45.3
G3V771	*Endothelin 2*	107.16
D3ZAQ9	*Excision repair cross-complementing rodent repair deficiency, complementation group 1, isoform CRA_a*	127.12
B2RZ28	*F-box protein 28*	149.63
Q5XI13	*Glutamate-rich WD repeat-containing protein 1*	43.42
D3Z9R9	*Goosecoid-like (Predicted)*	55.09
D4A9P9	*Gup1, glycerol uptake/transporter homolog (Yeast) (Predicted)*	49.3
D4A426	*Homeo box B2 (Predicted)*	89.67
P97830	*Homeobox protein unc-4 homolog*	60.4
Q80W90	*LIM/homeobox protein Lhx9*	135.7
P42123	*L-lactate dehydrogenase B chain*	44.82
Q920F5	*Malonyl-CoA decarboxylase, mitochondrial*	31.21
B5DEG0	*Melanoma associated antigen (Mutated) 1-like 1*	30.43
Q52KJ8	*Methionine-R-sulfoxide reductase B1*	161.1
Q497B7	*NK-3 transcription factor, locus 1 (Drosophila)*	139.7
M0R9K7	*Olfactory receptor*	75.4

D4AC32	*Olfactory receptor*	54.98
Q9JKR5	*Palmitoyltransferase ZDHHC2*	70.4
P35704	*Peroxiredoxin-2*	67.52
P25113	*Phosphoglycerate mutase 1*	95.22
Q6AYU5	*Poly(RC) binding protein 2*	56.18
F1LVG9	*Protein Alms1*	28.31
D4ABZ7	*Protein Arhgap22*	59.41
R9PY08	*Protein disulfide-isomerase A5*	76.45
F8WG83	*Protein Fam71e2*	29.55
F1M0R6	*Protein Fancf*	113.84
B2RYK0	*Protein Glcci1*	42.97
Q62669	*Protein Hbb-b1*	89.28
D3ZEI4	*Protein Hepacam*	35.67
D3Z895	*Protein Layn*	62.56
F7E4S0	*Protein LOC100362110 (Fragment)*	43.65
M3ZCQ3	*Protein LOC100910765*	89.28
M0R647	*Protein LOC100911144*	92.93
D3ZYH3	*Protein LOC100912928*	38.94
M0R6S6	*Protein LOC689679 (Fragment)*	94.52

F1M4C6	*Protein LOC690568 (Fragment)*	127.11
D4A3B6	*Protein Mtcp1*	65.94
E9PSV7	*Protein Nr6a1*	50.15
M0R6P7	*Protein Nr6a1 (Fragment)*	51.79
D4A212	*Protein Nxnl2*	124.01
D3ZW44	*Protein Ralyl*	151.75
Q66HG8	*Protein Red*	73.66
G3V7F6	*Protein RGD1561590*	85.6
D4ACT0	*Protein Rmi2*	57.23
F1M9D3	*Protein S1pr3*	34.87
B0BN66	*Protein Sapcd2*	81.8
D3ZZX9	*Protein Sfxn4*	43.39
F1LTK4	*Protein Srrd (Fragment)*	28.31
D4A9L2	*Protein Srsf1*	93.92
D4A1I4	*Protein Sys1*	54.68
B2GV90	*Protein Tmem219*	105.31
D4A424	*Protein Tmem52b*	54.47
Q304F3	*Protein Tnnc2*	565.44
F7F1N8	*Protein Usp39*	63.16

B1WBW3	*Protein Zfp579*	52.54
D3ZBV5	*Protein Zfp7*	358.12
Q6IE06	*Putative inactive serine protease 58*	110.91
Q27W01	*RNA-binding protein 8A*	168.86
Q6AXX1	*Schlafen-like protein 1*	57.85
Q62985	*SH2B adapter protein 1*	43.17
Q9JK00	*Sodium channel subunit beta-3*	64.66
Q4V8C6	*Suppressor of fused homolog*	103.92
Q5FVR8	*TBCC domain-containing protein 1*	30.9
Q9WTQ6	*Tribbles homolog 3*	100.94
A0JPQ4	*Tripartite motif-containing protein 72*	68.97
B5DF24	*Uridine-cytidine kinase*	53.41

Table S8. Unique proteins found in EDL muscle samples from DEG rats (present only in samples from animals exposed to 1 mg/kg/day of Dexamethasone for 10 days).

Accession number	***Protein name***	***PLGS Score***
P62832	*60S ribosomal protein L23*	236.14
Q7TMC7	*Ab2-417*	97.5
Q7TPI7	*Ac2-269*	84.94
D3ZCH1	*Ankyrin repeat domain 12 (Predicted), isoform CRA_b*	89.97
Q5M7T0	*Asparagine-linked glycosylation 3, alpha-1,3-mannosyltransferase homolog (S. cerevisiae)*	80.68
Q5U2U8	*Bcl2-associated athanogene 3*	61.87
Q32WR5	*Biogenesis of lysosome-related organelles complex-1 subunit 2*	190.17
P55280	*Cadherin-6*	55.77
F1LR80	*CaM kinase-like vesicle-associated protein*	102.99
D3ZB14	*CASP2 and RIPK1 domain containing adaptor with death domain, isoform CRA_a*	97.9
D4A346	*Centaurin, beta 5 (Predicted), isoform CRA_a*	65.32
Q63430	*Cold shock domain-containing protein C2*	100.12
Q920J3	*Coronin-6*	91.63
P13107	*Cytochrome P450 2B3*	65.47
Q6AXY8	*Dehydrogenase/reductase (SDR family) member 1*	137.77

Q5I0I2	*E3 ubiquitin-protein ligase MARCH2*	72.03
P13803	*Electron transfer flavoprotein subunit alpha, mitochondrial*	56.06
P97553	*Ephrin-A1*	97.66
P07687	*Epoxide hydrolase 1*	53.08
A9UMW3	*Erythroid associated factor*	93
O54924	*Exocyst complex component 8*	69.63
P70552	*GTP cyclohydrolase 1 feedback regulatory protein*	119.83
P63018	*Heat shock cognate 71 kDa protein*	72.93
Q0IKU7	*Influenza virus NS1A binding protein (Predicted), isoform CRA_a*	80.8
P17105	*Inositol-trisphosphate 3-kinase A*	71.2
Q5BKB0	*Interleukin 1 beta*	80.6
P16598	*Interleukin-1 alpha*	69.44
Q63264	*Interleukin-1 beta*	80.6
P43303	*Interleukin-1 receptor type 2*	58.89
P27471	*Killer cell lectin-like receptor subfamily B member 1A*	90.29
A1A5P5	*LisH domain-containing protein ARMC9*	77.1
Q66H35	*Lung adenoma susceptibility protein 2 homolog*	118.24
Q5XIU9	*Membrane-associated progesterone receptor component 2*	76.88
Q62625	*Microtubule-associated proteins 1A/1B light chain 3B*	56.77

Q6AYN8	*Minichromosome maintenance deficient 7 (S. cerevisiae)*	55
O70540	*Mucosal addressin cell adhesion molecule 1*	79.82
D3ZNE7	*Olfactory receptor*	55.87
D3ZFW9	*Olfactory receptor*	113.03
M0RBN5	*Olfactory receptor*	48.82
M0R9J3	*Olfactory receptor*	53.49
G3V7E0	*Orthopedia homolog (Drosophila), isoform CRA_a*	96.52
P45479	*Palmitoyl-protein thioesterase 1*	64.29
Q56A20	*Phosphoinositide-3-kinase-interacting protein 1*	67.87
Q3SWT0	*Platelet endothelial cell adhesion molecule*	69.37
G3V754	*POU domain protein*	69.25
F1M2W7	*Protease inhibitor 15 (Predicted)*	73.39
F1M8K7	*Protein Alg3*	80.68
D3ZGR5	*Protein Ankrd12*	84.56
F1M332	*Protein Arhgap28*	53.09
F7F1L0	*Protein Armc6*	77.76
D4A3H6	*Protein B3gnt4*	49.81
M0RBC7	*Protein Bco2 (Fragment)*	71.47
E9PTV4	*Protein Bms1*	166.09

D3ZTC4	*Protein Ccdc88b*	54.15
D3ZCZ3	*Protein Cys1*	72.16
D3ZDW9	*Protein Dok7*	66.63
D4A1P0	*Protein Fam216b*	105.49
D4A5T7	*Protein Ift52*	58.32
D3ZGR4	*Protein Kansl1l*	57.38
F1M635	*Protein Lbh (Fragment)*	97.02
D3ZK57	*Protein LOC100363638*	119.63
Q7TQ75	*Protein LOC100363716*	56.77
M0R4Y2	*Protein LOC100910401 (Fragment)*	90.08
M0RDY4	*Protein LOC316124*	64.3
F1LZI1	*Protein LOC680121*	72.93
D3ZS48	*Protein Lsm14b*	97.97
D3ZD50	*Protein Marveld2*	54.03
D4A9W8	*Protein Mbd4*	53.2
D4A4U3	*Protein Mdp1*	83.85
F1MAG2	*Protein Npas2*	63.12
D3ZNC0	*Protein Otud1*	54.53
E9PT60	*Protein Pcdhga9*	98.92

F1LVC3	*Protein Rab6b*	64.39
M0R9M2	*Protein RGD1561143 (Fragment)*	71.46
D3ZF25	*Protein RGD1561909*	61.32
D3ZPA7	*Protein Scara3*	56.84
F1MAA9	*Protein Serpinb3*	57.11
B1H291	*Protein Shcbp1l*	123.47
G3V970	*Protein Six1*	56.02
D3ZH43	*Protein Slc38a10*	71.94
D4ABT5	*Protein Smarce1l*	90.66
Q2YDU8	*Protein spinster homolog 1*	73.19
D4A712	*Protein Tcf25*	65.17
D4A8B5	*Protein Tex26*	56.5
F1MAB9	*Protein Tpd52*	147.36
D4ADY0	*Protein Vsig2*	58.63
D4A1V0	*Protein Zfp213*	56.84
Q5FVJ1	*Protein Zfp956*	67.89
Q99M75	*Reticulon-4 receptor*	61.95
P18298	*S-adenosylmethionine synthase isoform type-2*	70.73
F1LRB8	*S-adenosylmethionine synthase*	65.54

P09005	*Serine protease inhibitor 2.1 (Fragment)*	69.24
Q4FZX7	*Signal recognition particle receptor subunit beta*	82.79
A2RUW1	*Toll-interacting protein*	97.84
Q5HZE2	*Transmembrane protein 120A*	88.6
Q5U4F4	*Transmembrane protein 135*	65.43
Q5FVQ9	*Tubulin-specific chaperone E*	81.92
Q5PQX0	*UDP-glucuronic acid decarboxylase 1*	79.65
P53813	*Vitamin K-dependent protein S*	55.99
Q6PCU2	*V-type proton ATPase subunit E 1*	116.07
A1L1J6	*Zinc finger protein 652*	61.59

Table S9. Unique proteins found in EDL muscle samples from DEHG rats (animals exposed to 1 mg/kg/day of Dexamethasone and 300 mg/kg/day of HMB for 10 days).

Accession number	***Protein name***	***PLGS Score***
Q5MYT9	*2'-5'-oligoadenylate synthase-like protein 2*	45.76
Q62904	*3-keto-steroid reductase*	60.18
P62845	*40S ribosomal protein S15*	98.35
P17074	*40S ribosomal protein S19*	54.76
Q52KS1	*6-phosphofructokinase*	53.01
A2VD14	*Abcf2 protein*	62.5
D4A7W1	*Abhydrolase domain containing 2 (Predicted)*	45.29
E9PSQ1	*Alpha-amylase*	58.3
D4AEH9	*Amylo-1, 6-glucosidase, 4-alpha-glucanotransferase (Glycogen debranching enzyme, glycogen storage disease type III) (Predicted), isoform CRA_a*	51.34
F1M8D5	*Amyotrophic lateral sclerosis 2 (Juvenile) chromosome region, candidate 13 (Predicted)*	123.05
D3ZQX4	*Anaphase-promoting complex subunit 15*	105.57
P53678	*AP-3 complex subunit mu-2*	68.37
V9GZ82	*AP-3 complex subunit mu-2*	63.67
Q8VDA6	*Arginine vasopressin-induced protein 1*	78.13

M0R3Y2	*Arylacetamide deacetylase*	107.84
Q06647	*ATP synthase subunit O, mitochondrial*	89.38
B2GV35	*B3gat3 protein*	57.94
Q9JKC1	*Butyrylcholinesterase*	76.65
Q8CFN2	*Cell division control protein 42 homolog*	123.67
Q9WTV1	*Chitinase-3-like protein 1*	55.26
G3V936	*Citrate synthase*	54.46
P02466	*Collagen alpha-2(I) chain*	62.03
Q6TUH9	*Corticosteroid 11-beta-dehydrogenase isozyme 1*	83.3
P09605	*Creatine kinase S-type, mitochondrial*	69.69
D3ZML3	*Cyclin-dependent kinase 11B (Fragment)*	77.14
Q62871	*Cytoplasmic dynein 1 intermediate chain 2*	56.71
Q62696	*Disks large homolog 1*	53.9
P31016	*Disks large homolog 4*	46.81
Q6IM78	*DNA topoisomerase I, mitochondrial*	52.97
Q5YKV6	*DnaJ (Hsp40) related, subfamily B, member 13*	56.57
D4A098	*Doublesex and mab-3 related transcription factor 2 (Predicted)*	111.82
F1LN35	*Dystrophin*	47.72
Q5XI50	*E3 ubiquitin-protein ligase MARCH7*	58.34

D3ZTX5	*EF hand domain family, member B (Predicted), isoform CRA_b*	45.05
P62630	*Elongation factor 1-alpha 1*	59.44
P62632	*Elongation factor 1-alpha 2*	50.12
D3ZC03	*Enolase*	61.48
G3V7B8	*Excision repair cross-complementing rodent repair deficiency, complementation group 8 (Predicted), isoform CRA_f*	68.77
F1LYQ8	*FERM, RhoGEF and pleckstrin domain-containing protein 1*	53.51
Q9EST9	*Fibroblast growth factor 20*	125.15
P50609	*Fibromodulin*	53.27
Q9Z1N1	*Fructose-1,6-bisphosphatase isozyme 2*	73.27
Q5J2D6	*Gametogenetin-binding protein 1*	86.27
F1M1E8	*Glyceraldehyde-3-phosphate dehydrogenase (Fragment)*	48.71
P20781	*Glycine receptor subunit beta*	45.13
P09811	*Glycogen phosphorylase, liver form*	80.17
Q04807	*Glycosylation-dependent cell adhesion molecule 1*	57.52
Q67EV2	*GP49B2*	91.53
A0JPN1	*H2.0-like homeobox protein*	79.73
D3ZXP3	*Histone H2A*	126.51
Q6LED0	*Histone H3.1*	52.54

D3ZFJ6	*Lactamase, beta (Predicted)*	65.16
Q9WVB7	*Lens epithelial cell protein LEP503*	175.81
Q5FVI3	*Leucine-rich repeat-containing protein 57*	69.99
B2GV98	*Lrch1 protein*	66.04
B0BN00	*Lrrc27 protein*	80.27
O88989	*Malate dehydrogenase, cytoplasmic*	50.57
P50282	*Matrix metalloproteinase-9*	53.27
Q6AYB1	*MHC class II antigen*	87.72
Q6P7D5	*Mid1-interacting protein 1*	186.95
D3ZQ33	*Mitogen activated protein kinase 10, isoform CRA_a*	84.89
D3ZA38	*Myosin binding protein C, fast-type (Predicted)*	59.98
O54697	*N-acetylated-alpha-linked acidic dipeptidase-like protein*	61.13
Q5RJQ4	*NAD-dependent protein deacetylase sirtuin-2*	75.27
P19234	*NADH dehydrogenase [ubiquinone] flavoprotein 2, mitochondrial*	82.77
Q4V8K3	*N-alpha-acetyltransferase 11*	118.7
Q8CGU6	*Nicastrin*	52.11
P70503	*Nuclear receptor subfamily 0 group B member 1*	67.01
Q1RP77	*Nucleolar protein 16*	244.26
D3Z9Y6	*Olfactory receptor*	67.75

D3ZIT1	*Olfactory receptor*	48.4
M0RA08	*Perilipin*	55.94
Q642G4	*Peroxisomal membrane protein PEX14*	48.95
Q6MGC9	*PHD finger protein 1*	75.91
Q5U317	*Pre-mRNA 3'-end-processing factor FIP1*	57.69
O08628	*Procollagen C-endopeptidase enhancer 1*	75.69
D3ZNT5	*Procollagen, type IX, alpha 2 (Predicted)*	49.8
P43253	*Prostacyclin receptor*	100.18
F1LZU7	*Protein Apbb2 (Fragment)*	77.51
Q6P691	*Protein C2cd2*	103.8
D3ZZ65	*Protein Casp14*	66.06
D3Z8X7	*Protein Cep104*	63.42
F1MAG0	*Protein Dfna5 (Fragment)*	94.4
D3ZD46	*Protein Dhx58*	55.87
Q63081	*Protein disulfide-isomerase A6*	60.61
D4AE51	*Protein Ecscr*	110.05
D3ZJY0	*Protein Fam13b*	45.11
B0BN67	*Protein Fam181b*	64.29
D3ZTZ0	*Protein Fam184b*	56.13

D3ZW88	*Protein Frmpd1*	45.36
M0R8F9	*Protein Gm17555 (Fragment)*	67.33
D4ACU6	*Protein Gpr123 (Fragment)*	55.74
G3V6Z0	*Protein Hoxa11*	60.59
D4A435	*Protein Icam5*	49.58
M0RB74	*Protein Ipo5*	58.15
M0RCW8	*Protein Iqcc*	71.58
D3ZJ09	*Protein Kansl1*	52.9
D3ZG98	*Protein Lancl3*	53.77
D4A6G6	*Protein LOC100362339*	54.76
F1LYU9	*Protein LOC100363064*	85.23
M0R9M1	*Protein LOC100909686*	48.4
D3ZM03	*Protein LOC100912478*	192.54
D3ZVY0	*Protein LOC102550988*	75.72
D4A8Z5	*Protein LOC102553119*	62.43
F1LV00	*Protein LOC678817 (Fragment)*	75.79
M0RAT3	*Protein LOC685048*	160.06
D3ZDV5	*Protein Lrrc27*	80.27
A0A096MJZ6	*Protein Mef2c*	129.53

D3ZEX4	*Protein Olr1335 (Fragment)*	51.72
D4ACT8	*Protein Pcdhga3*	51.69
M0R485	*Protein Pglyrp2*	129.01
D3ZPS3	*Protein Pglyrp2*	129.01
D3Z9K4	*Protein Plekho2*	50.16
D3ZVQ3	*Protein Porcn*	69.29
D4A1A5	*Protein Ppp2r5c*	77.82
D3ZS72	*Protein Prex1 (Fragment)*	49.21
Q5BK00	*Protein RGD1305455*	71.57
B5DFL7	*Protein Sema3c*	58.48
F1LS93	*Protein Sh3bp2*	70.73
D3ZUN1	*Protein Slc17a9*	64.6
D4A8R5	*Protein Slc39a12*	59.08
F1LR22	*Protein Smyd3 (Fragment)*	108.06
D4A3Z3	*Protein Tbc1d24*	56.33
B4F7D5	*Protein Tmem181*	58.44
F1M5H6	*Protein Tp53bp2 (Fragment)*	46.62
Q5FVQ1	*Protein Traf3ip3*	69.18
D3ZDJ6	*Protein Vstm2l*	82.17

B1WC22	*Protein Zfp131*	64.62
D3ZQ68	*Protein Zfp263*	56.84
D3ZDC7	*Protein Zfyve9*	59.63
D4A5A9	*Protein Zswim8 (Fragment)*	85.98
P35248	*Pulmonary surfactant-associated protein D*	135.22
G3V8L1	*PYD and CARD domain containing*	119.64
P29826	*Rano class II histocompatibility antigen, B-1 beta chain*	87.72
P51156	*Ras-related protein Rab-26*	58.53
P62824	*Ras-related protein Rab-3C*	91.82
F1M520	*RCG28256 (Fragment)*	105.63
Q5PPN2	*Ribonuclease P protein subunit p25*	49.3
Q68FQ7	*RNA polymerase II-associated protein 3*	52.96
Q62991	*Sec1 family domain-containing protein 1*	51.2
D3ZHP7	*Serine/threonine-protein kinase ULK3*	62.47
P02770	*Serum albumin*	70.75
Q925Q9	*SH3 domain-containing kinase-binding protein 1*	56.91
P0DJJ3	*SH3-containing GRB2-like protein 3-interacting protein 1*	47.66
B5DEL3	*Tetratricopeptide repeat protein 17*	48.72
P41224	*Thyrotroph embryonic factor*	72.94

D3ZHV6	*Transmembrane channel-like protein*	49.54
Q7TQ79	*Transmembrane protein 170B*	71
Q5PQJ7	*Tubulin-specific chaperone cofactor E-like protein*	74.31
D3Z8K5	*Ubiquitin carboxyl-terminal hydrolase*	46.12
Q498S6	*Zinc finger CCHC domain-containing protein 2*	37.01
D3ZEI3	*Zinc finger protein 821*	70.74
Q3MHS2	*Zinc finger protein 830*	72

Table S10. Proteins identified in the EDL muscle of animals with differential expression in the DEG group compared to the PEG group (upregulated or downregulated).

Accession number	***Protein name***	***PLGS Score***	**Protein Expression Ratio**<br><br>**DEG:PEG**
P00330	*Alcohol dehydrogenase 1*	896.4	0.778800783
P00564	*Creatine kinase M-type*	743.86	0.818730751
O88752	*Epsilon 1 globin*	3045.01	0.755783741
P05065	*Fructose-bisphosphate aldolase A*	1768.39	0.895834136
P02091	*Hemoglobin subunit beta-1*	4494.34	0.860707971
P11517	*Hemoglobin subunit beta-2*	3735.43	0.810584251
Q29RW1	*Myosin-4*	667.21	0.826959136
P02564	*Myosin-7*	695.92	0.93239382
Q64578	*Sarcoplasmic/endoplasmic reticulum calcium ATPase 1*	30.44	0.71177032
P04466	*Myosin regulatory light chain 2, skeletal muscle isoform*	641.24	0.826959136
G3V9M3	*Protein Wdr47*	66.19	0.852143792
P16409	*Myosin light chain 3*	259.65	0.522045789
Q8R4I6	*Actinin alpha 3, isoform CRA_a*	174.88	0.826959136
P02563	*Myosin-6*	666.63	0.941764535

Q5XIG1	*Ldb3 protein*	85.04	1.209249595
D3ZCV0	*Protein Actn2*	88.36	1.309964465
Q4V7E8	*Leucine-rich repeat flightless-interacting protein 2*	50.77	1.716006899
G3V7K1	*Myomesin 2*	30.85	1.246076729
A0A096MJ01	*Protein Ldb3*	85.04	1.258600015
M0RCX5	*Protein LOC102554588 (Fragment)*	86.39	2.559981412
D3ZML2	*Serine/threonine-protein kinase BRSK2*	36.73	2.386910865
P58775	*Tropomyosin beta chain*	477.95	1.16183425
P10719	*ATP synthase subunit beta, mitochondrial*	276.84	1.271249144
P02454	*Collagen alpha-1(I) chain*	86.86	1.377127754
E9PSK9	*Protein Ctsql2*	46.53	1.896480852
F1MAC2	*Protein Krt78*	61.24	1.858928051
Q91XN6	*Tropomyosin 1, alpha, isoform CRA_h*	332.36	1.221402762

Ratio values below 1 represent downregulated proteins, while ratio values above 1 correspond to upregulated proteins. The identified proteins are divided into downregulated proteins at the top of the table and upregulated proteins at the bottom.

Table S11. Proteins identified in the EDL muscle of animals with differential expression in the DEHG group compared to the PEG group (upregulated or downregulated).

Accession number	***Protein name***	***PLGS Score***	**Protein Expression Ratio**<br><br>**DEHG:PEG**
P68136	*Actin, alpha skeletal muscle*	10253.84	0.951229
P00330	*Alcohol dehydrogenase 1*	896.4	0.786628
P00564	*Creatine kinase M-type*	743.86	0.802519
Q29RW1	*Myosin-4*	667.21	0.852144
Q64578	*Sarcoplasmic/endoplasmic reticulum calcium ATPase 1*	30.44	0.71177
Q6DKG0	*N-alpha-acetyltransferase 35, NatC auxiliary subunit*	27.62	0.382893
P16409	*Myosin light chain 3*	259.65	0.444858
G3V8B0	*Myosin-7*	695.92	0.941765
P02563	*Myosin-6*	666.63	0.941765
E9PSX6	*Calcium-transporting ATPase*	139.76	0.637628
P11507	*Sarcoplasmic/endoplasmic reticulum calcium ATPase 2*	139.76	0.631284
G3V6E1	*Myosin heavy chain 2*	735.59	1.061837
P86252	*Transcriptional activator protein Pur-alpha (Fragments)*	235.47	3.126768
P09739	*Troponin T, fast skeletal muscle*	330.61	1.197217

D4A7U8	*Protein Myoz1*	245.29	1.349859
F1LP05	*ATP synthase subunit alpha*	216.16	1.197217
Q4V7E8	*Leucine-rich repeat flightless-interacting protein 2*	50.77	1.616074
F1M513	*Protein Rufy4*	32.55	1.616074
F1LT99	*SH3-containing GRB2-like protein 3-interacting protein 1 (Fragment)*	132.99	2.58571
G3V7K1	*Myomesin 2*	30.85	1.390968
P02091	*Hemoglobin subunit beta-1*	4494.34	1.197217
P11517	*Hemoglobin subunit beta-2*	3735.43	1.116278
P01946	*Hemoglobin subunit alpha-1/2*	3580.13	1.447735
O88752	*Epsilon 1 globin*	3045.01	1.116278
P02600	*Myosin light chain 1/3, skeletal muscle isoform*	2528.3	1.105171
F1LRV9	*Protein Myh2*	1023.86	1.061837
F1M3U4	*Gp_dh_N domain-containing protein*	671.43	1.246077
P10719	*ATP synthase subunit beta, mitochondrial*	276.84	1.29693
G3V8V3	*Alpha-1,4 glucan phosphorylase*	223.2	1.185305
P00489	*Glycogen phosphorylase, muscle form*	168.47	1.173511
P09812	*Glycogen phosphorylase, muscle form*	161.85	1.173511
O88751	*Calcium-binding protein 1*	109.9	1.094174
D3ZCV0	*Protein Actn2*	88.36	1.447735

P02454	*Collagen alpha-1(I) chain*	86.86	1.840431
Q5XIG1	*Ldb3 protein*	85.04	1.462285
A0A096MKD4	*Protein Ldb3*	85.04	1.476981
M0RCJ8	*Protein Krt78*	74.13	1.733253
E9PSN4	*Protein Zc3h13*	53.98	1.768267

Ratio values below 1 represent downregulated proteins, whereas ratio values above 1 correspond to upregulated proteins. The identified proteins are divided into downregulated at the top of the table and upregulated at the bottom of the table.

Table S12. Proteins identified in the EDL muscle of animals with differential expression in the DEHG group compared to the DEG group (upregulated or downregulated).

Accession number	***Protein name***	***PLGS Score***	**Protein Expression Ratio DEHG:DEG**
Q68FY0	*Cytochrome b-c1 complex subunit 1, mitochondrial*	117.28	0.096327646
F1MAC2	*Protein Krt78*	61.24	0.554327299
D4ABX0	*Protein Dcaf12l1*	121.77	0.21438111
Q91XN6	*Tropomyosin 1, alpha, isoform CRA_h*	332.36	0.869358235
P68035	*Actin, alpha cardiac muscle 1*	7076.85	0.970445534
P68136	*Actin, alpha skeletal muscle*	10253.84	0.970445534
M9MMM8	*Serine/threonine-protein kinase BRSK2 (Fragment)*	36.73	0.357006971
P62738	*Actin, aortic smooth muscle*	6899.54	0.980198674
Q6MG75	*Protein Nelfe*	79.77	3.896193358
P00489	*Glycogen phosphorylase, muscle form*	168.47	1.1502738
P04466	*Myosin regulatory light chain 2, skeletal muscle isoform*	641.24	1.173510867
E9PSN4	*Protein Zc3h13*	53.98	1.377127754
A0A096MJ11	*Gasdermin domain containing 1 (Predicted), isoform CRA_a*	95.76	1.173510867
G3V6E1	*Myosin heavy chain 2*	735.59	1.051271097

P02600	*Myosin light chain 1/3, skeletal muscle isoform*	2528.3	1.083287066
A0A096MJ01	*Protein Ldb3*	85.04	1.185304853
Q8R4I6	*Actinin alpha 3, isoform CRA_a*	174.88	1.209249595
G3V8V3	*Alpha-1,4 glucan phosphorylase*	223.2	1.246076729
P02454	*Collagen alpha-1(I) chain*	86.86	1.336427477
O88752	*Epsilon 1 globin*	3045.01	1.476980773
P05065	*Fructose-bisphosphate aldolase A*	1768.39	1.094174288
F1M3U4	*Gp_dh_N domain-containing protein*	671.43	1.323129814
P01946	*Hemoglobin subunit alpha-1/2*	3580.13	1.349858824
P02091	*Hemoglobin subunit beta-1*	4494.34	1.377127754
P11517	*Hemoglobin subunit beta-2*	3735.43	1.377127754
Q5HZD9	*LOC100125377 protein*	149.19	1.840431425
F1LRV9	*Protein Myh2*	1023.86	1.083287066

Ratio values below 1 represent downregulated proteins, whereas ratio values above 1 correspond to upregulated proteins. The identified proteins are divided into downregulated at the top of the table and upregulated at the bottom of the table.

Table S13. Unique proteins found in liver samples from PEG rats (animals exposed only to placebo treatments containing saline solution)

Accession number	***Protein name***	***PLGS Score***
Q2MHD9	*17beta-hydroxysteroid dehydrogenase*	85.28
A9CMA9	*2-amino-3-carboxymuconate-6-semialdehyde decarboxylase (Fragment)*	118.91
D4A5C3	*3-hydroxy-3-methylglutaryl-CoA lyase_ cytoplasmic*	64.44
P62909	*40S ribosomal protein S3*	102.48
D4A2K1	*4-hydroxy-2-oxoglutarate aldolase 1*	76.77
Q7TMA9	*Aa1249*	115.18
Q7TP71	*Ab2-051*	86.97
G3V796	*Acetyl-Coenzyme A dehydrogenase_ medium chain*	119.14
M0R6F2	*Acyl-CoA dehydrogenase family_ member 10*	72.62
F1LSP2	*Acyl-CoA dehydrogenase family_ member 10*	78.59
F1LNW3	*Acyl-coenzyme A oxidase*	60.1
A0A0G2K125	*Acyl-coenzyme A synthetase ACSM2_ mitochondrial*	82.13
A0A0G2K7Z3	*Acyl-coenzyme A thioesterase 1*	62.04
O88267	*Acyl-coenzyme A thioesterase 1*	56.84
A0A0G2K6X9	*Adenosine kinase*	68.2

P10760	*Adenosylhomocysteinase*	190.26
Q05962	*ADP/ATP translocase 1*	74.43
Q66HA6	*ADP-ribosylation factor-like protein 8B*	69.43
A0A0G2K1Y9	*AF4/FMR2 family_ member 2*	53.77
Q38PG3	*AHNAK 1 (Fragment)*	54.24
Q5XI95	*Alcohol dehydrogenase 6*	61.74
P30839	*Aldehyde dehydrogenase family 3 member A2*	105.68
Q5I0M4	*Aldo-keto reductase family 1_ member C12*	85.28
D3ZPY8	*Aldo-keto reductase family 1_ member C13*	75.41
D3ZEL2	*Aldo-keto reductase family 1_ member C19*	74.96
Q7TP08	*Alpha-methylacyl-CoA racemase*	68.56
O09178	*AMP deaminase 3*	56.5
F1LYE2	*Androglobin*	59.13
O35462	*Angiopoietin-2*	132.25
Q7TMA5	*Apolipoprotein B-100*	55.81
Q8CGU4	*Arf-GAP with GTPase_ ANK repeat and PH domain-containing protein 2*	70.2
Q78E60	*Aryl hydrocarbon receptor nuclear translocator 2*	57.02
P49088	*Asparagine synthetase [glutamine-hydrolyzing]*	93.6
P13221	*Aspartate aminotransferase_ cytoplasmic*	87.46

O35815	*Ataxin-3*	54.19
D3ZLV5	*AT-hook transcription factor*	49.17
G3V7W3	*Atrophin-1*	51.76
B1WBV1	*Axin interactor_ dorsalization-associated*	103.26
Q64642	*Brain-enriched membrane-associated protein tyrosine phosphatase (BEM)-2*	67.75
B5DFA3	*Bucs1 protein*	73.08
D3ZLU0	*C2 calcium-dependent domain-containing 4C*	49.39
D4A5K6	*CAAX prenyl protease*	65.8
Q8R490	*Cadherin 13*	50.07
A0A0G2JZZ3	*Cap-binding complex-dependent translation initiation factor*	78.1
G3V7J5	*Carboxylic ester hydrolase*	73.84
F1LQT4	*Carboxypeptidase N subunit 2*	62.86
F1M642	*CCR4-NOT transcription complex_ subunit 6-like*	61.78
P63182	*Cerebellin-1*	120.65
A0A0G2K240	*Cilia and flagella-associated protein 65*	94.29
Q32Q01	*Ckap5 protein (Fragment)*	56.36
D3ZL71	*Clarin 2*	146.61
B4F764	*Cleavage and polyadenylation specificity factor subunit 5*	60.28
M0RC65	*Cofilin 2*	96.49

D3Z8H9	*Coiled-coil domain-containing 103*	93.18
Q6TUH9	*Corticosteroid 11-beta-dehydrogenase isozyme 1*	105.49
P48199	*C-reactive protein*	115.18
A0A0G2K1N8	*Cysteine and glycine-rich protein 3*	62.88
Q64611	*Cysteine sulfinic acid decarboxylase*	213.67
Q5UAJ6	*Cytochrome c oxidase subunit 2*	60.55
P10633	*Cytochrome P450 2D1*	65.2
P12939	*Cytochrome P450 2D10*	77.21
Q64680	*Cytochrome P450 2D4*	58.78
P33274	*Cytochrome P450 4F1*	296.62
P51869	*Cytochrome P450 4F4*	69.74
F1M949	*Cytoskeleton-associated protein 5*	78.56
Q68FS4	*Cytosol aminopeptidase*	72.09
Q64592	*Delta3_ delta2-enoyl-CoA isomerase*	64.02
Q01205	*Dihydrolipoyllysine-residue succinyltransferase component of 2-oxoglutarate dehydrogenase complex_ mitochondrial*	108.89
Q63150	*Dihydropyrimidinase*	62.44
D0VYQ0	*DLST protein*	136.21
Q7M081	*DnaK-type molecular chaperone P72 (Fragments)*	97.5

D3Z939	*Dpy-19-like 4*	87.69
D4ADU7	*EEF1A lysine methyltransferase 4*	128.47
B5DF91	*ELAV-like protein 1*	59.35
A0A0G2K2R2	*Enoyl-CoA delta isomerase 1_ mitochondrial*	167.71
P07687	*Epoxide hydrolase 1*	92.81
Q3B8Q2	*Eukaryotic initiation factor 4A-III*	58.55
D3ZLE0	*Exonuclease 5*	77.87
P31977	*Ezrin*	76.41
D4A176	*Family with sequence similarity 71_ member E1*	123.45
D3ZW92	*Family with sequence similarity 78_ member A*	106.51
P12785	*Fatty acid synthase*	61.76
D3ZFK8	*FERM_ ARH/RhoGEF and pleckstrin domain protein 2*	57.72
F1LTM1	*Ferritin*	88.77
A0A096P6L8	*Fibronectin*	111.1
M0R6E1	*Forkhead box L1*	67.75
P19112	*Fructose-1_6-bisphosphatase 1*	100.99
Q498R5	*FSA_C domain-containing protein*	92.08
Q9JI49	*Fudenine*	52.36
Q99521	*Gastrin-binding protein (Fragment)*	84.1

Q8CJG5	*Gene*	86.22
D4A275	*Glucoside xylosyltransferase 2*	92.77
P04041	*Glutathione peroxidase 1*	213.58
Q9Z1B2	*Glutathione S-transferase Mu 5*	70.48
G3V8H3	*Glutathione S-transferase*	61.55
B0BN47	*Glutathione S-transferase*	61.9
B6DYQ3	*Glutathione S-transferase*	112.78
O35077	*Glycerol-3-phosphate dehydrogenase [NAD(+)]_ cytoplasmic*	161.98
M0RDH0	*Glycine N-methyltransferase*	176.98
P09811	*Glycogen phosphorylase_ liver form*	88.74
Q4AEG0	*Glycoprotein 9*	97
P16261	*Graves disease carrier protein (Fragment)*	117.4
P14659	*Heat shock-related 70 kDa protein 2*	144.65
P62804	*Histone H4*	261.35
P97519	*Hydroxymethylglutaryl-CoA lyase_ mitochondrial*	70.39
D4A4N9	*Hypothetical protein LOC685849*	80.95
A0A0G2K4V5	*Integrin beta*	51.22
D3ZN51	*Integrin subunit alpha 9*	79.09
D3ZIM0	*Interleukin 17 receptor C*	67.92

D4A7K4	*Interleukin-1 receptor-associated kinase 4*
P20607	*Interleukin-6*
P41562	*Isocitrate dehydrogenase [NADP] cytoplasmic*
Q5BJS8	*Itpr1 protein (Fragment)*
A0A0G2JXJ9	*Keratin_ type I cytoskeletal 14*
Q63279	*Keratin_ type I cytoskeletal 19*
D3ZVH2	*KH type-2 domain-containing protein*
A0A0G2K857	*Kinesin-like protein*
Q64602	*Kynurenine/alpha-aminoadipate aminotransferase_ mitochondrial*
Q63288	*L1 retroposon_ ORF2 mRNA (Fragment)*
B0BNG3	*Lectin_ mannose-binding 2*
Q6P2A4	*Leucine-rich repeat LGI family_ member 4*
Q5M9H1	*Leucine-rich repeat-containing protein 41*
D3ZAG6	*Limb and CNS-expressed 1*
Q64573	*Liver carboxylesterase 4*
Q7TNW9	*Liver regeneration-related protein LRRG07*
P42123	*L-lactate dehydrogenase B chain*
H9N9H4	*L-lactate dehydrogenase C chain*
Q6AYX2	*L-lactate dehydrogenase*

Q5BK04	*LOC363306 protein (Fragment)*	63.89
Q5I0H7	*LOC682097 protein (Fragment)*	72.76
Q6QI78	*LRRG00130*	118.09
Q6QI76	*LRRG00132*	105.49
Q6QI75	*LRRG00133*	77.76
Q6QI73	*LRRG00135*	92.14
Q6TXF0	*LRRGT00049*	32.64
Q6TUG5	*LRRGT00079*	32.64
Q6TUF9	*LRRGT00085*	119.61
Q6TUF6	*LRRGT00088*	105.49
Q6QI65	*LRRGT00143*	86.97
Q6QI58	*LRRGT00150*	70.83
Q6QI48	*LRRGT00160*	50.92
Q6QI17	*LRRGT00191*	105.49
Q6QI12	*LRRGT00196*	105.49
Q5FVQ4	*Malectin*	102.69
M0R7B6	*Mediator complex subunit 12-like (Fragment)*	70.14
P08503	*Medium-chain specific acyl-CoA dehydrogenase_ mitochondrial*	119.14
D3ZNC9	*Membrane associated ring-CH-type finger 4*	108.69

A0A0H2UHK2	*Membrane-associated progesterone receptor component 1*	70.92
P70580	*Membrane-associated progesterone receptor component 1*	70.92
A0A0G2JV93	*Metabotropic glutamate receptor 3*	52.12
Q562C4	*Methyltransferase-like protein 7B*	142.23
A0A0G2K7V7	*MHC class I polypeptide-related sequence B*	113.47
Q5UT99	*MHC class II antigen*	115.61
Q60I19	*MHC class I-like located near the LRC_ 2*	113.47
A0A0G2K7P7	*Mitochondrial carrier 2*	87.21
D3ZPN5	*Mitochondrial poly(A) polymerase*	82.11
A0A096MK24	*MORC family CW-type zinc finger 4*	80.47
A0A0G2K484	*Myosin heavy chain 1*	32.91
F1LMU0	*Myosin heavy chain 2*	32.91
Q9JLT0	*Myosin-10*	62.03
A0A0G2K6S9	*Myosin-11*	49.71
Q29RW1	*Myosin-4*	32.91
Q769K2	*N-acyl-phosphatidylethanolamine-hydrolyzing phospholipase D*	102.96
F1LNP8	*Nectin cell adhesion molecule 1*	96.74
Q6MG75	*Negative elongation factor complex member E*	114.89
Q8K5B7	*Neuron-specific class III beta-tubulin (Fragment)*	205.28

Q62707	*Nonmuscle myosin heavy chain-B (Fragment)*	57.69
F1LQ55	*Non-specific lipid-transfer protein*	81.87
F1LP37	*Nucleoredoxin-like 1*	73.74
Q05982	*Nucleoside diphosphate kinase A*	67.35
P19804	*Nucleoside diphosphate kinase B*	181.16
P97692	*ORF2 (Fragment)*	79.09
Q63305	*ORF3 protein*	105.49
Q5I0G7	*Pabpc4 protein (Fragment)*	74.21
H6X2X0	*Pentaxin*	115.18
P10111	*Peptidyl-prolyl cis-trans isomerase A*	219.23
A0A0G2JSS8	*Peroxiredoxin*	131.23
Q9R063	*Peroxiredoxin-5_ mitochondrial*	131.23
O35244	*Peroxiredoxin-6*	140.79
A1A5L2	*Pgm1 protein (Fragment)*	184.61
Q68G31	*Phenazine biosynthesis-like domain-containing protein*	122.55
Q99M64	*Phosphatidylinositol 4-kinase type 2-alpha*	60.89
Q5CZZ9	*Phosphatidylinositol 4-phosphate 5-kinase type-1 beta*	65.95
Q499Q4	*Phosphoglucomutase 1*	184.61
P38652	*Phosphoglucomutase-1*	177.18

D3Z9T8	*Phospholipid scramblase*	81.84
F1M1C9	*Phosphorylase b kinase regulatory subunit*	73.59
D4A9R2	*PKHD1-like 1*	59.39
A0A0G2QC04	*Plastin 1 OS=Rattus norvegicus OX=10116 GN=Pls1 PE=1 SV=1*	90.26
D4A779	*Pleckstrin homology like domain_ family B_ member 2*	71.26
B5DEY0	*Pls1 protein*	90.26
A0A1L1WKJ6	*Poliovirus receptor-related 1 (Fragment)*	96.74
D3ZSR2	*Polyadenylate-binding protein*	67.42
Q9Z0N7	*Potassium voltage-gated channel subfamily KQT member 1*	131.26
D4A3H1	*PRAME family member 8*	62
B0BNA0	*Prg4 protein (Fragment)*	100.7
Q9JJH2	*Probable G-protein coupled receptor 173 O*	87.81
O70417	*Prolactin-inducible protein homolog*	65.75
A0A0G2JWD0	*Prominin 1*	61.13
G3V6W6	*Proteasome 26S subunit_ ATPase 6*	123.81
P21670	*Proteasome subunit alpha type-4*	60.61
Q6DGG1	*Protein ABHD14B*	111.8
Q9ESN0	*Protein Niban 1*	92.52
Q66HG8	*Protein Red*	69.65

B0K032	*Protein tyrosine phosphatase 4A3*	89.54
Q32PW9	*Psmc6 protein (Fragment)*	123.81
Q9QZH5	*Putative phosphate/phosphoenolpyruvate translocator*	74.86
D4A7A8	*Rab7b_ member RAS oncogene family*	84.44
P29826	*Rano class II histocompatibility antigen_ B-1 beta chain*	115.61
Q99P74	*Ras-related protein Rab-27B*	92.32
B5LNR2	*RCG38817*	73.74
F1M1K0	*RCG39867_ isoform CRA_b*	48.4
D3ZY89	*RCG43696_ isoform CRA_b*	101.45
F1LRY5	*RCG45398*	99.8
B5DF29	*Replication factor C (Activator 1) 5*	218.48
Q80WD1	*Reticulon-4 receptor-like 2*	57.83
B5DEF6	*RGD1310159 protein*	72.62
Q3KRD9	*RGD1311595 protein (Fragment)*	92.76
Q32PX8	*Rho GTPase activating protein 9*	53.7
D4ADQ1	*Ribonucleotide reductase M2 B (TP53 inducible) (Predicted)*	56.17
D3Z8R4	*RNA-binding motif protein 25-like 1*	98.11
Q5M9F1	*RNA-binding protein 34*	84.27
B0LPN4	*Ryanodine receptor 2*	78.8

D4ADE6	*Sal-like 2 (Drosophila) (Predicted)*	70.37
Q64380	*Sarcosine dehydrogenase_ mitochondrial*	99.8
O88453	*Scaffold attachment factor B1*	62.88
Q99MS0	*SEC14-like protein 2*	70.55
F1LU12	*Similar to 20-alpha-hydroxysteroid dehydrogenase*	131.54
F1LRC7	*Similar to KIAA2026 protein*	69.52
A0A096MJT6	*Similar to RIKEN cDNA D630029K19*	98.24
B1H220	*Similar to Zbtb3 protein (Predicted)_ isoform CRA_a*	63.58
Q64093	*Sodium- and chloride-dependent transporter XTRP3*	73.62
D4A6Y6	*Solute carrier family 25 member 26*	94.93
D3ZB81	*Solute carrier family 25 member 31*	153.08
Q6J329	*Solute carrier family 25 member 47*	53.22
A0A0G2JTE6	*Solute carrier family 35 member E4*	88.95
P27867	*Sorbitol dehydrogenase*	59.73
Q6AXW5	*Sp56 protein*	112.74
A0A0G2K6M7	*Spalt-like transcription factor 2*	70.37
A0A0G2K677	*Spectrin beta chain*	69.88
Q9JKU6	*Spermatid perinuclear RNA-binding protein*	81.7
Q5BJU8	*Sptbn4 protein (Fragment)*	59.96

Q7TSW6	*Store-operated calcium entry regulator STIMATE*	73.19
B1H270	*Succinate--CoA ligase [GDP-forming] subunit beta_ mitochondrial*	61.28
M0RCF8	*Takusan domain-containing protein*	63.89
A0A0G2K8M4	*Takusan domain-containing protein*	63.89
A0A0G2K849	*TATA-box binding protein associated factor 4*	91.34
D4A3U2	*Tetraspanin*	124.8
Q5M8C7	*Thioredoxin domain containing 9*	88.77
Q8K581	*Thioredoxin domain-containing protein 9*	88.77
P06882	*Thyroglobulin*	70.28
D4A3S0	*Trafficking protein particle complex 6A*	93.78
Q07258	*Transforming growth factor beta-3 proprotein*	48.31
G3V6Q4	*Transporter*	73.62
P48500	*Triosephosphate isomerase*	86.85
Q2MHD8	*Truncated form of 17beta-hydroxysteroid dehydrogenase*	85.28
Q8K3P3	*Truncated KvLQT1-like protein (Fragment)*	133.69
Q6P9X7	*Txndc9 protein (Fragment)*	109.16
Q6IFV0	*Type I keratin KA11*	121.25
A0A0G2JUD3	*UDP glucuronosyltransferase 2 family_ polypeptide B10*	39.76
Q64550	*UDP-glucuronosyltransferase 1-1*	55.76

P09875	*UDP-glucuronosyltransferase 2B1*	39.76
P36511	*UDP-glucuronosyltransferase 2B15*	94.2
Q62789	*UDP-glucuronosyltransferase 2B7*	53.97
F1LTB8	*UDP-glucuronosyltransferase*	39.76
P70624	*UGT1A7*	74.67
Q68G32	*Ugt1a7c protein (Fragment)*	55.76
A0A0G2K121	*Unc-119 lipid-binding chaperone B*	102.69
Q04462	*Valine--tRNA ligase*	47.24
Q2YS36	*Voltage-dependent P/Q-type calcium channel alpha-1A subunit (Fragment)*	174.93
Q3KRC6	*Volume-regulated anion channel subunit LRRC8E*	97.9
Q5J3G7	*Vomeronasal type-1 receptor*	56.08
A0A0G2K5G9	*WD repeat-containing protein 55*	69.65
Q7TSY4	*Zona pellucida sperm-binding protein 3 receptor*	112.74

Table S14. Unique proteins found in liver samples from DEG rats (present only in samples from animals exposed to 1 mg/kg/day of Dexamethasone for 10 days)

**Accession number**	***Protein name***	***PLGS Score***
P14842	*5-hydroxytryptamine receptor 2A*	87.05
P62914	*60S ribosomal protein L11*	72.79
Q7TMA9	*Aa1249*	78.76
P17764	*Acetyl-CoA acetyltransferase_ mitochondrial*	107.84
Q4KLM5	*Activator of basal transcription 1*	87.36
Q6TXF3	*Acyl-CoA-binding protein*	359.69
P11030	*Acyl-CoA-binding protein*	350.93
F1LSD1	*Adenylate cyclase type 6*	73.05
Q5I0L1	*Akr1c1 protein (Fragment)*	81.65
D3ZXY4	*Aldehyde dehydrogenase 8 family_ member A1*	79.72
Q3MHS3	*Aldo-keto reductase family 1_ member C1*	81.65
D3ZPY8	*Aldo-keto reductase family 1_ member C13*	97.96
Q5D178	*Alpha 2 macroglobulin cardiac isoform (Fragment)*	129.22
P06238	*Alpha-2-macroglobulin*	114.54
P04764	*Alpha-enolase*	91.86

Q1P9T9	*Amyloid beta A4-like protein 1 (Fragment)*	484.96
F1LRS5	*Amyloid beta precursor-like protein 1*	484.96
D3ZG73	*ANK_REP_REGION domain-containing protein*	92.67
B2GV19	*Anks4b protein*	71.23
B5DF17	*Ankyrin repeat and SOCS box-containing 8*	73.06
M0RBX2	*Ankyrin repeat and sterile alpha motif domain-containing 4B*	83.57
B1WBV6	*Aplp1 protein*	484.96
P49088	*Asparagine synthetase [glutamine-hydrolyzing]*	50.61
D4AA26	*Atonal bHLH transcription factor 8*	149.24
Q06647	*ATP synthase subunit O_ mitochondrial*	70.2
Q9Z2G3	*ATP-citrate (Pro-S-)-lyase (Fragment)*	324.57
P16638	*ATP-citrate synthase*	337.37
A0A0G2K9U6	*Autophagy-related 16-like 1*	64.99
Q3HS84	*B and T lymphocyte attenuator variant*	79.24
F1LNN2	*B- and T-lymphocyte attenuator*	79.24
Q9JLX9	*Beta actin (Fragment)*	698.76
P15429	*Beta-enolase*	75.52
B5DF65	*Biliverdin reductase B*	77.06
A0A0G2K728	*Cadherin-4*	72.31

P18418	*Calreticulin*	52.46
D3ZP14	*Carboxylic ester hydrolase*	62.81
A0A0G2K455	*Carboxylic ester hydrolase*	116.38
O70177	*Carboxylic ester hydrolase*	67.2
A0A0G2JV37	*Carboxylic ester hydrolase*	53.53
A0A0G2K0C1	*Carboxylic ester hydrolase*	104.49
Q7TP10	*Cc2-36*	90.79
D3ZL71	*Clarin 2*	88.48
P16296	*Coagulation factor IX*	67.62
F1LQ93	*Collagen alpha-1(IX) chain*	93.23
P48199	*C-reactive protein*	78.76
P51952	*Cyclin-dependent kinase 7 (Fragment)*	56.01
Q64680	*Cytochrome P450 2D4*	68.8
D3ZH41	*Cytoskeleton-associated protein 4*	49.3
P80254	*D-dopachrome decarboxylase*	251.41
A0A0F7RQJ6	*D-dopachrome tautomerase*	251.41
G3V6P2	*Dihydrolipoamide S-succinyltransferase (E2 component of 2-oxo-glutarate complex)_ isoform CRA_a*	253.93
Q01205	*Dihydrolipoyllysine-residue succinyltransferase component of 2-oxoglutarate dehydrogenase complex_ mitochondrial*	253.93

Q642E5	*Diphosphomevalonate decarboxylase*	134.05
Q62967	*Diphosphomevalonate decarboxylase*	119.03
P31016	*Disks large homolog 4*	46.85
A0A0G2K7F5	*Disks large homolog 4*	53.99
D0VYQ0	*DLST protein*	142.67
Q5PR00	*DnaJ homolog subfamily C member 22*	112.6
Q7M081	*DnaK-type molecular chaperone P72 (Fragments)*	79.27
Q68G41	*Dodecenoyl-Coenzyme A delta isomerase (3_2 trans-enoyl-Coenzyme A isomerase)*	95.29
D4AD75	*Dpy-19-like C-mannosyltransferase 1*	96.49
M0RD27	*DUF3456 domain-containing protein*	112.71
B5DF83	*Ect2 protein (Fragment)*	104.64
P62632	*Elongation factor 1-alpha 2*	89.41
Q66HD0	*Endoplasmin*	62.27
Q5BJ93	*Enolase 1_ (Alpha)*	91.86
P23965	*Enoyl-CoA delta isomerase 1_ mitochondrial*	95.29
D3ZUD0	*Epithelial cell-transforming 2*	101.51
Q62894	*Extracellular matrix protein 1*	60.11
A0A0H2UHL1	*Fatty acyl-CoA reductase*	114.89

G3V6Z4	*F-box only protein 32*	55.04
F1LRT1	*Fructose-1_6-bisphosphatase 1*	65.52
Q9Z1N1	*Fructose-1_6-bisphosphatase isozyme 2*	83.64
B2GV35	*Galactosylgalactosylxylosylprotein 3-beta-glucuronosyltransferase*	66.86
G3V7N8	*Galectin*	92.1
P47967	*Galectin-5*	92.1
P97840	*Galectin-9*	92.1
Q8CJG5	*Gene*	214.73
A0A0U1RVK4	*Glutamic-oxaloacetic transaminase 1-like 1*	71.91
Q6AY54	*Glutamic-oxaloacetic transaminase 1-like 1*	71.91
Q5BK56	*Glutathione S-transferase*	70.87
P08010	*Glutathione S-transferase Mu 2*	347.96
D4A322	*Glutathione S-transferase_ C-terminal domain-containing*	100.93
P97617	*Glyceraldehyde 3-phosphate dehydrogenase (Fragment)*	564.62
P13255	*Glycine N-methyltransferase*	130.6
D3ZQT0	*Gp_dh_N domain-containing protein*	163.59
Q9EQY6	*GPI mannosyltransferase 1*	68.15
P63018	*Heat shock cognate 71 kDa protein*	97.72
P14659	*Heat shock-related 70 kDa protein 2*	136.09

A0A0G2JYF3	*HECT domain E3 ubiquitin protein ligase 2*	97.76
B5DEN8	*HEPACAM family member 2*	74.19
D3ZU87	*HIG1 domain-containing protein*	85.54
F7EPH4	*Inorganic pyrophosphatase 1*	61.32
A0A0G2JUD2	*Inorganic pyrophosphatase 1*	61.32
D3ZLQ6	*Integrator complex subunit 8*	52.09
Q562A4	*Ipo11 protein (Fragment)*	98.09
Q5BJY4	*Josephin-1*	158.56
F1LM38	*KH domain-containing 4_ pre-mRNA-splicing factor*	71.82
Q5M9H1	*Leucine-rich repeat-containing protein 41*	52.25
P42123	*L-lactate dehydrogenase B chain*	66.72
B2GV26	*LOC304239 protein (Fragment)*	60.38
Q56A23	*LOC361980 protein (Fragment)*	77.46
Q5I0H7	*LOC682097 protein (Fragment)*	173.14
P18163	*Long-chain-fatty-acid--CoA ligase 1*	82.26
Q5M837	*Ly49 stimulatory receptor 6*	114.55
Q4V8A1	*Lysophosphatidylcholine acyltransferase 2B*	79.63
D3ZR52	*Lysophosphatidylcholine acyltransferase 4*	95.44
P57113	*Maleylacetoacetate isomerase*	355.84

A0A0G2K6H2	*Maleylacetoacetate isomerase*	355.84
Q499Q5	*Meckel syndrome type 1 protein homolog*	72.49
M0R976	*Mediator of RNA polymerase II transcription subunit 18*	109.02
D3ZX09	*Metalloendopeptidase*	94.27
Q63335	*MG2 domain-containing protein (Fragment)*	104.99
O89035	*Mitochondrial dicarboxylate carrier*	114.73
A0A0G2K0F5	*Myosin heavy chain 1*	92.38
G3V6E1	*Myosin heavy chain 2*	100.07
Q0GC40	*Myosin heavy chain type IIa (Fragment)*	88.86
A0A0G2K833	*Neuron navigator 2*	133.27
Q9EQK9	*NK cell receptor 2B4*	69.76
G3V8X8	*Noggin*	126.56
D3ZQ51	*Non-specific serine/threonine protein kinase*	76.95
F1M2M6	*NTF2 domain-containing protein*	49.14
Q05982	*Nucleoside diphosphate kinase A*	71.07
P19804	*Nucleoside diphosphate kinase B*	148.13
A0A0G2K4D4	*Olfactory receptor*	67.3
A0A0G2JZA3	*Olfactory receptor*	92.65
D4ACV1	*Olfactory receptor*	121.33

M0R438	*Olfactory receptor*	113.8
F1LW67	*Olfactory receptor*	87
D3ZNH9	*Olfactory receptor*	154.71
D4ABI3	*Olfactory receptor 830*	98.56
Q6AYN7	*Osgepl1 protein (Fragment)*	50.5
H6X2V2	*Pentaxin*	78.76
A0A0G2K2E4	*Peptidyl-prolyl cis-trans isomerase*	127.64
P10111	*Peptidyl-prolyl cis-trans isomerase A*	282
P35704	*Peroxiredoxin-2*	125.23
A0A0G2JUF1	*Peroxisomal trans-2-enoyl-CoA reductase*	130.39
D3ZKT0	*Phosphatidate cytidylyltransferase_ mitochondrial*	91.38
Q5XIV1	*Phosphoglycerate kinase*	78.77
P81062	*Pituitary homeobox 3*	65.39
Q499R7	*Ppa1 protein (Fragment)*	61.32
G3V6N5	*PQ loop repeat containing 3*	76.91
D4ACH7	*Preferentially-expressed antigen in melanoma*	76.85
Q32PY9	*Probable gluconokinase*	73.58
Q4V7F3	*Probable tRNA N6-adenosine threonylcarbamoyltransferase_ mitochondrial*	50.5
P62963	*Profilin-1*	137.26

Q62969	*Prostacyclin synthase*	121.38
P11598	*Protein disulfide-isomerase A3*	90.38
Q4VY99	*Putative tctex1/2 family protein (Fragment)*	144.66
A0A0H2UI07	*Pyruvate kinase*	59.79
P12928	*Pyruvate kinase PKLR*	59.79
M0RB91	*Queuine tRNA-ribosyltransferase accessory subunit 2*	79.03
Q6AYT0	*Quinone oxidoreductase*	101.95
D3ZBM0	*R3H domain-containing-like*	73.98
D4A1C1	*RAB_ member of RAS oncogene family-like 3*	103.54
Q3B7T9	*Rab11 family-interacting protein 1*	76.02
D4A7A8	*Rab7b_ member RAS oncogene family*	121.22
A0A0G2JSK1	*RCG20603*	83.66
G3V8D8	*RCG50308_ isoform CRA_a*	144.66
F7F3I7	*RCG62756_ isoform CRA_a*	99.04
P49803	*Regulator of G-protein signaling 7*	58.41
A0A0G2JVG0	*Regulator of telomere elongation helicase 1*	97.45
Q8R4H5	*Retinoic acid inducible in neuroblastoma cells RAINB1 (Fragment)*	119.14
Q4V889	*Ring finger protein 12*	74.53
A0A096MKG3	*Ring finger protein_ LIM domain interacting*	57.09

B1WBW1	*Rtel1 protein (Fragment)*	97.45
A0A0G2K0H9	*RuvB-like helicase*	84
A0A1B0GWV9	*Semaphorin 4A (Fragment)*	99.04
B0BNE5	*S-formylglutathione hydrolase*	91.09
G3V8P5	*Similar to cDNA sequence BC017158*	72.26
F1LZI1	*Similar to heat shock protein 8*	67.24
Q6J329	*Solute carrier family 25 member 47*	67.04
A0JPP4	*Sperm equatorial segment protein 1*	62.8
A0A0G2K849	*TATA-box binding protein associated factor 4*	190.37
B1WBT5	*Tetratricopeptide repeat domain 8*	67.15
G3V912	*Thioredoxin domain containing 13*	127.41
B1WC39	*Tigger transposable element derived 5*	63.01
M0R6R9	*Titin-like*	163.16
A0A0G2K0Y1	*TMF1-regulated nuclear protein 1*	222.69
Q1XID0	*Transcription factor LBX1*	93.41
M0R5E3	*Transmembrane protein 200C*	88.81
A0A0G2K330	*Trifunctional enzyme subunit beta_ mitochondrial*	58.57
Q4KLZ6	*Triokinase/FMN cyclase*	53.67
A0A0G2JWU1	*Triosephosphate isomerase*	97.66

P70566	*Tropomodulin-2*	74.43
B4F7C2	*Tubulin beta chain*	124.16
Q4QQV0	*Tubulin beta chain*	56.91
G3V7C6	*Tubulin beta chain*	130.09
M0R8B6	*Tubulin beta chain*	68.1
P85108	*Tubulin beta-2A chain*	82.46
Q3KRE8	*Tubulin beta-2B chain*	82.46
Q4QRB4	*Tubulin beta-3 chain*	106.15
Q6P9T8	*Tubulin beta-4B chain*	124.16
P69897	*Tubulin beta-5 chain*	82.46
Q52KK2	*Txndc13 protein (Fragment)*	127.41
Q6T5F3	*UDP-glucuronosyltransferase*	62.15
F1LP67	*Uncharacterized protein*	121.38
A0A0G2JYL7	*Uncharacterized protein*	111.78
A0A1P8YVD0	*Uncharacterized protein*	67.15
M0R5J4	*Uncharacterized protein*	91.86
Q04462	*Valine--tRNA ligase*	722.92
Q91WX9	*Voltage-dependent L-type calcium channel subunit alpha*	25.03
Q5J3L2	*Vomeronasal type-1 receptor*	70.78

A0A0G2KAW5	*WD repeat domain 48*	59.28
Q8VDA5	*Z-DNA-binding protein 1*	104.21
D3Z8I0	*Zinc finger protein 541*	94.2

Table S15. Unique proteins found in liver samples from DEHG rats (animals exposed to 1 mg/kg/day of Dexamethasone and 300 mg/kg/day of HMB for 10 days)

**Accession number**	***Protein name***	***PLGS Score***
O88521	*190 kDa ankyrin isoform*	82.89
Q9JJ46	*3-beta-hydroxysteroid-Delta(8)_Delta(7)-isomerase*	69.38
D3ZSL2	*ABRA C-terminal-like*	61.36
F1M1W1	*Acyl-coenzyme A synthetase ACSM2_ mitochondrial*	49.66
B2RZC0	*Aig1 protein*	79.08
F1LYE2	*Androglobin*	61.45
Q3T1J5	*Ank3 protein*	88.26
Q574E1	*Ankyrin G109*	88.26
O70511	*Ankyrin-3*	96.45
O70510	*AnkyrinG (Fragment)*	66.71
Q5M890	*Apolipoprotein N*	70.04
A0A0G2K847	*ArfGAP with GTPase domain_ ankyrin repeat and PH domain 1*	71.13
M0R5J1	*ArfGAP with SH3 domain_ ankyrin repeat and PH domain 3*	127.5
Q6PCU0	*ATP synthase subunit gamma*	56.28
O88750	*Bilitranslocase*	75.02

Q4V8K5	*BRO1 domain-containing protein BROX*	110.67
B5DFA3	*Bucs1 protein*	68.68
F1M1A2	*Cadherin 12*	64.79
A0A0G2K681	*CAP-Gly domain-containing linker protein 1*	77.86
F1LRN4	*CAP-Gly domain-containing linker protein 4*	52.89
Q8K3R0	*Carboxylic ester hydrolase*	58.61
B0BND8	*Centg2 protein (Fragment)*	71.13
M0RAF2	*Checkpoint protein*	147.05
Q6IRG7	*Claudin*	227.55
Q99P82	*Claudin-11*	227.55
Q4V8P6	*Clip1 protein*	62.29
A0A0U1RRT4	*Coatomer protein complex_ subunit epsilon (Fragment)*	119.71
G3V8Q1	*Coatomer subunit epsilon*	119.71
Q62613	*CREMdeltaC-G*	82.32
A0A0G2K1N8	*Cysteine and glycine-rich protein 3*	127.5
P10818	*Cytochrome c oxidase subunit 6A1_ mitochondrial*	194.65
P10634	*Cytochrome P450 2D26*	72.1
Q63156	*Decorin (Fragment)*	295.73

F1LY78	*Deltex E3 ubiquitin ligase 3*	124.06
Q4QQV2	*Docking protein 1*	82.65
A0A0A0MY02	*E2F transcription factor 7 (Predicted)_ isoform CRA_a*	68.26
F1M4M8	*E3 ubiquitin-protein ligase*	87.3
D4A488	*Elastase 2_ neutrophil (Predicted)*	376.94
Q6P7Q0	*Electron transfer flavoprotein beta subunit lysine methyltransferase*	82.51
B0BMU7	*Elongator complex protein 5*	70.32
D4A8X5	*G protein-coupled receptor 45*	73.29
F1M9H1	*GLI family zinc finger 3*	72.85
B0BN46	*Glyoxylate and hydroxypyruvate reductase*	77.93
P16261	*Graves disease carrier protein (Fragment)*	61.84
Q6MGB4	*H2-K region expressed gene 4_ rat orthologue*	92.65
Q6IMX7	*Hsp70-binding protein 1*	69.14
G3V8Y8	*Huntingtin interacting protein 1_ isoform CRA_a*	58.49
D4A7K4	*Interleukin-1 receptor-associated kinase 4*	88.36
D3ZUL9	*IQ motif-containing E*	47.1
Q6IE12	*Kallikrein 4*	74.02
A0A0G2K522	*KAT8 regulatory NSL complex subunit 3*	67.15
A0A0U1RRN6	*Keratin 78*	71.06

Q63279	*Keratin_ type I cytoskeletal 19*	66.55
Q6LE95	*Kininogen*	84.03
F7EUK4	*Kininogen-1*	84.03
D3ZD05	*KN motif and ankyrin repeat domain-containing protein 2*	64.27
Q5FVK7	*LOC685045 protein*	61.36
D3ZY41	*Makorin_ ring finger protein_ 3*	65.32
M0R5R3	*Mannosyltransferase*	126.82
A0JPR2	*Mapk12 protein*	102.86
W8W3K0	*Mas-related G protein-coupled receptor b*	92.42
Q7TN38	*Mas-related G-protein coupled receptor member H*	92.42
Q9ES73	*Melanoma-associated antigen D1*	178.11
Q63538	*Mitogen-activated protein kinase 12*	96.25
G3V7K1	*Myomesin 2*	96.52
Q5PQM4	*Myosin-binding protein H-like*	163.9
P97546	*Neuroplastin*	77.4
D3ZH90	*NME/NM23 family member 5*	146
A0A0G2K2B4	*Nuclear receptor corepressor 1*	51.36
D4ADW1	*Olfactory receptor*	80.04
D3ZMC8	*Olfactory receptor*	100.24

Q8VHZ4	*Paraneoplastic antigen Ma1 homolog*	84.96
Q7TT47	*Paraplegin*	73.04
D3ZBG6	*Peptidyl-tRNA hydrolase domain-containing 1*	150
A0A0G2JSS8	*Peroxiredoxin*	93.28
Q9R063	*Peroxiredoxin-5_ mitochondrial*	93.28
D4A2Y9	*Peroxisomal biogenesis factor 13*	67.95
P25113	*Phosphoglycerate mutase 1*	96.36
P07633	*Propionyl-CoA carboxylase beta chain_ mitochondrial*	82.69
Q6DGG1	*Protein ABHD14B*	74.36
Q5I034	*Protein CUSTOS*	95.68
Q68HB8	*Protocadherin 7*	65.54
Q68HB5	*Protocadherin 7_ isoform CRA_b*	65.54
Q68HB7	*Protocadherin 7b*	65.54
Q68HB6	*Protocadherin 7c*	65.54
F1LS01	*Protocadherin 9*	123.33
Q00A23	*Protocadherin 9 (Fragment)*	102.71
D4A376	*Ras-related protein Rab-12*	88.55
O55147	*Rattus norvegicus utrophin*	12.6
D4ADT0	*RCG37011*	59.71

G3V8X6	*RCG39455_ isoform CRA_a*	103.35
E9PU34	*RCG41110*	55.71
Q3KRD9	*RGD1311595 protein (Fragment)*	58.47
Q4V8F3	*Rhomboid-related protein 4*	94.41
D3Z8R4	*RNA-binding motif protein 25-like 1*	136.94
F1LPJ2	*Ryanodine receptor 3*	100.43
O35209	*Ryanodine receptor 3 (Fragment)*	96.33
Q6P688	*S-adenosylmethionine synthase*	65.66
A0A0G2JZI0	*Scaffold attachment factor B*	127.5
B5DFD3	*Schip1 protein*	88.62
F1M6Q1	*Schwannomin-interacting protein 1*	88.62
D3ZA76	*Serine protease HTRA3*	74.29
D4A229	*Serine/threonine-protein kinase*	68.82
F1MAA3	*Serine/threonine-protein phosphatase 2A 56 kDa regulatory subunit*	93.71
B5DFD8	*SH3 domain-binding glutamic acid-rich-like protein*	140.2
Q4FZX7	*Signal recognition particle receptor subunit beta*	63.42
D4A3D3	*Similar to RIKEN cDNA 2700097O09*	172.65
D4A6Y6	*Solute carrier family 25 member 26*	73.56
Q6AXW5	*Sp56 protein*	190.39

Q5XIJ9	*Succinyl-CoA:3-ketoacid coenzyme A transferase 2A_ mitochondrial*	87.95
M0RA66	*Takusan domain-containing protein*	55.13
Q9JKT7	*Taste receptor type 2 member 13*	106.63
P08932	*T-kininogen 2*	84.03
D3ZPC3	*TLC domain-containing 2*	103.58
Q812C4	*Translation initiation factor 4A_ isoform 1 (Fragment)*	74.71
Q6QMY6	*Tsukushin*	72.16
A0A0H2UHM7	*Tubulin alpha chain*	64.66
P68370	*Tubulin alpha-1A chain*	64.66
Q6P9V9	*Tubulin alpha-1B chain*	60.02
Q6AYZ1	*Tubulin alpha-1C chain*	64.66
O35803	*Tyrosine-protein kinase (Fragment)*	51.25
A0A0G2K727	*UDP-glucuronosyltransferase*	86.51
D3Z973	*Uncharacterized protein*	71.83
Q5M833	*Wash2 protein (Fragment)*	103.4
D3ZYC1	*Zinc finger protein 507*	124
Q91WY5	*Zinc finger transcription factor GLI3 (Fragment)*	64.39
Q5U1X7	*Zinc transporter ZIP3*	146.75
G3V902	*Zona pellucida 3 receptor_ isoform CRA_a*	190.39

Table S16. Proteins identified in the liver of animals with differential expression in the GED group compared to the GEP group (upregulated or downregulated).

Accession number	***Protein name***	***PLGS Score***	**Protein Expression Ratio DEG:PEG**
M0R8T2	*10-formyltetrahydrofolate dehydrogenase PE=1 SV=2*	177.4	0.576949804
P52759	*2-iminobutanoate/2-iminopropanoate deaminase*	1692	0.449328959
D3ZRN3	*Actin_ beta-like 2*	444.54	0.71177032
P60711	*Actin_ cytoplasmic 1*	5563.04	0.869358235
P63269	*Actin_ gamma-enteric smooth muscle*	4486.41	0.878095435
Q8K571	*ADH-like protein*	709.49	0.571209062
P06757	*Alcohol dehydrogenase 1*	699.32	0.559898376
G3V7J0	*Aldehyde dehydrogenase family 6_ subfamily A1_ isoform CRA_b*	164.39	0.733446954
P11884	*Aldehyde dehydrogenase_ mitochondrial*	668.52	0.537944435
P31210	*Aldo-keto reductase family 1 member D1*	126.33	0.794533599
F1LML3	*Aldo-keto reductase family 1 member D1*	126.33	0.802518799
A1Z0K8	*Beta-actin (Fragment)*	2639.25	0.835270205
Q99NC6	*Beta-actin FE-3 (Fragment)*	1469.69	0.826959136
Q63276	*Bile acid-CoA:amino acid N-acyltransferase*	422.68	0.49658531

P04762	*Catalase*	542.27	0.444858065
P22734	*Catechol O-methyltransferase*	336.89	0.532591804
P18757	*Cystathionine gamma-lyase*	316.38	0.511708569
Q64680	*Cytochrome P450 2D4*	58.78	0.532591804
P28037	*Cytosolic 10-formyltetrahydrofolate dehydrogenase*	176.29	0.58274824
P80254	*D-dopachrome decarboxylase*	774.69	0.594520559
A0A0F7RQJ6	*D-dopachrome tautomerase*	774.69	0.657046828
P06761	*Endoplasmic reticulum chaperone BiP*	411.92	0.778800783
A0A0G2K4I4	*Endoplasmin*	90.29	0.63128364
P02692	*Fatty acid-binding protein_ liver*	2542.69	0.554327299
P25093	*Fumarylacetoacetase*	562.5	0.794533599
A0A0G2K8Q5	*Glutathione S-transferase*	373.56	0.657046828
P00502	*Glutathione S-transferase alpha-1*	418.39	0.677056884
P04903	*Glutathione S-transferase alpha-2*	418.39	0.690734327
P04904	*Glutathione S-transferase alpha-3*	2710.05	0.618783398
P46418	*Glutathione S-transferase alpha-5*	674.53	0.644036423
P04905	*Glutathione S-transferase Mu 1*	2321.41	0.310366955
P08010	*Glutathione S-transferase Mu 2*	1045.98	0.364218983
F1M4A7	*Gse1 coiled-coil protein*	176.89	0.663650253

Q498T5	*Gsta3 protein*	630.98	0.594520559
D4A4S3	*Heat shock cognate 71 kDa protein*	232.33	0.794533599
P63018	*Heat shock cognate 71 kDa protein*	232.33	0.802518799
A0A0G2K7R1	*Histone domain-containing protein*	794.01	0.794533599
D4AEC0	*Histone H2A*	576.82	0.843664815
P02262	*Histone H2A type 1*	1408.33	0.852143792
Q4FZT6	*Histone H2A type 3*	1408.33	0.843664815
Q00728	*Histone H2A type 4*	1408.33	0.843664815
D3ZNZ9	*Histone H2B*	1109.59	0.923116348
Q5I0H7	*LOC682097 protein (Fragment)*	72.76	0.21438111
P18163	*Long-chain-fatty-acid--CoA ligase 1*	169.43	0.511708569
Q6TUG1	*LRRGT00083*	310.83	0.740818212
Q6QI17	*LRRGT00191*	105.49	0.197898698
A0A0G2K6H2	*Maleylacetoacetate isomerase*	273.23	0.726149042
P57113	*Maleylacetoacetate isomerase*	273.23	0.748263574
Q02253	*Methylmalonate-semialdehyde dehydrogenase [acylating]_ mitochondrial*	156.6	0.740818212
O89035	*Mitochondrial dicarboxylate carrier*	139.9	0.481908981
P00481	*Ornithine carbamoyltransferase_ mitochondrial*	1047.68	0.65050909

Q5M897	*Otc protein*	1047.68	0.644036423
A0A0G2K1P0	*Peptidyl-prolyl cis-trans isomerase*	219.23	0.197898698
P10111	*Peptidyl-prolyl cis-trans isomerase A*	219.23	0.182683515
Q63716	*Peroxiredoxin-1*	812.12	0.697676316
Q9Z0V5	*Peroxiredoxin-4*	304.53	0.697676316
Q9WVK3	*Peroxisomal trans-2-enoyl-CoA reductase*	245.25	0.690734327
Q6LDP3	*Rat glutathione S-transferase*	1112.55	0.332871076
B0BMT0	*RCG47746_ isoform CRA_a*	4486.41	0.869358235
Q03336	*Regucalcin*	1019.75	0.313486191
A0A0G2JUM9	*Reverse transcriptase domain-containing protein*	115.02	0.199887611
D3ZD94	*Similar to class-alpha glutathione S-transferase*	310.83	0.733446954
P07632	*Superoxide dismutase [Cu-Zn]*	169.06	0.683861412
A0A0G2K849	*TATA-box binding protein associated factor 4*	91.34	0.236927745
A0A0G2K3Z9	*Thioredoxin domain-containing protein*	812.12	0.697676316
P24329	*Thiosulfate sulfurtransferase*	1115.05	0.625002269
Q6T5F3	*UDP-glucuronosyltransferase*	55.76	0.458406024
P08541	*UDP-glucuronosyltransferase 2B2*	219.39	0.516851321
P09118	*Uricase*	286.34	0.740818212
Q62670	*0 beta-2 globin*	246.37	3.596639623

Q68G44	*3-hydroxy-3-methylglutaryl coenzyme A synthase*	135.5	1.491824707
P63039	*60 kDa heat shock protein_ mitochondrial*	636.17	1.173510867
Q7TMC7	*Ab2-417*	264.97	1.716006899
Q7TPI5	*Ac2-281*	494.52	1.584073998
P10760	*Adenosylhomocysteinase*	190.26	2.316366916
Q63910	*Alpha globin*	66.3	4.349235265
P07824	*Arginase-1*	539.7	1.537257535
P09034	*Argininosuccinate synthase*	375.83	1.768267039
P13221	*Aspartate aminotransferase_ cytoplasmic*	87.46	4.806648446
Q0QEP3	*ATP synthase subunit beta (Fragment)*	2228.7	1.072508182
P10719	*ATP synthase subunit beta_ mitochondrial*	2607.68	1.094174288
Q7TP24	*Ba1-667*	258.56	1.716006899
A0A0G2JTW9	*Beta globin minor gene*	372.62	3.254374032
O09171	*Betaine--homocysteine S-methyltransferase 1*	2597.96	2.117000017
P07756	*Carbamoyl-phosphate synthase [ammonia]_ mitochondrial*	1665.12	1.197217372
O88752	*Epsilon 1 globin*	291.08	3.525421454
P12785	*Fatty acid synthase*	61.76	2.095935534
P00884	*Fructose-bisphosphate aldolase B*	596.73	1.584073998
Q62669	*Globin a1*	372.62	3.35348478

A0A1K0FUA6	*Globin a2*	1580.79	3.669296493
A0A0G2JSW3	*Globin a4*	2973.28	3.596639623
A0A0G2JSV6	*Globin c2*	6134.58	3.2219925
G3V8R3	*Globin e1*	66.3	4.85495602
P10860	*Glutamate dehydrogenase 1_ mitochondrial*	983.61	1.209249595
P04797	*Glyceraldehyde-3-phosphate dehydrogenase*	2461.76	1.336427477
P13255	*Glycine N-methyltransferase*	77.73	2.435129616
A0A0G2JYY8	*Gp_dh_N domain-containing protein*	353.78	1.404947596
P01946	*Hemoglobin subunit alpha-1/2*	6134.58	3.2219925
P11517	*Hemoglobin subunit beta-2*	1580.79	3.669296493
A0A482IDN3	*Hsp60*	636.17	1.138828378
P22791	*Hydroxymethylglutaryl-CoA synthase_ mitochondrial*	135.5	1.491824707
P12007	*Isovaleryl-CoA dehydrogenase_ mitochondrial*	86.59	1.377127754
P04642	*L-lactate dehydrogenase A chain*	558.98	1.447734622
O88989	*Malate dehydrogenase_ cytoplasmic*	217.99	2.117000017
P52873	*Pyruvate carboxylase_ mitochondrial*	160.61	1.349858824
P13444	*S-adenosylmethionine synthase isoform type-1*	143.45	1.682027618
P12346	*Serotransferrin*	264.97	1.69893226
P02770	*Serum albumin*	195.79	2.857650982

Q68FT5	*S-methylmethionine--homocysteine S-methyltransferase BHMT2*	662.55	2.181472203
P17988	*Sulfotransferase 1A1*	211.58	1.87761057
Q64428	*Trifunctional enzyme subunit alpha_ mitochondrial*	201.73	1.521961536
Q63011	*Zero beta-globin (Fragment)*	372.62	3.387187831

Ratios with values lower than 1 represent downregulated proteins. whereas ratio values greater than 1 correspond to upregulated proteins. The identified proteins are divided into downregulated proteins at the top of the table and upregulated proteins at the bottom of the table.

Table S17. Proteins identified in the liver of animals with differential expression in the DEHG group compared to the PEG group (upregulated or downregulated).

Accession number	***Protein name***	***PLGS Score***	**Protein Expression Ratio DEHG:PEG**
P52759	*2-iminobutanoate/2-iminopropanoate deaminase*	1692	0.301194198
P68136	*Actin_ alpha skeletal muscle*	4486.41	0.748263574
D3ZRN3	*Actin_ beta-like 2*	444.54	0.594520559
P60711	*Actin_ cytoplasmic 1*	5563.04	0.740818212
Q8K571	*ADH-like protein*	709.49	0.522045789
P06757	*Alcohol dehydrogenase 1*	699.32	0.50157607
A0A0G2JW98	*Alcohol dehydrogenase 6*	265.9	0.49658531
G3V7J0	*Aldehyde dehydrogenase family 6_ subfamily A1_ isoform CRA_b*	164.39	0.740818212
P11884	*Aldehyde dehydrogenase_ mitochondrial*	668.52	0.436049294
P15999	*ATP synthase subunit alpha_ mitochondrial*	452.88	0.869358235
A0A068F1Y2	*Beta-actin (Fragment)*	3758.33	0.58274824
Q63276	*Bile acid-CoA:amino acid N-acyltransferase*	422.68	0.323033258
P04762	*Catalase*	542.27	0.241714027
F2W8B0	*Catechol O-methyltransferase*	336.89	0.398519034

P18757	*Cystathionine gamma-lyase*	316.38	0.477113911
P28037	*Cytosolic 10-formyltetrahydrofolate dehydrogenase*	176.29	0.511708569
P62630	*Elongation factor 1-alpha 1*	181.32	0.778800783
P06761	*Endoplasmic reticulum chaperone BiP*	411.92	0.677056884
P14604	*Enoyl-CoA hydratase_ mitochondrial*	261.84	0.543350861
P02692	*Fatty acid-binding protein_ liver*	2542.69	0.491644208
P25093	*Fumarylacetoacetase*	562.5	0.726149042
P00502	*Glutathione S-transferase alpha-1*	418.39	0.367879441
P04905	*Glutathione S-transferase Mu 1*	2321.41	0.26184566
A0A0G2K7R1	*Histone domain-containing protein*	794.01	0.71177032
D3ZXP3	*Histone H2A*	576.82	0.794533599
D3ZWM5	*Histone H2B*	1235.4	0.904837417
Q10758	*Keratin_ type II cytoskeletal 8*	444.39	0.826959136
Q6TUG1	*LRRGT00083*	310.83	0.272531806
Q02253	*Methylmalonate-semialdehyde dehydrogenase [acylating]_ mitochondrial*	156.6	0.733446954
P00481	*Ornithine carbamoyltransferase_ mitochondrial*	1047.68	0.704688094
Q63716	*Peroxiredoxin-1*	812.12	0.527292432
Q9Z0V5	*Peroxiredoxin-4*	304.53	0.565525443

Q9WVK3	*Peroxisomal trans-2-enoyl-CoA reductase*	245.25	0.657046828
M0R6Y8	*Phosphoglycerate kinase*	207.8	0.71177032
Q63030	*Rat alpha-smooth muscle actin mRNA (Fragment)*	2862.04	0.576949804
Q6LDP3	*Rat glutathione S-transferase*	1112.55	0.175520399
Q63232	*Rat liver glutathione S-transferase Ya subunit mRNA (Fragment)*	119.44	0.298197268
B0BMT0	*RCG47746_ isoform CRA_a*	4486.41	0.755783741
Q03336	*Regucalcin*	1019.75	0.201896513
A0A0G2K3Z9	*Thioredoxin domain-containing protein*	812.12	0.522045789
P24329	*Thiosulfate sulfurtransferase*	1115.05	0.49658531
P50137	*Transketolase*	320.32	0.818730751
F1M6C2	*Tr-type G domain-containing protein*	181.32	0.786627865
A0A0G2K727	*UDP-glucuronosyltransferase*	60.76	0.236927745
Q68G19	*UDP-glucuronosyltransferase*	79.41	0.310366955
Q6T5E8	*UDP-glucuronosyltransferase*	55.76	0.467666431
P09118	*Uricase*	286.34	0.697676316
Q62670	*0 beta-2 globin*	246.37	1.768267039
Q7TMC7	*Ab2-417*	264.97	1.271249144
Q6P743	*Adenosylhomocysteinase*	190.26	2.203396474
Q63910	*Alpha globin*	66.3	3.560852494

P07824	*Arginase-1*	539.7	1.682027618
P09034	*Argininosuccinate synthase*	375.83	1.309964465
P13221	*Aspartate aminotransferase_ cytoplasmic*	87.46	5.002811299
Q7TP24	*Ba1-667*	258.56	1.309964465
A0A0G2JTW9	*Beta globin minor gene*	372.62	1.896480852
O09171	*Betaine--homocysteine S-methyltransferase 1*	2597.96	2.410899695
O88752	*Epsilon 1 globin*	291.08	1.221402762
P12785	*Fatty acid synthase*	61.76	2.203396474
Q66HT1	*Fructose-bisphosphate aldolase*	700.98	1.786038401
P00884	*Fructose-bisphosphate aldolase B*	596.73	1.87761057
Q62669	*Globin a1*	372.62	1.858928051
A0A1K0FUA6	*Globin a2*	1580.79	1.221402762
A0A0G2JSW3	*Globin a4*	2973.28	1.271249144
A0A0G2JSV6	*Globin c2*	6134.58	1.750672504
P10860	*Glutamate dehydrogenase 1_ mitochondrial*	983.61	1.185304853
M0R660	*Glyceraldehyde-3-phosphate dehydrogenase*	2461.76	1.1502738
P16261	*Graves disease carrier protein (Fragment)*	117.4	7.028687916
P01946	*Hemoglobin subunit alpha-1/2*	6134.58	1.197217372
P02091	*Hemoglobin subunit beta-1*	2897.92	1.258600015

P11517	*Hemoglobin subunit beta-2*	1580.79	1.221402762
B5DEN4	*L-lactate dehydrogenase*	558.98	1.233678052
P04642	*L-lactate dehydrogenase A chain*	558.98	1.221402762
Q0QF43	*Malate dehydrogenase (Fragment)*	409.6	1.258600015
O88989	*Malate dehydrogenase_ cytoplasmic*	217.99	2.270499821
F1LRA5	*Proteoglycan 4*	126.53	1.803988368
P52873	*Pyruvate carboxylase_ mitochondrial*	160.61	1.271249144
P13444	*S-adenosylmethionine synthase isoform type-1*	143.45	1.552707215
P12346	*Serotransferrin*	264.97	1.284025417
A0A0G2JSH5	*Serum albumin*	229.82	1.822118844
P02770	*Serum albumin*	195.79	1.896480852
Q68FT5	*S-methylmethionine--homocysteine S-methyltransferase BHMT2*	662.55	3.287081395
Q9JKL7	*Splicing regulatory glutamine/lysine-rich protein 1*	123.38	3.819043633
P17988	*Sulfotransferase 1A1*	211.58	2.410899695
Q64428	*Trifunctional enzyme subunit alpha_ mitochondrial*	201.73	1.682027618
Q63011	*Zero beta-globin (Fragment)*	372.62	1.858928051

Ratios with values lower than 1 represent downregulated proteins. whereas ratio values greater than 1 correspond to upregulated proteins. The identified proteins are divided into downregulated proteins at the top of the table and upregulated proteins at the bottom of the table.

Table S18. Proteins identified in the liver of animals with differential expression in the GEDH group compared to the GED group (upregulated or downregulated).

Accession number	***Protein name***	***PLGS Score***	**Protein Expression Ratio DEHG:DEG**
Q62670	*0 beta-2 globin*	120.33	0.543350861
P63259	*Actin_ cytoplasmic 2*	2029.11	0.93239382
Q63910	*Alpha globin*	111.78	0.726149042
A0A0G2JTW9	*Beta globin minor gene*	448.11	0.677056884
A0A068F1Y2	*Beta-actin (Fragment)*	790.89	0.704688094
Q6PDU6	*Beta-glo*	448.11	0.644036423
P04762	*Catalase*	83.38	0.644036423
O88752	*Epsilon 1 globin*	1337.86	0.199887611
Q62669	*Globin a1*	448.11	0.625002269
A0A1K0FUA6	*Globin a2*	5606.06	0.339595511
A0A0G2JSW3	*Globin a4*	6255.02	0.458406024
A0A0G2JSV6	*Globin c2*	5695.37	0.93239382
B1H216	*Globin c3*	5695.37	0.93239382
A0A0G2JTB1	*Glutathione S-transferase*	91.52	0.612626388

A0A0G2K8Q5	*Glutathione S-transferase*	91.52	0.618783398
P00502	*Glutathione S-transferase alpha-1*	91.52	0.588604987
P04903	*Glutathione S-transferase alpha-2*	91.52	0.60653066
P14942	*Glutathione S-transferase alpha-4*	628.59	0.444858065
Q4FZZ3	*Glutathione S-transferase alpha-5*	91.52	0.60653066
Q9JLQ6	*Glutathione transferase A3 subunit (Fragment)*	139.57	0.618783398
A0A0G2K8S2	*Gp_dh_N domain-containing protein*	217.54	0.835270205
Q498T5	*Gsta3 protein*	91.52	0.718923724
P01946	*Hemoglobin subunit alpha-1/2*	5695.37	0.554327299
P02091	*Hemoglobin subunit beta-1*	6113.23	0.458406024
P11517	*Hemoglobin subunit beta-2*	5606.06	0.449328959
A0A0G2K4H7	*Keratin 78*	60.68	0.440431658
Q6TUG1	*LRRGT00083*	628.59	0.440431658
Q63716	*Peroxiredoxin-1*	154.48	0.826959136
Q63030	*Rat alpha-smooth muscle actin mRNA (Fragment)*	2373.25	0.594520559
Q63232	*Rat liver glutathione S-transferase Ya subunit mRNA (Fragment)*	628.59	0.427414922
Q03336	*Regucalcin*	145.16	0.718923724
P02770	*Serum albumin*	275.12	0.818730751
D3ZD94	*Similar to class-alpha glutathione S-transferase*	674.91	0.444858065

A0A0G2K3Z9	*Thioredoxin domain-containing protein*	154.48	0.826959136
Q63011	*Zero beta-globin (Fragment)*	448.11	0.618783398
M0R8T2	*10-formyltetrahydrofolate dehydrogenase*	89.13	1.433329435
P23457	*3-alpha-hydroxysteroid dehydrogenase*	239.76	1.185304853
G3V9U2	*3-ketoacyl-CoA thiolase_ mitochondrial*	638.65	1.138828378
A0A0G2K642	*3-ketoacyl-CoA thiolase_ mitochondrial*	638.65	1.1502738
P13437	*3-ketoacyl-CoA thiolase_ mitochondrial*	638.65	1.1502738
Q6P743	*Adenosylhomocysteinase*	236.2	1.233678052
P10760	*Adenosylhomocysteinase*	236.2	1.233678052
G3V7J0	*Aldehyde dehydrogenase family 6_ subfamily A1_ isoform CRA_b*	141.22	1.197217372
P31210	*Aldo-keto reductase family 1 member D1*	124.8	1.246076729
F1LML3	*Aldo-keto reductase family 1 member D1*	124.8	1.271249144
A0A0G2KAV5	*Aldo-keto reductase family 1 member D1*	124.8	1.271249144
P07824	*Arginase-1*	404.42	1.197217372
P09034	*Argininosuccinate synthase*	468.08	1.094174288
P13221	*Aspartate aminotransferase_ cytoplasmic*	436.51	1.10517092
O09171	*Betaine--homocysteine S-methyltransferase 1*	2675.75	1.16183425
A0A0G2JSK9	*Betaine--homocysteine S-methyltransferase 1*	2675.75	1.16183425
G3V9D8	*Carboxylic ester hydrolase*	131.42	1.296930074

O70631	*Carboxylic ester hydrolase*	131.42	1.296930074
M0RC65	*Cofilin 2*	79.19	1.87761057
P02692	*Fatty acid-binding protein_ liver*	2598.1	1.138828378
Q66HT1	*Fructose-bisphosphate aldolase*	544.24	1.296930074
P00884	*Fructose-bisphosphate aldolase B*	531.1	1.309964465
P10860	*Glutamate dehydrogenase 1_ mitochondrial*	562.62	1.10517092
A0A0H2UHM3	*Haptoglobin*	150.63	1.476980773
P06866	*Haptoglobin*	150.63	1.491824707
D3ZNZ9	*Histone H2B*	590.08	1.138828378
G3V9C7	*Histone H2B*	590.08	1.138828378
M0R4L7	*Histone H2B*	590.08	1.1502738
M0RBQ5	*Histone H2B*	590.08	1.1502738
A0A0G2JXI9	*Histone H2B*	590.08	1.1502738
D4A817	*Histone H2B*	590.08	1.1502738
G3V8B3	*Histone H2B*	590.08	1.1502738
D3ZNH4	*Histone H2B*	590.08	1.1502738
D3ZLY9	*Histone H2B*	590.08	1.1502738
D3ZWM5	*Histone H2B*	590.08	1.1502738
A0A0G2JXE0	*Histone H2B*	590.08	1.16183425

Q00715	*Histone H2B type 1*	590.08	1.1502738
Q00729	*Histone H2B type 1-A*	208.87	1.197217372
Q0QF43	*Malate dehydrogenase (Fragment)*	280.49	1.173510867
P04636	*Malate dehydrogenase_ mitochondrial*	366.69	1.221402762
P00481	*Ornithine carbamoyltransferase_ mitochondrial*	577.18	1.173510867
Q5M897	*Otc protein*	577.18	1.185304853
F1LMG2	*S-methylmethionine--homocysteine S-methyltransferase BHMT2*	594.68	1.094174288
Q68FT5	*S-methylmethionine--homocysteine S-methyltransferase BHMT2*	594.68	1.094174288
P17988	*Sulfotransferase 1A1*	270.1	1.246076729
P07632	*Superoxide dismutase [Cu-Zn]*	371.79	1.568312167
Q6LDS4	*Superoxide dismutase [Cu-Zn]*	371.79	1.632316236
Q64428	*Trifunctional enzyme subunit alpha_ mitochondrial*	171.61	1.246076729

Ratios with values lower than 1 represent downregulated proteins. whereas ratio values greater than 1 correspond to upregulated proteins. The identified proteins are divided into downregulated proteins at the top of the table and upregulated proteins at the bottom of the table.

Table S19. Mean values of fiber area and minimum diameter of soleus and EDL muscles.

	Fiber area			
	Soleus		EDL	
	Area (µm²)	SD	Area (µm²)	SD
GCP	2671.854	± 223.205	1826.862	± 157.088
GED	1860.559[a]	± 365.302	1062.340[a]	± 155.508
GEDH	2385.533	± 401.243	1188.613[a]	± 586.142
	Minimum diameter			
	Soleus		EDL	
	MD (µm)	SD	MD (µm)	SD
GCP	45.481	1.885	37.354	1.814
GED	37.021[a]	3.817	28.027[a]	2.113
GEDH	42.782	4.117	27.886[a]	11.902

* Different letters indicate statistically significant difference
